# Omnivory of an Insular Lizard: Sources of Variation in the Diet of *Podarcis lilfordi* (Squamata, Lacertidae)

**DOI:** 10.1371/journal.pone.0148947

**Published:** 2016-02-12

**Authors:** Ana Pérez-Cembranos, Alicia León, Valentín Pérez-Mellado

**Affiliations:** Department of Animal Biology, University of Salamanca, Salamanca, Spain; University of Naples, ITALY

## Abstract

Through 17 years and from a sample of 7,790 faecal pellets and 26,346 prey items, we studied the diet of the Balearic lizard *Podarcis lilfordi* in Aire Island (Menorca, Balearic Islands, Spain). We analysed the diet in terms of prey frequencies, as well as by their volume and biomass contributions. The diet of the Balearic lizard was extremely variable through the years, months and areas under study. The dominance of small clumped prey, particularly ants, was confirmed. However, the main contribution by volume corresponded to beetles, with a relevant role for Diplopoda and terrestrial Isopoda during some months and at particular areas of the island. Several prey items were probably captured at the base of shrubs, under stones or inside rock crevices. Therefore, our estimations of electivity would only be reliable for epigeal and flying prey. The capacity of the Balearic lizard to include marine subsidies in its diet, such as coastal crustaceans, is noteworthy. Also, its consumption of carrion from carcasses of gulls and rabbits and leftovers from human visitors is remarkable. Juvenile conspecifics can also be a sporadic food resource, especially during the second half of summer, whereas the consumption of vegetal matter is constant for each whole year. The shifts of vegetal exploitation among areas of the island and months take place according to availability of different plant species at each area or during a given period. Thus, lizards are able to conduct a thorough monitoring of plant phenology, exploiting a large variety of plant species. Omnivory does not imply the indiscriminate inclusion of any edible food in its diet. Rather, the inclusion of several food items means the adoption of a wide range of foraging behaviours adapted to the exploitation of each food resource.

## Introduction

Determinants of lizard's diets are complex and involve, among other factors, the interplay of evolutionary history, body size, microhabitat characteristics and prey availability [1]. Variation in dietary characteristics among species drives several ecological and evolutionary processes. In addition, diets in islands can be exceedingly different than in continents, because island life deviates in many ways from mainland ecological conditions [2].

The Balearic lizard, *Podarcis lilfordi*, is an active forager. In Mediterranean lizards, the plesiomorphic condition was insectivory, but omnivory was adopted by several species, incorporating vegetal matter and nutrients from other sources in their diet [3–5]. However, the extent of omnivory and its relation to insular characteristics and particular traits of lizard populations still remain largely unknown.

Dietary studies of *P*. *lilfordi* started with Salvador [6], who summarized observations from several populations. Then, a study of Menorcan coastal islets carried out by Pérez-Mellado [7] and Pérez-Mellado and Corti [3], provided a general account of the diets of several lacertid lizards from islands within the Mediterranean (see also [8, 9]). These first studies were based either on stomach contents from captured specimens [6,7] or from a mixture of recently captured lizards and gut contents from museum specimens [3].

According to these previous studies, the diet of the Balearic lizard was based on some common clumped prey such as ants (Formicidae), homopteran bugs, and beetles. The inclusion of a large amount of ants in the diet was interpreted as the result of a long-term adaptation of Balearic lizards to arid environments [3]. In fact, Balearic Islands are characterized by a poor and unpredictable food supply for ectotherms [10,11], a supposed prerequisite for the so-called reversed island syndrome [12]. Thus, the general characteristics of trophic ecology of lizards from the Balearic Islands were explained by a combination of factors, including both historical events and present day ecological conditions of insular environments [13]. Although the main prey types are usually similar, there are some differences in diet and foraging behavior according to the season [3] or the blooming period of some plants [14]. Also, some prey may be consumed at a general low rate, or just in some periods of the year. All these factors made it necessary the analysis of large sample sizes along several years and season [15].

A comparison of the ratios of total energy expenditure during the activity period versus resting metabolism indicated that activity intensity was 73% higher in *P*. *lilfordi* than in other previously studied lizards [10]. Consequently, the Balearic lizard showed an intense activity throughout the daily activity period, probably as a result of active foraging rather than territorial defense. Brown et al. [10] proposed that extremely high population densities, together with poor trophic resources [11], would obligate lizards to an intensive foraging throughout the day in order to maintain a suitable energy balance.

In this work we studied the diet of the Balearic lizard in Aire Island along a period of 17 years. We tried to disentangle the factors accounting for the observed variability along different years, among different seasons and in five different areas of Aire Island.

## Materials and Methods

### Area and species under study

Lizards were studied thanks to special permits from the Servei de Protecció d'Especies, Conselleria de Medi Ambient, Balearic Government (permits numbers: CAP 04/2008 and CAP 09/2010). The studies reported in this paper complied with the current laws of the country (Spain) where they were performed. In addition, all research was conducted in compliance with ethical and welfare standards and procedures of the Bioethics Committee of the University of Salamanca.

**Fig 1:**
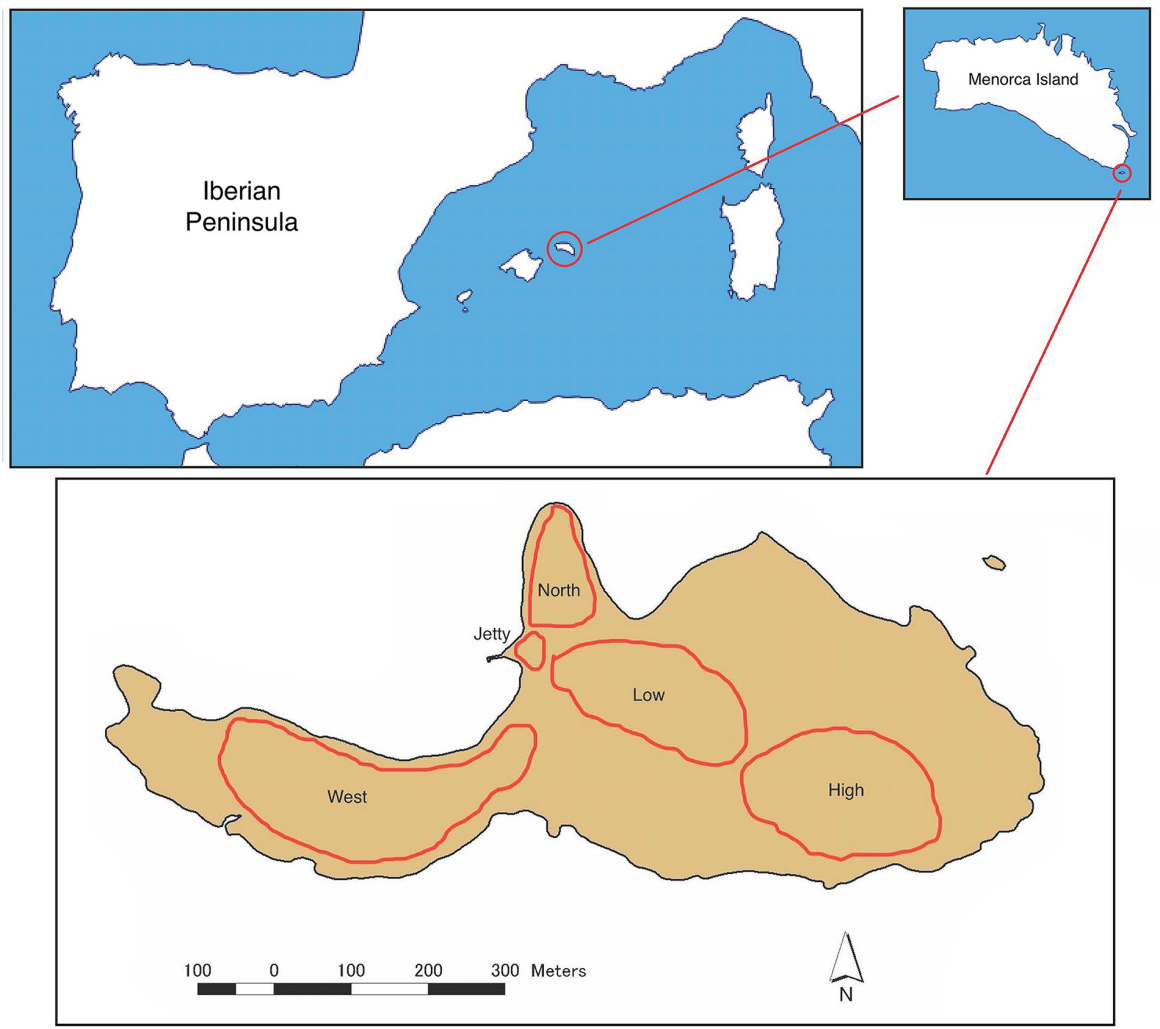
Aire Island. Approximate limits of the five areas under study (see more details in the text).

We conducted this study on Aire Island, a 32 hectares islet off the coast of Menorca. We divided Aire Island into five areas that we designated as High, Low, North, West and Jetty areas (Fig 1). These areas showed differences in plant and rock cover, as well as in lizard densities ([16] and unpub. results).

*P*. *lilfordi* is a lacertid lizard endemic to the Cabrera Archipelago and the offshore islets of Mallorca and Menorca (Balearic Islands, Spain). It is medium-sized with a maximum SVL (snout—vent length) of 81 mm in males and 75 mm in females [17]. *P*. *lilfordi* reaches high population densities on Aire [16].

### Fieldwork and diet study

Scats were obtained directly from the ground or from captured lizards that defecated during handling. Lizards were captured using a noose and were sexed, weighed and measured prior to their release at the exact point of capture. We employed a dataset of 7,790 faecal samples obtained during 17 different years (1995, 1996, 1997, 1999, 2000, 2001, 2002, 2003, 2005, 2006, 2007, 2008, 2009, 2010, 2011, 2012 and 2013). Unfortunately, these annual samples were unbalanced, with only large samples for some months and in only some years. Thus, for some analyses, we worked with representative subsets of the whole dataset. For instance, the comparison among different areas of Aire Island was only made for 2009, 2011 and 2012, because in these three years we obtained large samples of the five areas and also the three months of highest lizard activity: May, June and July. Then, the so-called High area was employed so as to compare annual variation of the diet during the months of April, May, June, July and August (Table 1).

**Table 1:** Sampling over time and its use in the different analyses.

Type of analysis	1995	1996	1997	1999	2000	2001	2002	2003	2005	2006	2007	2008	2009	2010	2011	2012	2013
General	*	*	*	*	*	*	*	*	*	*	*	*	*	*	*	*	*
Annual diet variability													*		*	*	
High area, April										*		*	*	*			*
High area, May										*	*	*	*		*	*	
High area, June											*	*	*	*	*	*	
High area, July		*								*	*	*		*	*	*	*
High area, August	*		*		*					*			*	*		*	
Diptera	*	*	*	*	*	*	*	*	*	*	*	*	*	*	*	*	*
Carrion	*	*	*	*	*	*	*	*	*	*	*	*	*	*	*	*	*
Cannibalism	*	*	*	*	*	*	*	*	*	*	*	*	*	*	*	*	*
Vegetal		*															
*H*. *muscivorus*	*	*	*	*	*	*	*	*	*	*	*	*	*	*	*	*	*

Type of analysis: General, description of the general diet of *P*. *lilfordi* on Aire Island; Annual diet variability, studied with samples from three months of three years, in five areas of the island; High area April to High area August, samples used to compare diet variability in the same area and month along different years; Diptera, analysis of Diptera consumption among areas and months; Carrion, analysis of carrion consumption; Cannibalism, analysis of *Podarcis* consumption; Vegetal; taxonomic analysis of plant consumption; *H*. *muscivorus*, analysis of consumption of fruits of *H*. *muscivorus*.

### Prey availability

To estimate prey availability, we employed the so-called biocenometer [18]. It is a device for quantifying epigeal terrestrial arthropods and other invertebrates. Our biocenometer was a bottomless cube of 1 m side, whose sides and top were covered with a fine netting mesh. Two people quickly placed the biocenometer on the test plot. In this way, it was possible to obtain a good sample of a 1 m^3^ of volume of both epigeal and flying insects. Trapped animals were then removed with entomological forceps, by hand or with a suckling flask, also including invertebrates found under stones. Unfortunately, this method is only useful for epigeal and flying prey types (see below). In the islet under study, only a small fraction of stones can be turned over to search for other invertebrate groups [19]. Hence, a large fraction of hypogeal fauna living under large stones and crevices remained improperly sampled. In May 2009 and June 2011 we collected five samples with the biocenometer at each area of Aire Island (see below).

We tried to describe prey selection with the selectivity index (*D*) of Ivlev [20], later modified by Jacobs [21]:

D=(r−p)/(r+p−2rp)

Where *r* is the proportion of a given prey type in lizard diet and *p* is its proportion in the environment. We also calculated the electivity index (E*) of Vanderploeg and Scavia [22] that would give a better estimation of electivity in the case of small sample sizes:

$$E^{*}{}_{i}=\left[W_{i}-\left(1/n\right)\right]/\left[W_{i}+\left(1/n\right)\right]$$

Where *n* is the number of available prey types and the selectivity coefficient $W_{i}=\left(r_{i}/p_{i}\right)/\sum_{i}\left(r_{i}/p_{i}\right)$, is based on the proportions of prey *i* in the diet (*r*_*i*_) and in the environment (*p*_*i*_).

Both indices range from –1 (total avoidance) through 0 (no or random selection) to +1 (maximum positive selection).

### Laboratory work

We analysed faecal samples under a binocular dissecting microscope. In lizards, diet reconstruction based on a meticulous faecal pellet analysis has been found to be highly comparable to those diet reconstructions based on gastric contents removed from dissected stomachs, with soft-bodied prey and particularly insect larvae being equally represented in faecal pellets and gut contents [23]. Furthermore, faecal pellet analysis is a standard method to quantify diet with the added advantage of not compromising animal well-being (e.g. [24–28]).

Each individual scat was spread in a thin layer of less than 0.5 mm over the entire surface of a Petri dish with some drops of 70° ethylic alcohol. The percentage of vegetal matter was then visually estimated according to the surface occupied by vegetal remains. Plants were recognized up to species, genus or family level only using a subset of 371 scats collected during 1995, 1996 and 1997. From the vegetal fraction of each scat in this subsample, we randomly picked five pinches of plant matter, and then laid them out on microscopic slides. We poured some drops of Hertwig' solution into the slide, flame heating it until the emergence of vapours [29]. This treatment destroyed cell contents preserving cellulose membranes [30]. The slide was finally mounted with Canada balsam for microscopic identification. Further plant recognition was based on leaf epidermis morphology which included the shape of epidermal cells, shape and extension of trichomes, hairs and stomata, as well as the morphology of pollen grains [31]. Samples from scats were compared with a previously made collection of microscopic slides from tissues of the most common plant species of Aire Island and with the aid of some palynological works [32–34] to identify pollen grains. Plant families were arranged according to Cronquist [35] and APG III [36].

Prey remains were identified up to its order or, exceptionally, family level. Prey number for each faecal pellet was conservatively estimated by counting only easily identifiable remains. The consumption of carcasses from birds and mammals was detected either by the presence of feathers or hairs, employing the work of Teerink [37] for hair identification. To estimate prey size, we measured with a micrometer eyepiece the length of intact or nearly intact prey items or of some particular anatomical pieces.

The diet was described according to the three most common methods: as the contribution of each prey item by numbers, as the contribution in biomass (dry weight) and as the contribution by volume. We calculated prey abundance (%n) as the percentage of a given prey type in relation to the total prey number, and the relative prey or plant presence (%p) as the percentage of faeces containing a given type of prey or plant. The percentage of estimated biomass (%b) is the proportion of dry weight of each prey type relative to the total estimated biomass.

To estimate the dry mass of each prey, we employed regression equations for each taxonomic group (for details see [38–41]). Where possible, we applied the regression equations we had previously developed by using arthropods collected in the study area. When no such equation was available for a prey group, we adopted a suitable substitute from Hódar [41,42] or Díaz and Díaz [40]. When prey items were indeed identified but its measurement were not possible, we assigned them the value of average dry mass recorded for that group in the study area. When we could not even identify the group of arthropod remains, we assigned the average dry mass value recorded in faeces for all arthropod groups found. We only made statistical analyses of prey mass for those prey groups whose estimated dry mass values were available. For consumed lizards, biomass was calculated from our own data of hatchlings from Aire Island. For Gastropoda, biomass was calculated by measuring 60 snails and then using the equations provided by Collins [43].

The volume of large prey items was estimated by its displacement in alcohol. It was the case of Isopoda, other Crustacea and Gastropoda. For the remaining prey items, volumes were calculated using the formula of a prolate spheroid [44] excepting for Diplopoda, where volumes were estimated using the formula of a cylinder. Measurements used for calculating volumes were directly taken from prey items found in faecal pellets, excepting in those prey types that were extremely fragmented. In this case, we employed either specimens from samples of availability (see below) or preserved specimens of each prey type: Isopoda (genus *Armadilidium*, n = 30 and genus *Oniscus* n = 30, Oniscidae), Blattodea (n = 27), Araneae (n = 8), Pseudoscorpionida (n = 15), Collembola (n = 30), Isoptera (n = 30), Dermaptera (genus *Forficula*, n = 30), Diplopoda (genus *Julus*, n = 17 and genus *Polidesmus*, n = 2). For 'other Crustacea' we measured the most common crab species from Aire Island shores, the marbled rock crab, *Pachygrapsus marmoratus* (n = 9), because we had evidence of its consumption by lizards, from direct observations. For Gastropoda, we employed 7 empty shells of Hellicidae. All preserved specimens were obtained from the Zoological Collection of Animal Biology Department of the University of Salamanca. All volumes were expressed in cm^3^. Average volumes of each prey type were applied to frequency tables, to make an estimation of the contribution of each prey type by volume.

### Statistical procedures

To study the variation of diets attributed to different covariates, we employed a permutational multivariate analysis of variance [45] using the 'adonis' function from 'vegan' R package [46]. In addition, the multivariate homogeneity of group dispersions (variances) was tested with the function 'betadisper' from 'vegan' package, a multivariate analogue of Levene's test for homogeneity of variances.

We estimated and compared diet diversities using the approach proposed by Pallmann et al. [47]. Instead describing diet diversity through a given index as, for example, Simpson or Shannon indices, we converted these "raw" indices into "true" diversities which all belong to one and the same mathematical family. That is, regarding different measures as special cases of Hill's general definition of diversity measures [48]. In this way, to study differences in diversity between the diet and availability, or among diets of different years, months, seasons or sites, we performed two-tailed tests for integral Hill numbers of orders -1≤*q*≤3. This selection includes the transformed versions of the three following indices: the species richness index, *H*_*sr*_ (*q* = 0), the Shannon entropy index, *H*_*sh*_ (*q→*1) and the Simpson concentration index, *H*_*si*_ (*q* = 2) [49]. All comparisons among diversities of groups were made with Tukey-like contrasts employing a resampling procedure. We did 5000 bootstrap replications so as to obtain reliable p-values [50]. Methods described here are implemented in R package “simboot” [51] and are fully described in Pallmann et al. [47]. All calculations were done in R version 3.0.3 [52].

## Results

### Diet of *P*. *lilfordi* in Aire Island

**Fig 2:**
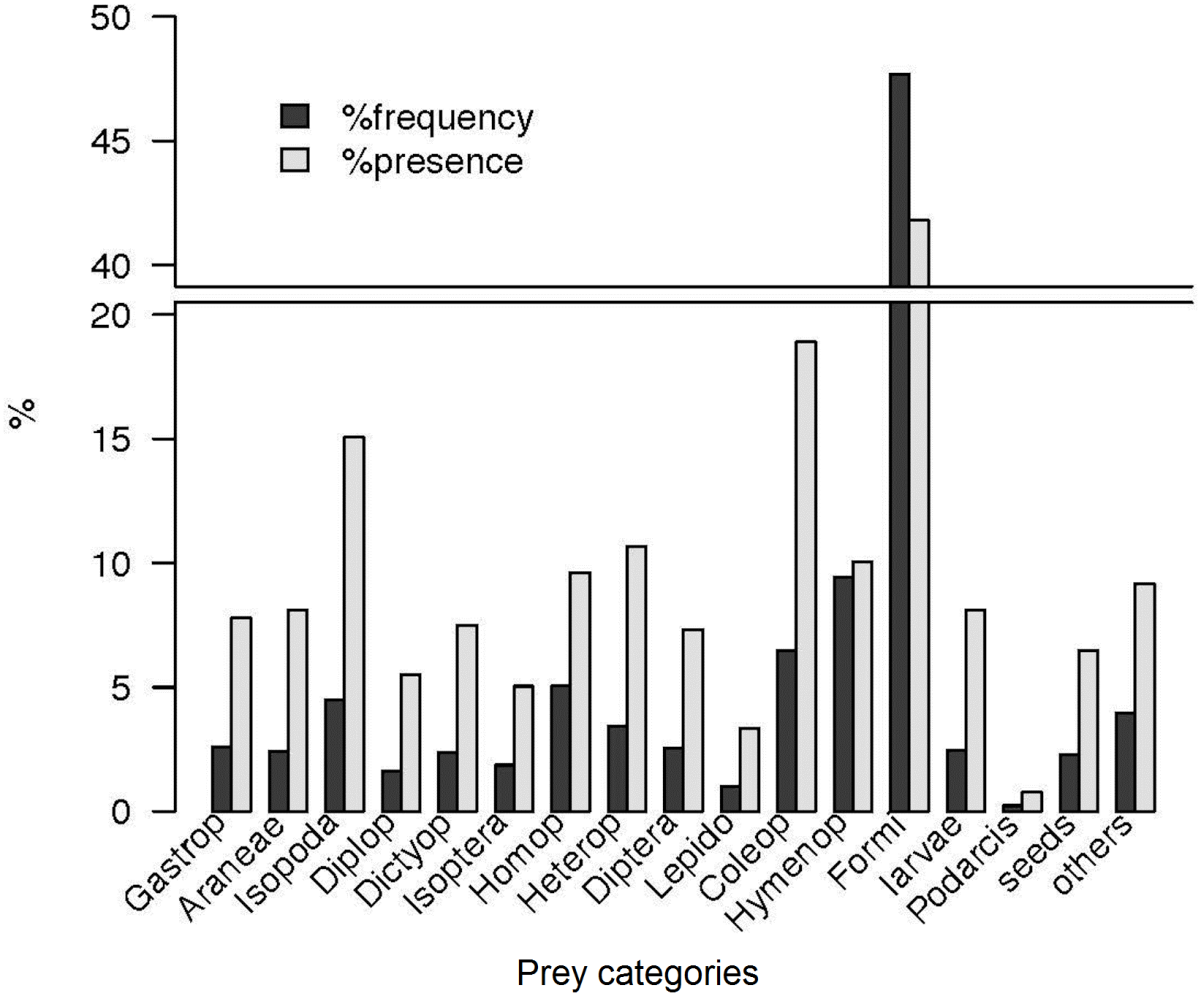
The overall diet in Aire Island by frequency. From the analysis of 7,790 faecal pellets of 17 years of study. %frequency represents prey abundance (%n) and %presence (%p) is the percentage of faeces containing a given prey type.

From the whole sample of 7,790 faecal pellets, we found 26,346 prey items belonging to 23 different prey categories. Ants were the most frequently found prey, followed by Coleoptera and terrestrial Isopoda (Fig 2).

**Fig 3:**
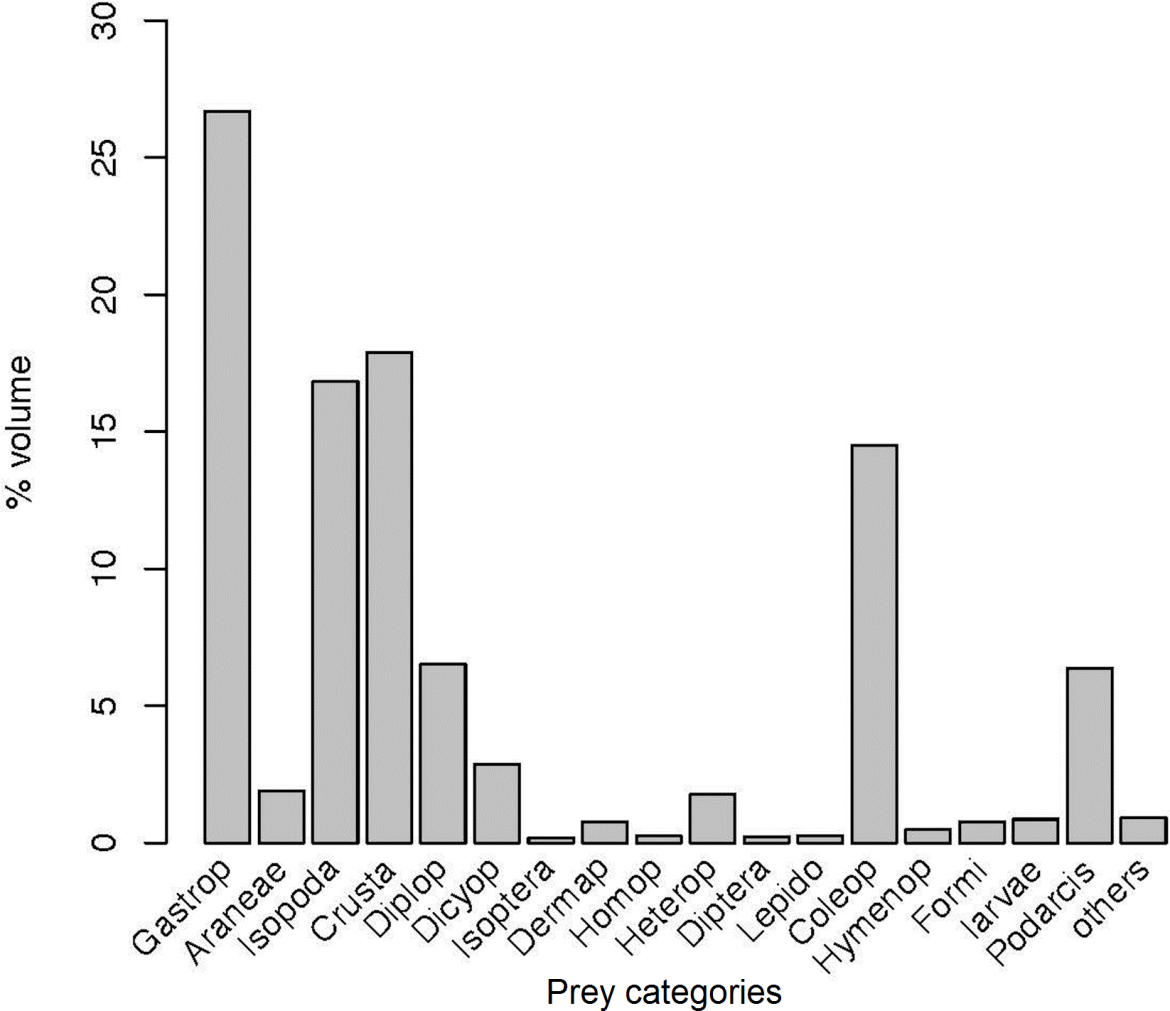
The overall diet in Aire Island by volume. Percentage contribution of each prey type by volume (see details in the text).

If the overall diet is represented by volume (Fig 3), the picture is completely different, with Gastropoda, Crustacea (including terrestrial Isopoda) and Coleoptera apparently being the main prey types. However, this representation could be misleading, because the very large volume assigned to snails' shells did not correspond to the real volume of the soft parts of the snail body effectively eaten by lizards. This problem was even worse in the case of other Crustacea different from Isopoda, where the volume was estimated from whole marbled rock crabs (see above) that in several cases were only partially consumed by lizards (pers. obs.). Regarding to Coleoptera, its high contribution in volume matches with its importance in terms of frequency (Fig 2). Thus, in this taxon, volume data may closely reflect its overall contribution to the diet.

### Annual diet variability

**Fig 4:**
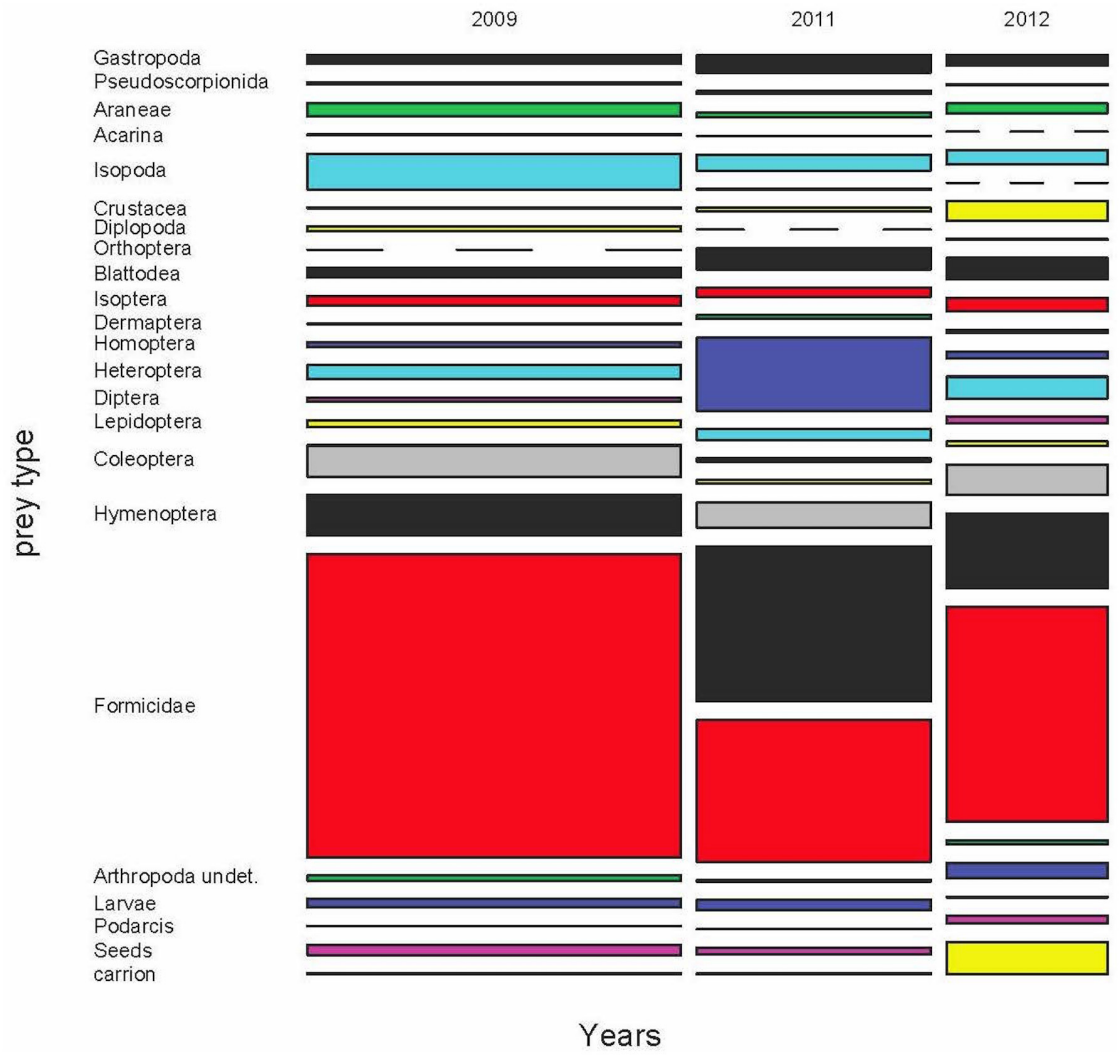
Mosaic plot of the variation of the diet of *Podarcis lilfordi* during 2009, 2011 and 2012 in Aire Island. The width of each annual column is proportional to the whole sample size for each year. The heights of the boxes within each column are proportional to the relative frequency of each prey type. Dashed lines indicate prey types to be absent in this annual sample. Same colour codes for each prey type were employed in all remaining figures of diet and prey availability (see details in the text).

**Fig 5:**
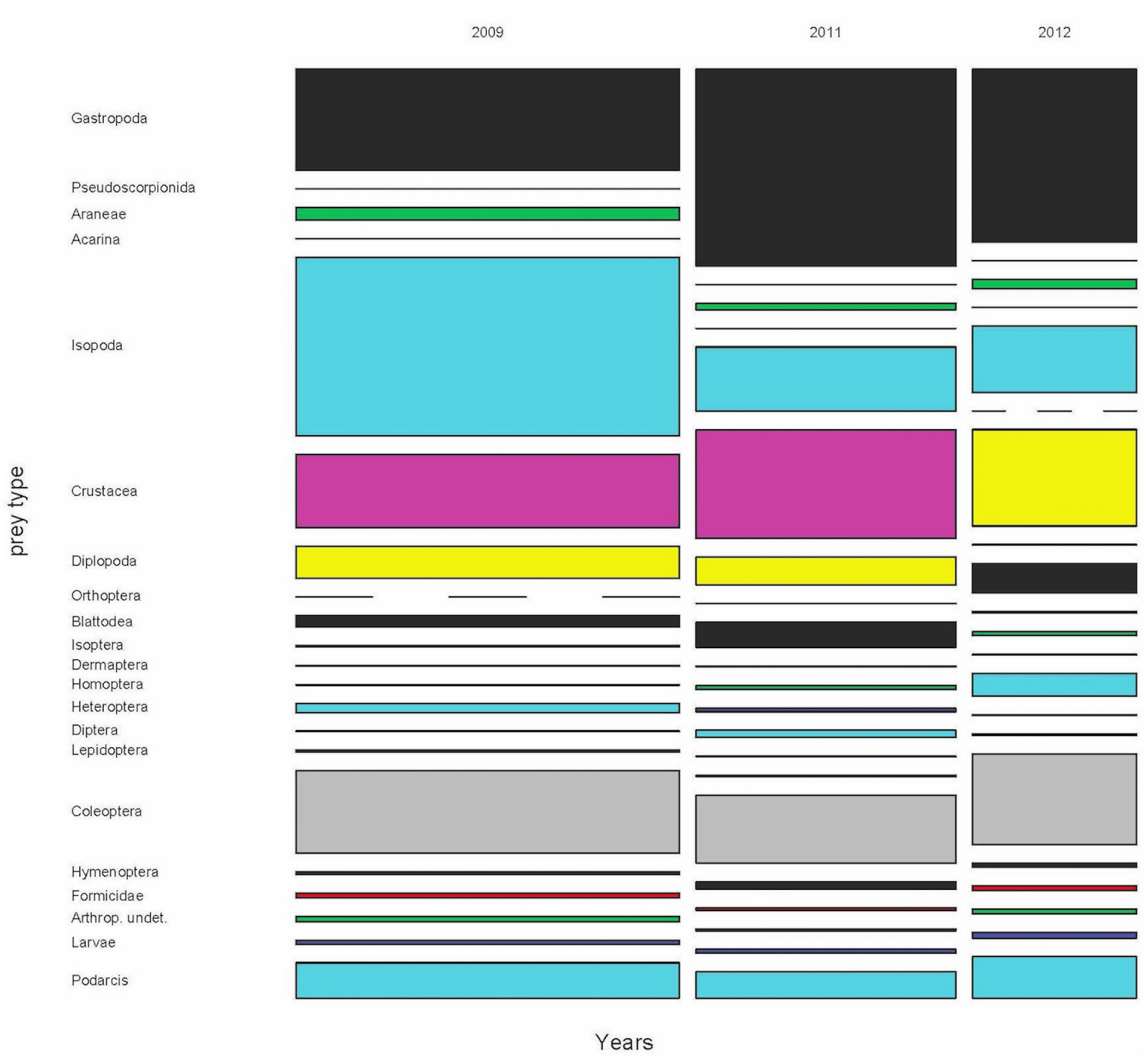
Annual variation of the diet of the Balearic lizard during 2009, 2011 and 2012 by volume. (see details in the text).

**Fig 6:**
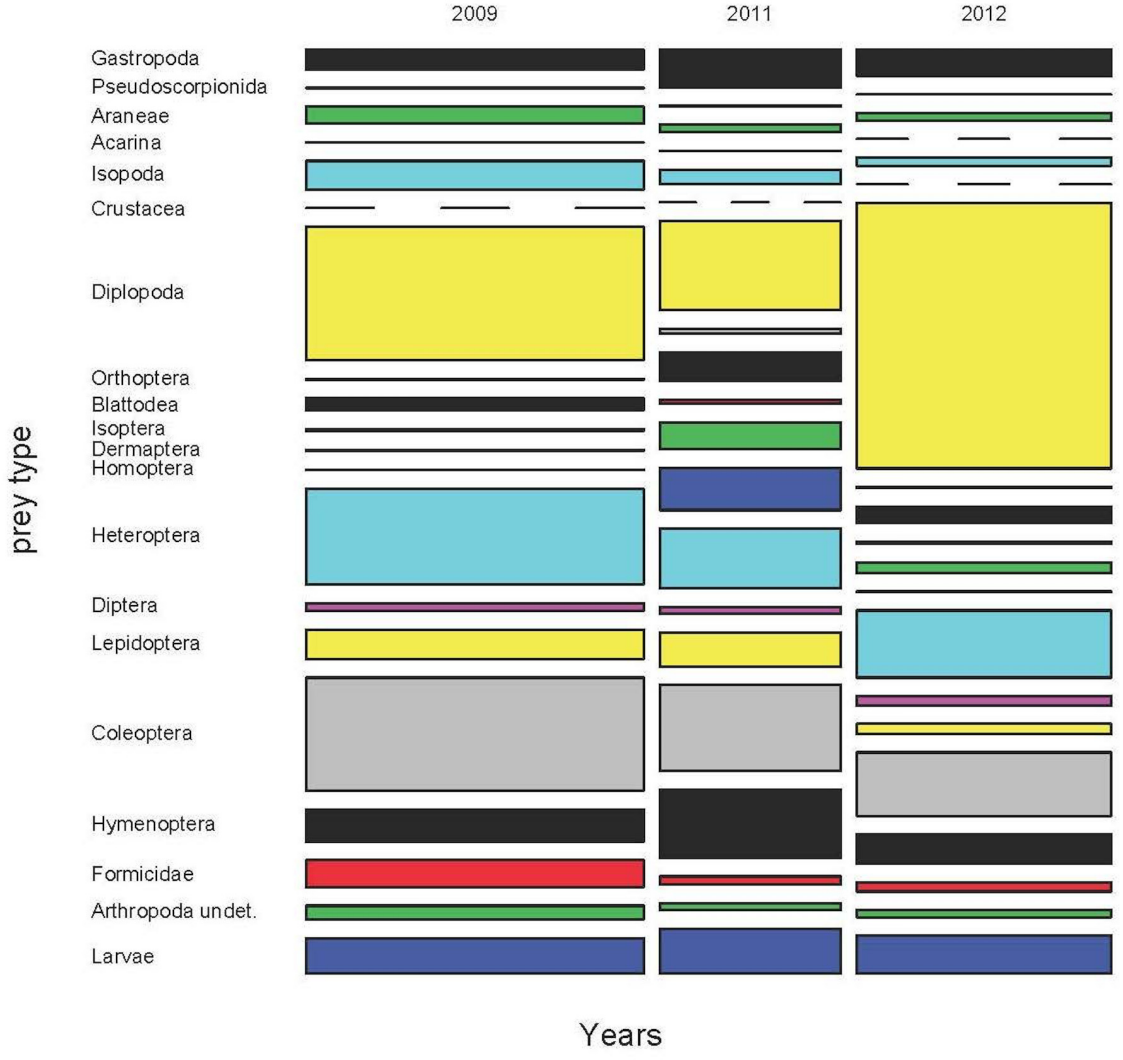
Annual variation of the diet of the Balearic lizard during 2009, 2011 and 2012 by biomass.

For years 2009, 2011 and 2012 we obtained a sample of 1870 scats from the months of May, June and July and from all areas of the island. Thus, we could compare the diets of these years (Fig 4 and S1–S3 Tables), taking into account both the month and the area of the island as potential sources of variation within the year. The results of the permutational MANOVA were clear. All factors (year, month and area), all paired interactions, and the triple interaction too, were statistically significant for prey frequencies, biomass and volumes (p<0.0001 in all cases, Figs 5 and 6).

The diet in the three years was mainly based in some clumped prey as Formicidae. Isopoda, Gastropoda and Coleoptera also contributed importantly, especially in terms of volume. Regarding biomass, the contribution of Diplopoda was essential, particularly during 2012 and 2009, whereas ants were almost negligible and other uncommon groups, as Heteroptera, had apparently a key role (Fig 6). However, this picture can be, again here, misleading. The presence (%p) of these groups was relatively low (S1–S3 Tables), indicating that the capture of a Diplopoda or Heteroptera was an infrequent event, excepting the case of Heteroptera in 2012 (S3 Table). Thus, the sporadic capture of a large prey item can be punctually important, but cannot be considered as a main energetic resource for the whole lizard population, as it would be if we only take into account the biomass results. The same reasoning could be applied to the sporadic presence of carrion or juvenile lizards in the diet (see below).

**Fig 7:**
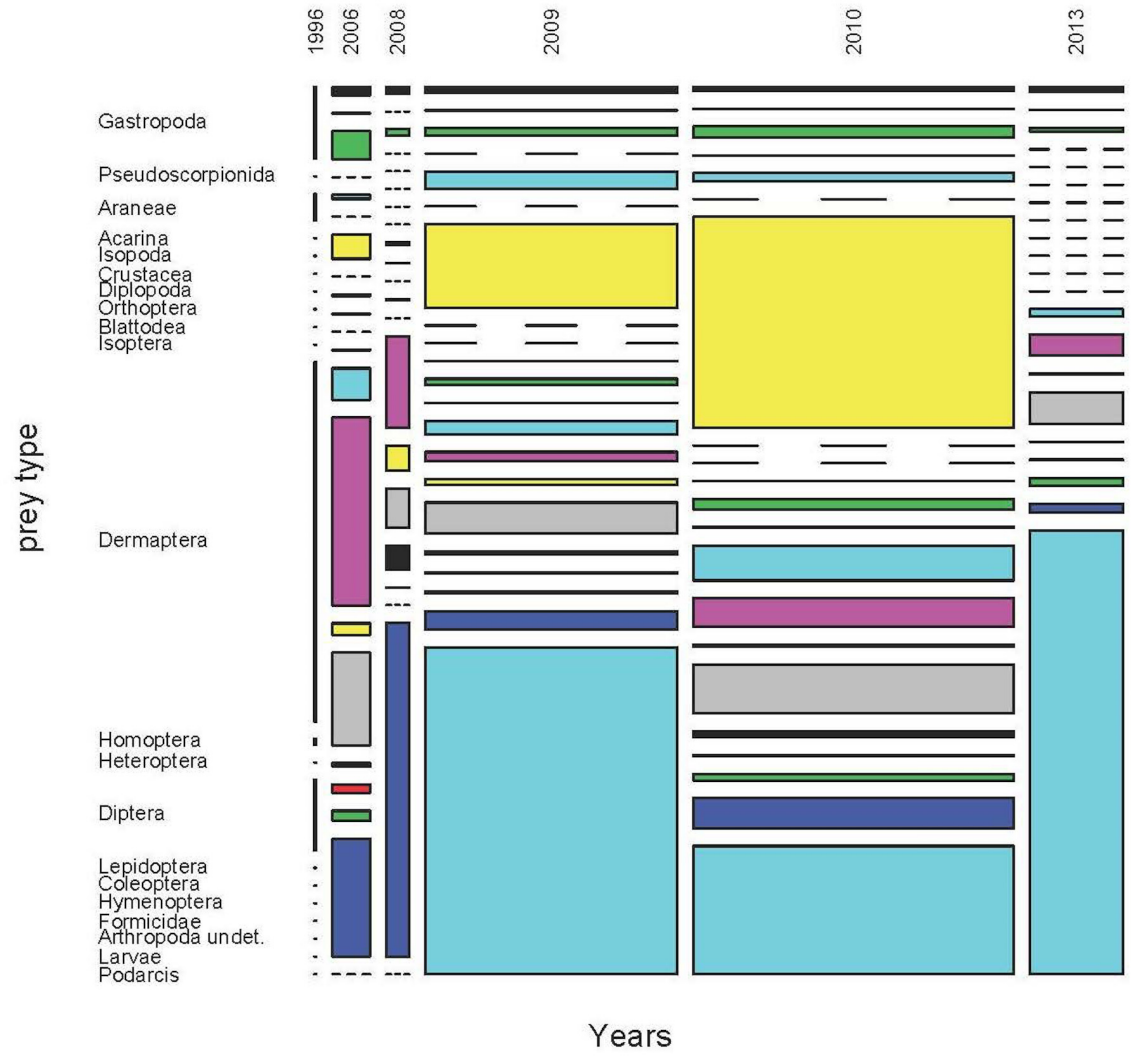
Annual variation of the diet of the Balearic lizard by biomass in High area of Aire Island. (See details in the text).

A clear example of the difficulties in the interpretation of biomass input corresponding to each prey type is showed in Fig 7, where the annual variation of the diet by biomass in the High area of Aire Island is depicted. In 2009 and 2013 the bulk of biomass seemed to correspond to a handful of juvenile lizards captured by their conspecifics. This would only be true if we suppose the entire consumption of the juvenile by the predator. Moreover, even if we discarded the inclusion of these biomass estimations of juvenile lizards, some secondary prey types by number or volume, as terrestrial Isopoda, appeared to be particularly important in 2009 and 2010. Again here, the correct interpretation of these results would need to include the percentage of presence of each prey type and a careful inspection of diets by frequencies and volumes.

If we compare dietary diversities among those three years: 2009, 2011 and 2012, we only found significant differences for all Hill numbers between 2009 and 2011 and (only for q = 0) between 2009 and 2012 (Tables 2 and 3).

**Table 2:** Diversity values from years 2009, 2011 and 2012.

Year	Simpson index
2009	0.6349 ± 7.29 x 10^−5^
2011	0.8051± 2.17 x 10^−5^
2012	0.7883± 7.49 x 10^−5^

Values of Simpson´s diversity index (± var).

**Table 3:** Pairwise comparisons of diversity values from years 2009, 2011 and 2012.

	Hill’s numbers		
Pairwise comparisons	q = 0	q = 1	q = 2
2009–2011	0.0000	0.0066	0.0458
2009–2012	0.0144	0.1618	0.2608
2011–2012	0.3016	0.3522	0.6138

Pairwise comparisons of diversities using three Hill's numbers (q = 0, q = 1 and q = 2, see more details in the text).

**Fig 8:**
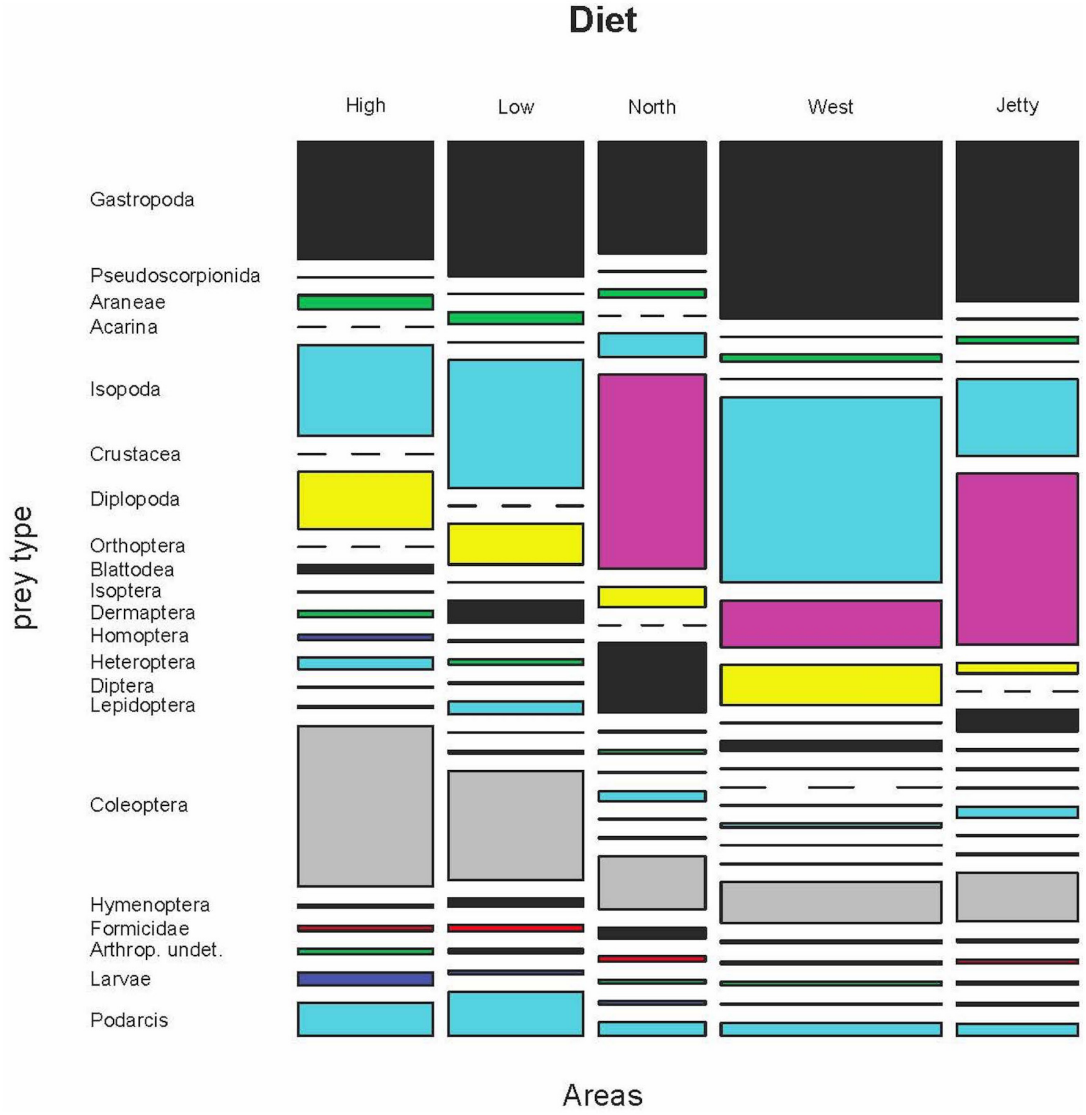
Variation of the diet by volume in five areas of Aire Island (years 2009, 2011 and 2012).

The comparison among different areas of Aire was also done with samples belonging to 2009, 2011 and 2012 (S4–S8 Tables). The results of the permutational MANOVA for prey frequencies showed that diet composition was significantly different among the five areas (F_4, 1752_ = 15.18, p<0.0001, Fig 8). Interestingly, trophic diversities were similar, without any differences in all pairwise comparisons (Tables 4 and 5).

**Table 4:** Diversity values in five areas of Aire Island.

Areas	Simpson index
High	0.7744 ± 7.30 x 10^−5^
Low	0.7085± 8.19 x 10^−5^
North	0.7198± 7.55 x 10^−5^
West	0.7531± 9.22 x 10^−5^
Jetty	0.7534± 1.56 x 10^−4^

Values of Simpson´s diversity index (± var).

**Table 5:** Pairwise comparisons of diversity values in five areas of Aire Island.

	Hill’s numbers		
Pairwise comparisons	q = 0	q = 1	q = 2
High-Low	0.1190	0.1585	0.1074
High-North	1.0000	0.9990	0.9990
High-West	1.0000	0.9962	0.9376
High-Jetty	0.3518	0.1060	0.0626
Low-North	0.3580	0.1420	0.1028
Low-West	0.1388	0.4962	0.6358
Low-Jetty	1.0000	1.0000	0.9990
North-West	1.0000	0.9582	0.8272
North-Jetty	0.3684	0.3430	0.4282
West-Jetty	0.3674	0.3430	0.4302

Pairwise comparisons of diversities using three Hill's numbers (q = 0, q = 1 and q = 2, see more details in the text).

**Fig 9:**
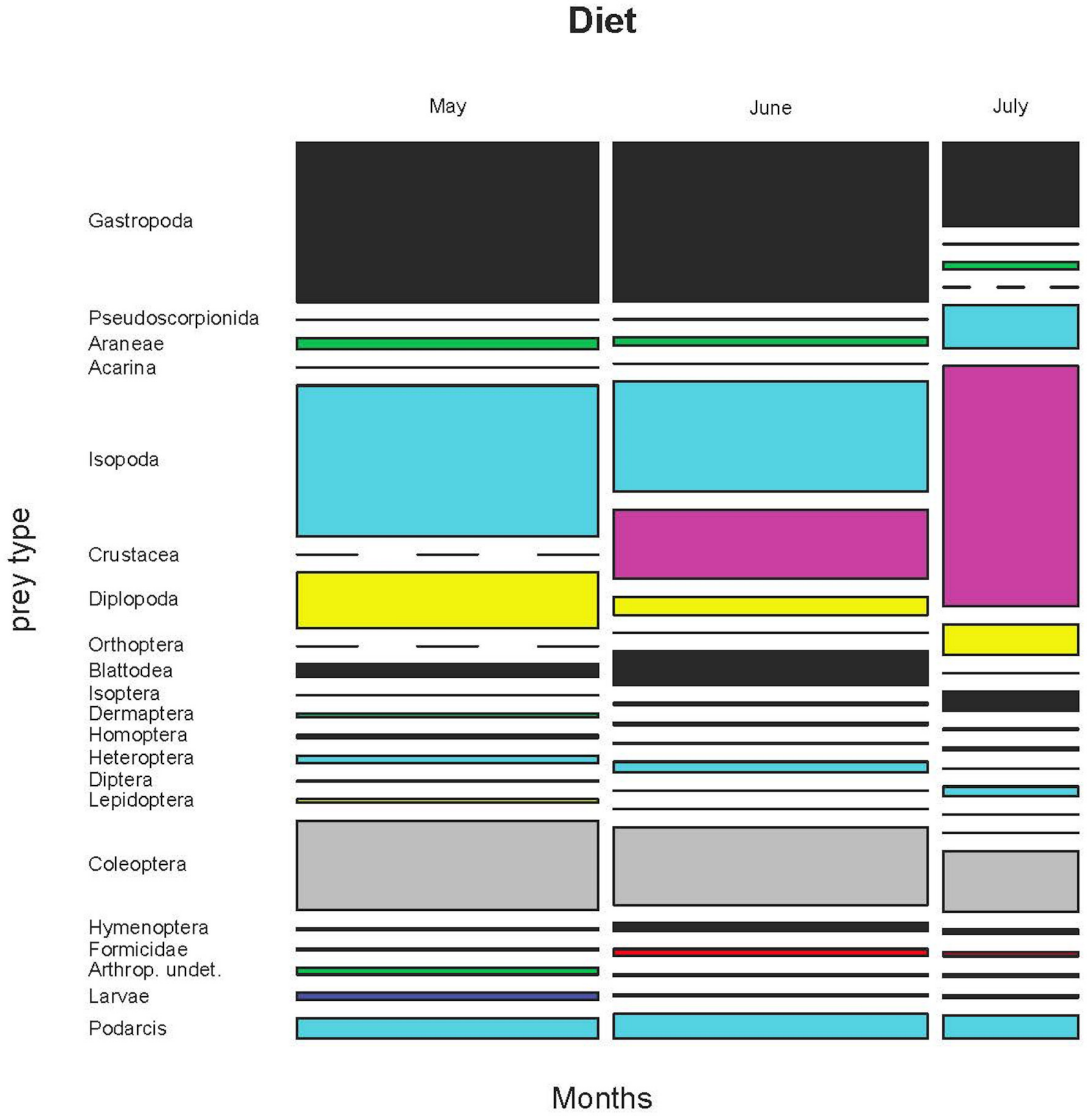
Variation of the diet by volume in three months (years 2009, 2011 and 2012).

The comparison of the three months of the same sample produced similar results in the case of permutational MANOVA (F_2, 1766_ = 18.16, p<0.001; Fig 9 for prey volumes, S6 Fig for prey frequencies and S7 Fig for biomass and S9–S11 Tables).

According to these results, the comparison of the same month along years was done only for those months with good interannual samples and for the same area of the island. Hence, we only analysed samples from the High area of Aire Island. In April, main prey types were ants, beetles, flies and flying hymenopterans (by volume see S1 Fig and S12–S17 Tables). Nevertheless, there were some remarkable differences among years, like the contribution of insect larvae in 2008 or Isopoda (blue squares) in 2009. These results led again to significant differences in the diet among years (permutational MANOVA, F_4, 816_ = 13.982, p<0.001 for prey frequencies and F_4, 811_ = 11.899, p<0.001 for volumes). The picture was similar in May, with significant differences among years (F_5, 504_ = 10.182, p<0.001 for prey frequencies and F_5, 481_ = 8.3385, p<0.001 for volumes), but with a higher contribution by volume of other prey items in some years, such as Homoptera in 2008 and 2011. This corresponded with a decrease in the consumption of ants (S2 Fig). In June, Coleoptera and Formicidae were again the main prey items by volume, with an important contribution of Diptera in 2008 and Gastropoda in 2011 (S3 Fig). There were also significant differences among years (F_5, 414_ = 9.628, p<0.001 for frequencies and F_5, 331_ = 8.4811, p<0.001 for volumes). Also in July we found significant differences among years (F_7, 310_ = 2.5613, p = 0.00099 for frequencies and F_7, 304_ = 2.1207, p<0.001 for volumes), with an even higher contribution of ants and beetles to the diet and the disappearance of several prey types that were present in previous months (S4 Fig). Finally, in August we found again significant differences among years (F_5, 264_ = 2.9965, p<0.001 for frequencies and F_5, 263_ = 3.5333, p<0.001 for volumes). In this summer month the diet was also dominated by ants and beetles. But some prey types raised its volume, such as juvenile lizards (but see below) or Homoptera (S5 Fig).

**Fig 10:**
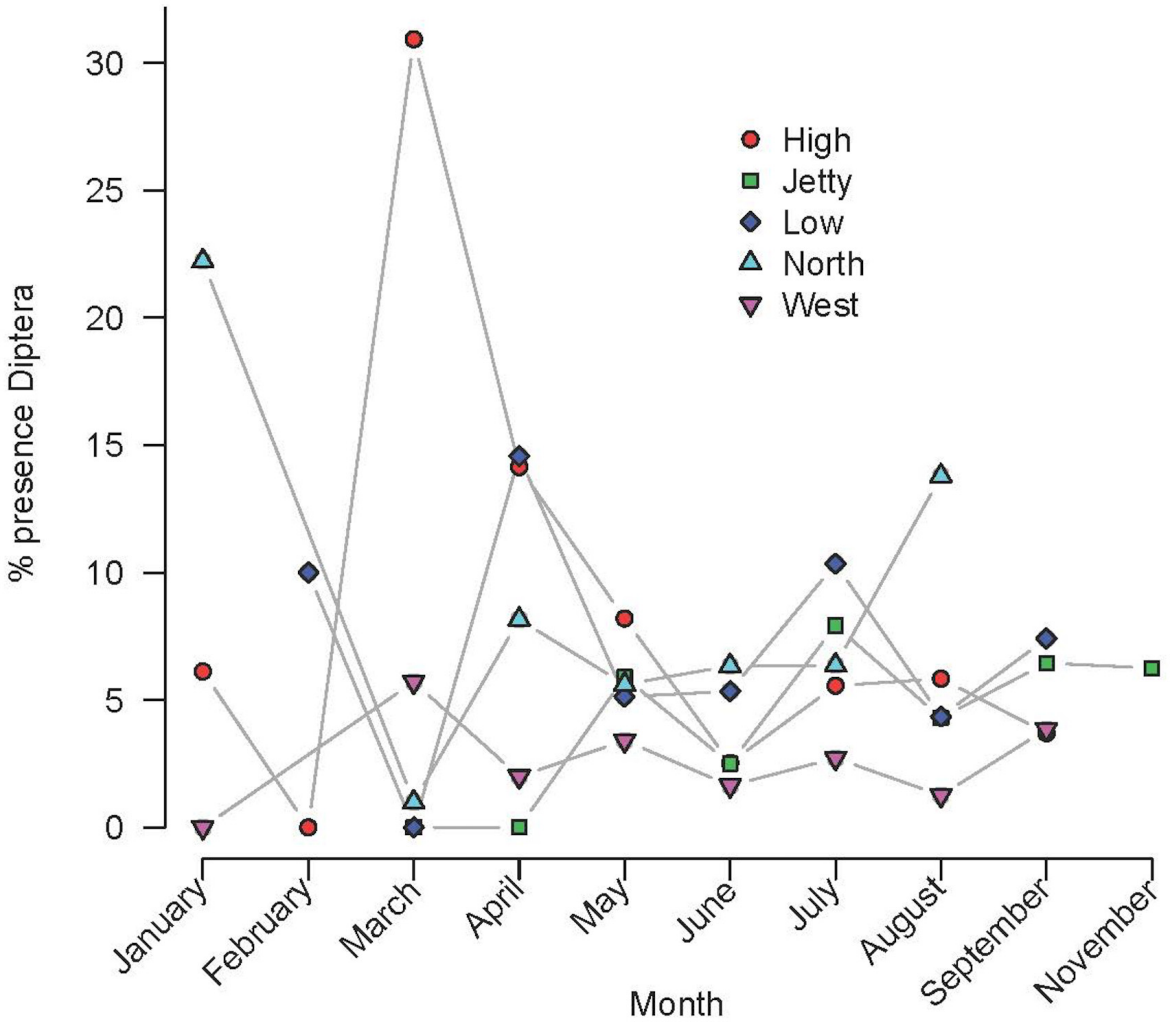
Monthly variation in the capture of Diptera at different areas of Aire Island. (see the presence of Diptera in High area during March).

**Fig 11:**
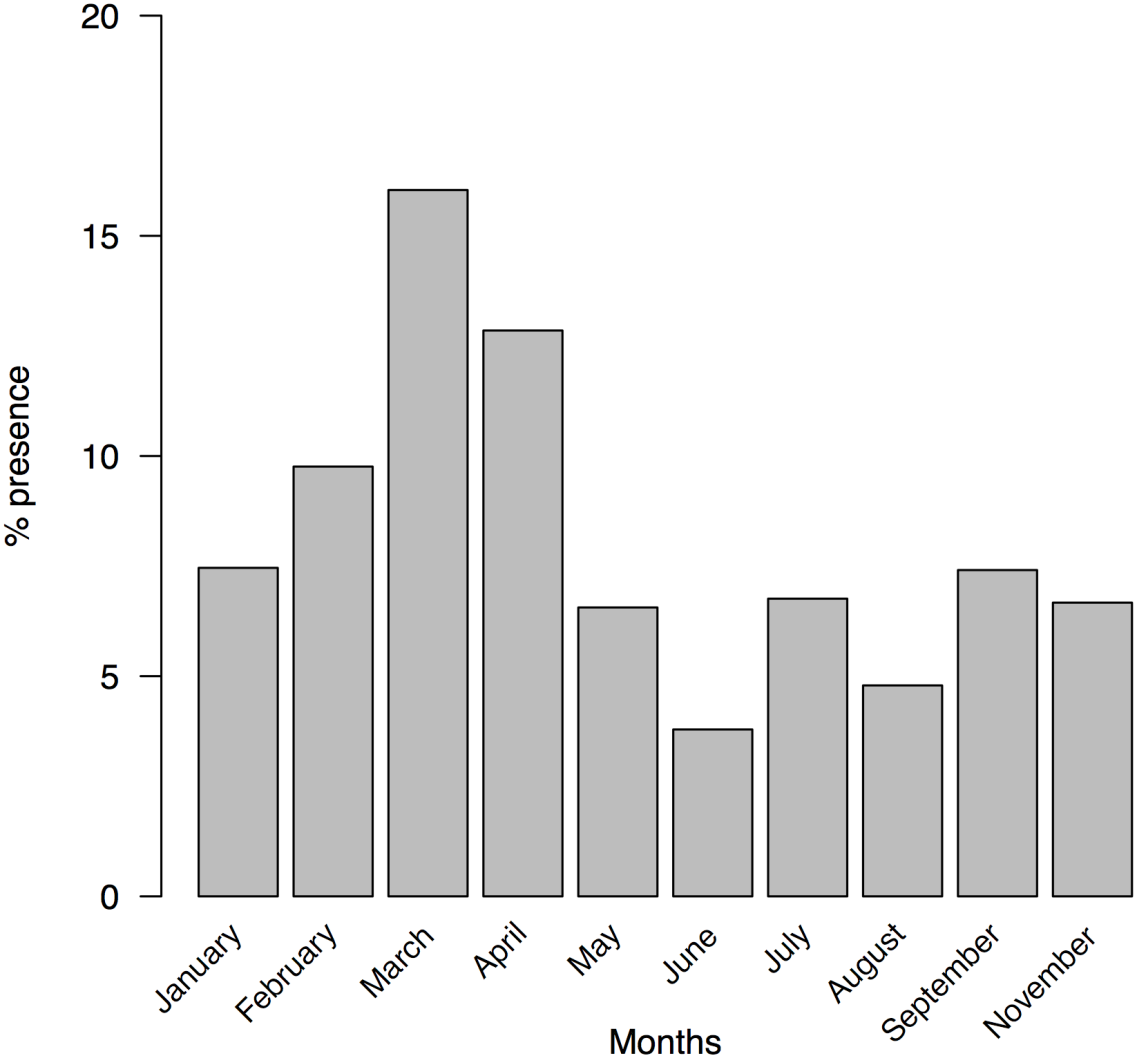
The presence of Diptera in the diet of *P*. *lilfordi* along the months. (samples from all studied areas were pooled, see more details in the text).

Factors driving the consumption of a given prey type are clearly difficult to discern. But in some cases we can infer some clues. Flies (Diptera) were captured along all months under study and in all areas of the island (Fig 10), but there were highly significant differences among months (G-test, G = 125.24, d.f. = 9, p = 2.2x10^-16^). Pairwise comparisons indicate that these differences were mainly due to a higher consumption of flies during March and April (Table 6 and Fig 11). In addition, flies were mainly captured in High and Low areas. In fact, during March, more than 30% of faeces from High area contained flies (Fig 10).

**Table 6:** Pairwise comparisons with G-test of Diptera consumption among months under study (see also Fig 10).

	February	March	April	May	June	July	August	September	November
**January**	0.905	0.184	0.410	0.917	0.410	0.928	0.630	0.989	0.980
**February**		0.513	0.815	0.715	0.260	0.741	0.430	0.881	0.915
**March**			0.430	**7.4e-05**	**2.2e-09**	**0.00011**	**1.6e-06**	**0.040**	0.513
**April**				**2.4e-08**	**< 2e-16**	**6.8e-08**	**1.0e-09**	0.124	0.715
**May**					**0.0013**	0.928	0.213	0.905	0.989
**June**						**0.0006**	0.465	0.148	0.869
**July**							0.159	0.917	0.989
**August**								0.428	0.917
**September**									0.980

Significant results are shown in bold.

### Availability and consumption

**Fig 12:**
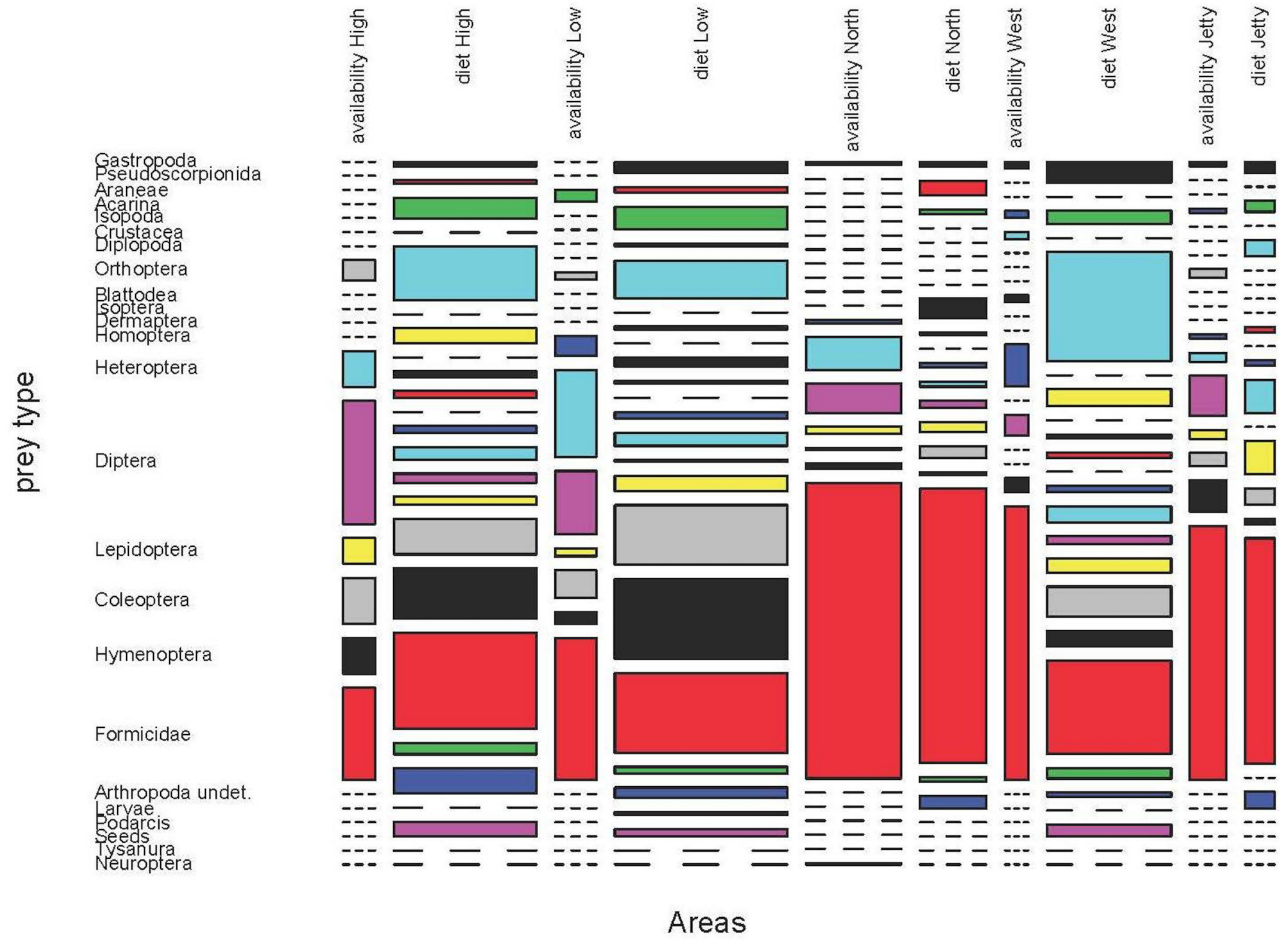
Prey availability and diet of *P*. *lilfordi* in May 2009 at five areas of Aire Island. We showed all prey types, including those obviously absent in the availability samples (as seeds or juvenile *Podarcis*) or diet, to enable comparisons.

**Fig 13:**
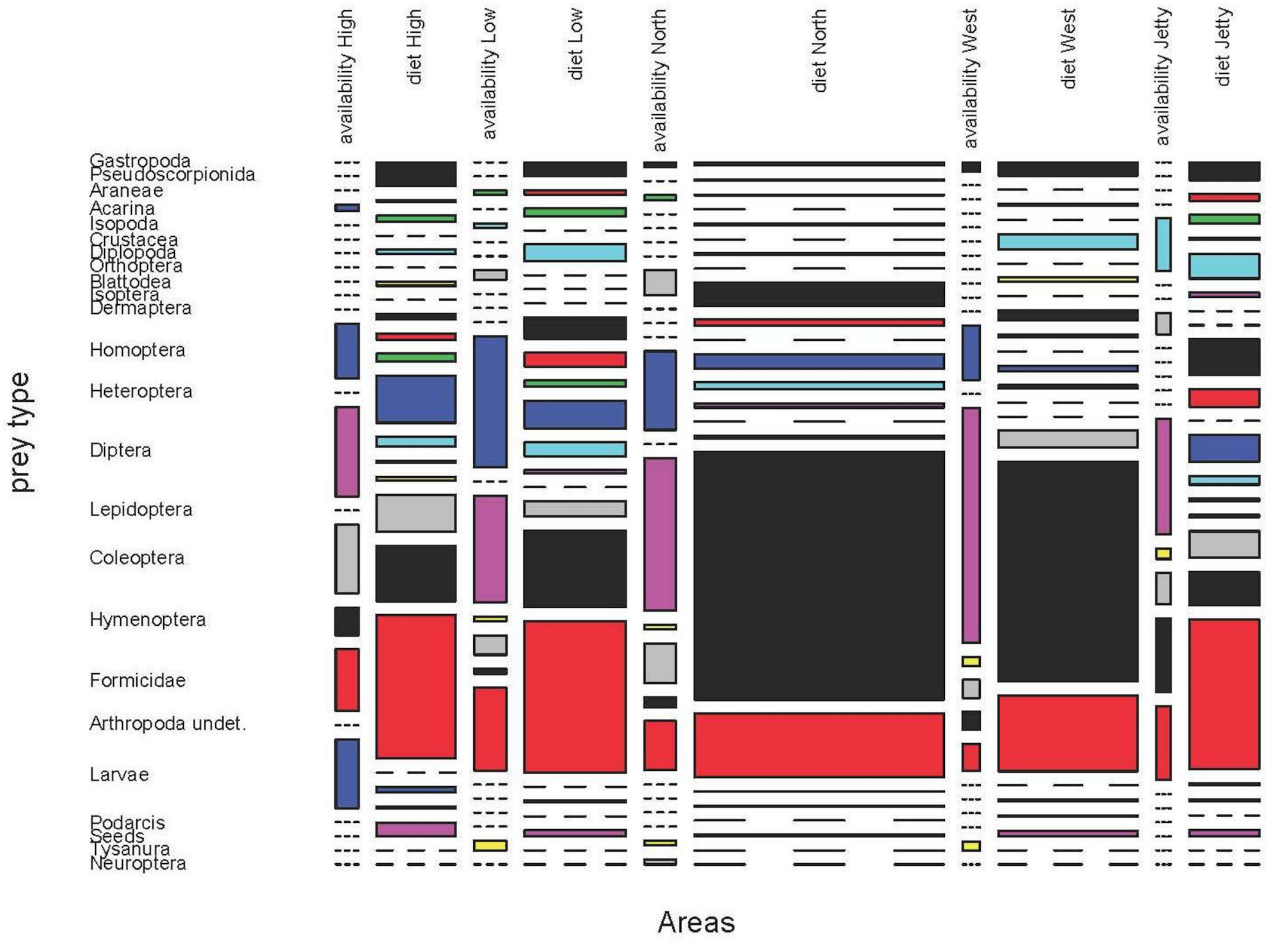
Prey availability and diet of *P*. *lilfordi* in June 2011 at five areas of Aire Island.

With the aid of biocenometers, we obtained 531 invertebrates in May 2009 and 288 in June 2011. An important source of variation in the diet would be different prey availabilities. Even within a small islet as Aire, trophic availabilities can be surprisingly different among years, months or areas (Figs 12 and 13). During May 2009 and in the five areas under study, the most common epigeal or flying prey items were Formicidae, Coleoptera, Diptera, Hymenoptera and Heteroptera. But all of these prey types had a very variable contribution to the whole availability of each area (Fig 12 and S44–S48 Tables). A similar pattern was observed in June 2011 (Fig 13 and S49–S53 Tables), with a higher contribution of Hymenoptera and Homoptera in some areas (North and West) but a lower presence of Heteroptera. In North, West, High and Low areas of Aire larger values of diversity were shown (Table 7), while the lowest value was detected in North area.

**Table 7:** Diversity values from five areas of Aire Island for diet and availability.

	High	Low	North	West	Jetty
**Diet**	0.88 ± 8.91 x 10^−5^	0.87 ± 6.26 x 10^−5^	0.47 ± 2.49 x 10^−3^	0.84 ± 1.81 x 10^−4^	0.63 ± 4.09 x 10^−3^
**Availability**	0.81 ± 6.27 x 10^−4^	0.77 ± 7.10 x 10^−4^	0.38 ± 1.60 x 10^−3^	0.47 ± 6.49 x 10^−3^	0.53 ± 3.86 x 10^−3^

Values of Simpson’s diversity index (± var).

In all cases, we observed a significant difference in prey composition between availability and consumption (Fisher exact test, p<0.001 in all pairwise comparisons, Tables 8 and 9). Moreover, several prey types captured by lizards were absent in the availability samples (S44–S53 Tables). This fact would confirm that samples from biocenometers can only effectively represent the availability of epigeal and flying prey. The most important prey item by frequency, Formicidae, showed variable values of electivity, with low positive or negative electivities in May 2009 and positive or slightly negative values in June 2011.

**Table 8:** Pairwise comparisons of Simpson’s diversity index.

		High		Low		North		West	
Areas	Hill’s numbers	Availability	Diet	Availability	Diet	Availability	Diet	Availability	Diet
Low	q = 0	<0.001	<0.001						
	q = 1	<0.001	<0.001						
	q = 2	<0.001	<0.001						
North	q = 0	1.00	1.00	<0.001	<0.001				
	q = 1	1.00	1.00	<0.001	<0.001				
	q = 2	1.00	1.00	<0.001	<0.001				
West	q = 0	<0.001	<0.001	0.0158	0.8274	<0.001	0.0002		
	q = 1	<0.001	<0.001	0.0720	0.7636	<0.001	0.0002		
	q = 2	<0.001	<0.001	0.1324	0.7060	<0.001	0.0004		
Jetty	q = 0	<0.001	<0.001	<0.001	<0.001	<0.001	1.00	0.2044	<0.001
	q = 1	<0.001	<0.001	<0.001	<0.001	<0.001	1.00	0.4574	0.0002
	q = 2	<0.001	<0.001	<0.001	<0.001	<0.001	1.00	0.5330	0.0002

Pairwise comparisons using three Hill's numbers (q = 0, q = 1 and q = 2). In each cell we give the three p-values of the three Hill's numbers compared for availability and for diets.

**Table 9:** Comparisons of diversities between availability and diet for each area of Aire Island.

	Areas				
Hill’s numbers	High	Low	North	West	Jetty
q = 0	1.00	0.9268	<0.001	<0.001	<0.001
q = 1	1.00	0.9474	<0.001	<0.001	<0.001
q = 2	1.00	0.9716	<0.001	<0.001	<0.001

Thus, the high consumption of ants was, at least partially, a reflection of its abundance. The abundance of ants even exceeded its consumption during spring (May 2009). With regard to Coleoptera, which were the most important prey item by volume (see above), the situation was radically different in both periods of study. In May 2009, lizards positively selected beetles, while in June 2011 they seemed to be partially avoided. In the case of flying prey the situation was also rather variable. Diptera were relatively common in both years and all areas. However, lizards showed negative values of electivity for flies, although these were intensively consumed during March and April (see above). A remarkable situation was observed in the case of Hymenoptera, which were positively selected in some areas while avoided in others (S44–S53 Tables).

### Cannibalism and carrion consumption

**Fig 14:**
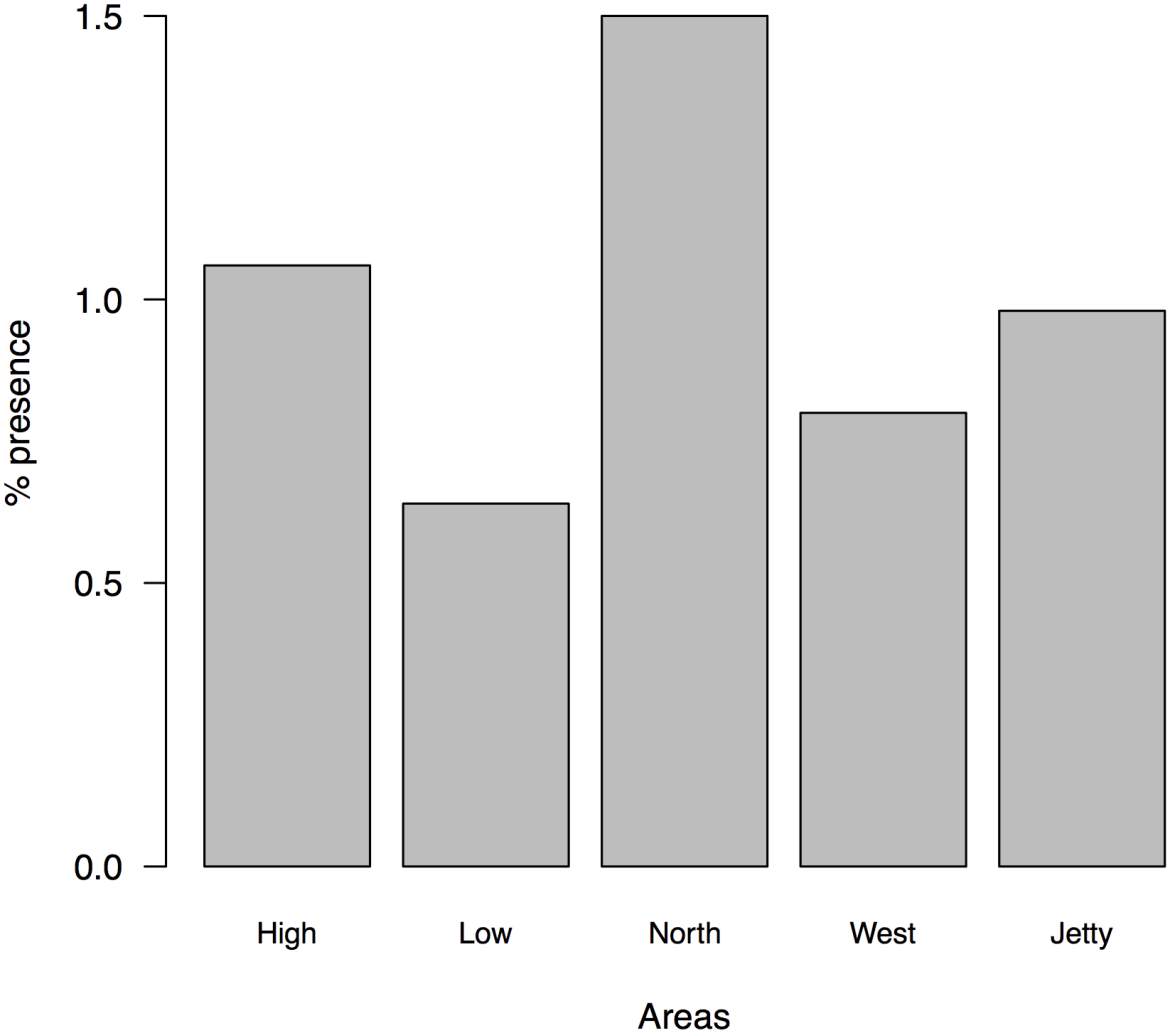
The consumption of *P*. *lilfordi* as a prey item in different areas of Aire Island. (see details in the text).

In the whole dataset we found 61 remains of *P*. *lilfordi*, corresponding to 12 different years. Comparing frequencies of consumed lizards in relation to prey frequencies for each available month (we do not have data from November and December), we found significant differences among months (Fisher exact test, p = 0.00050), with the highest consumption in August (24 individuals), accounting for almost 40% of all captured lizards (Fig 14).

**Fig 15:**
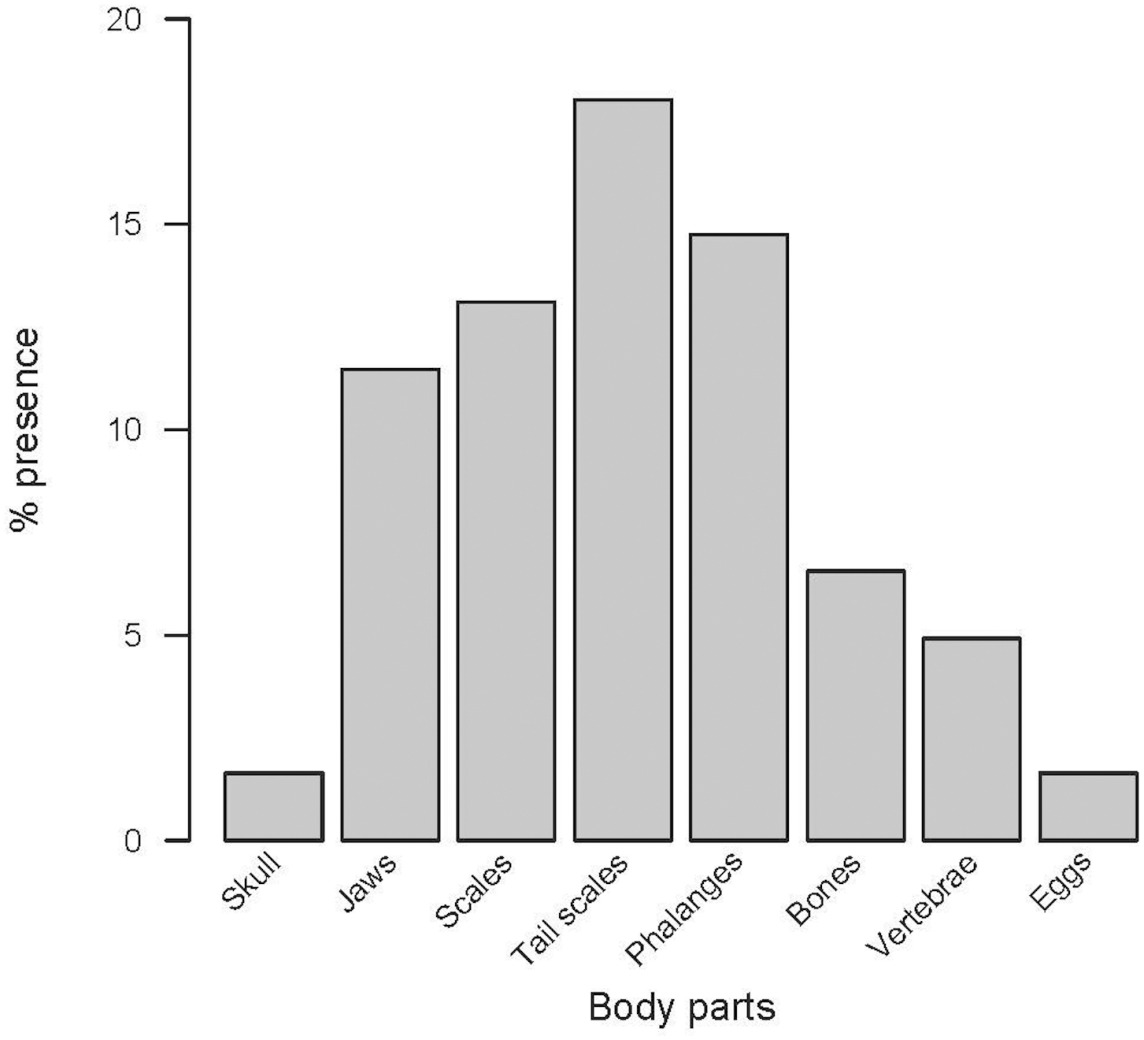
Identified remains of *P*. *lilfordi* found in faecal samples. (see details in the text).

The remains of lizards identified in faeces mainly consisted in tails, toes or skin (Fig 15), while less than 15% of remains were identified as head or body parts. Thus, we cannot be sure that all these captures involved the death of lizards and their whole consumption.

Carrion remains from dead mammals or birds were observed in 107 faecal pellets. There was a significantly higher proportion of pellets containing carrion remains in Jetty and West areas (G-test, G = 40.87, d.f. = 4, p = 2.86 x 10^−8^, see pairwise comparisons in Table 10).

**Table 10:** Pairwise comparisons of G test of frequencies of carrion consumption in the five areas of Aire Island.

Areas	High	Low	North	West
**Low**	0.28			
**North**	0.12	0.03		
**West**	0.001	0.03	0.0001	
**Jetty**	7.4 x 10^−6^	0.0007	2.8 x 10^−6^	0.28

### Plant consumption

**Fig 16:**
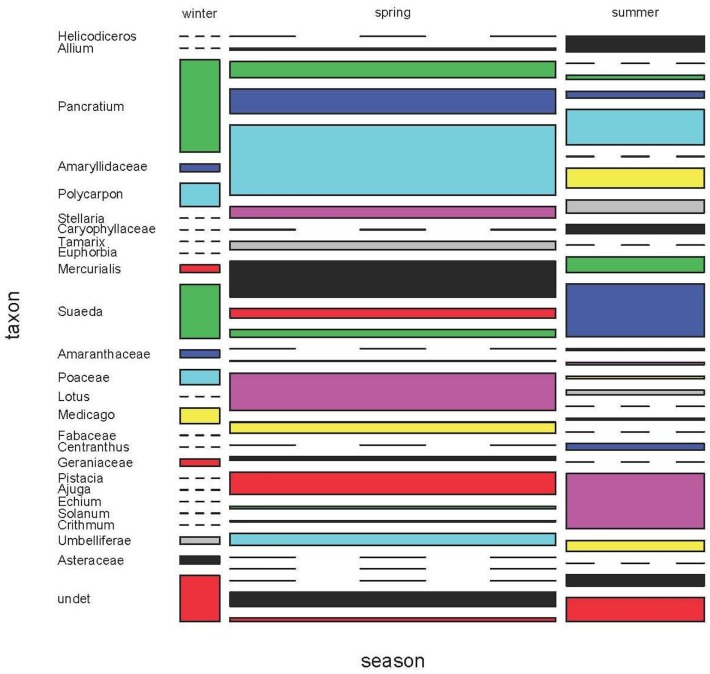
Plant consumption in Aire Island during three seasons of 1996. Taxa are showed as genera and families.

We found vegetal matter in 43.92% of faecal pellets, although its consumption some months can be even higher and thus, extremely important (Fig 16). When we analyzed the subset of 1996, we found that the percentage of plant matter was slightly different in the three seasons (Kruskal-Wallis test, χ^2^ = 6.0717, d.f. = 2, p = 0.048), but this small dissimilarity did not result in significant differences between seasons (all pairwise comparisons, p>0.05). However, the taxonomic composition of vegetal items found in diet was significantly different among the three seasons (permutational MANOVA, F_2,169_ = 20.047, p = 0.0009), with significant differences in all pairwise comparisons (Fisher test, p<0.001). Finally, diversity values of vegetal items were similar in the three seasons (Simpson's diversity, winter: 0.85 ± 0.001, spring: 0.890 ± 0.00008 and summer: 0.899 ± 0.0001; in all pairwise comparisons of the three Hill's numbers, p>0.05).

**Fig 17:**
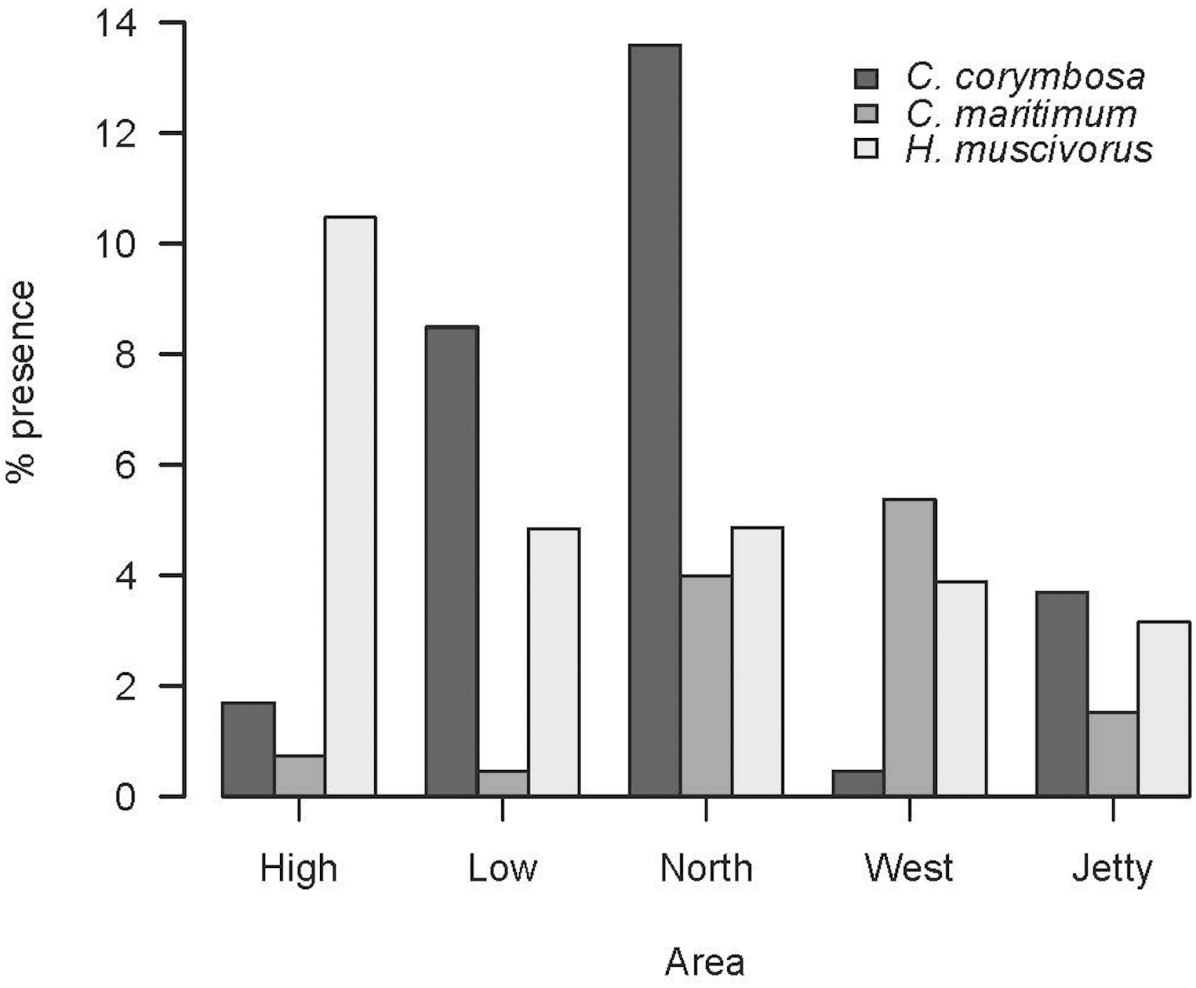
The consumption of three plant species in the five areas of Aire Island.

Regarding those plans identified up to species level, there were clear differences in its consumption among the studied areas. In the case of the sea fern, *Crithmum maritimum*, it was significantly different among the five areas of Aire (G-test, G = 95.19, p = 2.2 x 10^−16^, Fig 17). In pairwise comparisons we observed a similar consumption in High and Low areas (p = 0.2603), both with lowest values (Fig 17) and in North and West peninsulae (p = 0.2218), both with highest values.

Lizards ate fruits from different plant species during all months under study, excepting February, showing a higher consumption during May, June and July. Furthermore, 80.63% of all fruits detected during the study belonged to the dead horse arum, *Helicodiceros muscivorus*. Fruits of the dead horse arum accounted for 5.99% of the whole prey items and 22.39% of prey recorded in June. When comparing months, fruits were more consumed during June (G test, G = 645.45, p = 2.2x10^-16^), with significant differences among all months under study (pairwise G test, p<0.001) excepting between April and August (p = 0.050). As in the case of the sea fern, the consumption of dead horse arum fruits was not uniform throughout Aire Island (G-test, G_4_ = 76.277, p = 1.07 x 10^−15^). In fact, it was significantly higher in High area (Table 11). Apparently, the presence of *H*. *muscivorus* in Aire would be relatively recent, as we did not detect fruits of the dead horse arum in the first year of sampling (1995).

**Table 11:** Pairwise comparisons with the G test of the consumption of fruits of *H*. *muscivorus* among the five areas of Aire Island.

Areas	High	Low	North	West
**Low**	**1.1 x 10**^**−8**^			
**North**	**3.3 x 10**^**−7**^	0.74		
**West**	**2.0 x 10**^**−7**^	0.61	0.82	
**Jetty**	**3.4 x 10**^**−9**^	0.25	0.54	0.61

Frugivory was significantly higher only in high area (bold values, see Fig 17).

## Discussion

The majority of dietary studies conducted on lizards has been based on a limited sample size and comprised a short period of time. Nevertheless, annual or seasonal variations in diet can occur and were indicated in some long term studies [53, 54]. These variations, as well as the detection of some prey consumed at very low rates, require the study of large sample sizes spanning several months and years [15]. As well, the simultaneous interpretation of results in terms of frequency, presence, volume and/or biomass is necessary to better understand which prey categories are important for the whole predator population or which ones are punctually important just for some individuals.

It was extremely difficult to interpret the diet of the Balearic lizard in terms of availability, energy demands or environmental constraints. We showed that the diet was awfully variable each year and, within each year, among months or among different areas of the island. Almost in all annual, monthly or local samples we can see that some taxa are always represented. It is the case of ants, an omnipresent prey item, with only a low contribution in biomass, but an important contribution by volume. Hymenoptera, Coleoptera, Homoptera and Isopoda were usually the next prey categories in importance of frequency or presence. However, several of those differences relied on some of the remaining prey types, which showed an extreme variability in their presence. For example, some prey, as Diplopoda, can be very important in some areas and years, but would be considered as globally irrelevant in the general diet of the Balearic lizard.

Also, the consumption of some prey types can be interpreted as the opportunistic exploitation of a resource linked with the peak of blooming of a particular plant species. It seems to be the case of Diptera consumption. We showed that flies were captured during the whole year, but especially during March and April, coinciding with the blooming of the dead horse arum, *H*. *muscivorus* in Aire Island [55]. Bow flies are attracted by this plant species for pollination and lizards employ blooming plants as thermoregulatory and foraging perching sites [55]. Hence, in this particular case, population dynamics of a plant species together with the existence of a very particular interaction with lizards, allowed an intense consumption of an uncommon prey type during the rest of the year. This interpretation is strengthened by observing the very high consumption of flies in the High area of Aire, where we recorded the highest densities of *H*. *muscivorus* (Fig 11; [55]).

Particularities of island environments have a fundamental effect on the diet of animals, as well as in their behaviour, physiology and morphology [9, 12, 56, 57]. To overcome the variability and unpredictability of food resources, island populations can expand their feeding preferences and/or maximize energy acquisition [4, 11]. Thus, omnivory is widespread in islet habitats, as specializing in the consumption of a narrow range of food resources could give rise to dramatic consequences during the year or the season when those resources became less abundant [58].

Also, the consumption of ants is extremely common in Mediterranean islands, probably due to the scarcity of more profitable prey in this kind of arid environments [3,59–61]. In order to interpret the acute variability of prey consumption along the time and space, we need to take into account that lizards do not permanently follow the rule of consumption of the most profitable prey. When the habitat is poor in terms of availability, predators can then adopt a variable strategy, capturing less profitable prey, as ants or any other clumped prey items. In these poor environments, the long-term maximisation rate would be more realistic as a foraging strategy [62]. Moreover, functional models of diets can only define optimal solutions to particular problems, but they do not take into account the role of feedback in foraging behaviour. Learning provides the feedback that animals need for a rapid adaptation to an ever-changing environment [63]. It is the case of Aire Island, where environmental conditions are extremely variable among different years, months or even areas. In this scenario, we should expect quick learning processes to use novel food resources, as well as learning from conspecifics [64]. The exploitation of these new food resources appears to spread very rapidly among the lizard population, indicating that some kind of social learning is working among conspecifics. Thus, social enhancement could play an important role in the successful survival in this poor and unpredictable environment [64].

As truly active foragers, Balearic lizards are able to capture an extremely wide variety of prey items, from aggregate prey as ants or some Homoptera, to hidden and slow-moving prey as terrestrial Isopoda, millipedes or insect larvae. It is the case of the Heteroptera *Brachypelta aterrima* (= *Cydnus aterrimus*). This species is present within root system of shrubs or under stones and basically has a nocturnal activity [65]. Thus, we can consider this insect as a fossorial prey. Since it is an insect species mainly present in sandy areas, we could expect a higher presence in North area of the Aire Island, where soft substrates are common. This case illustrates that the Balearic lizard has a very active foraging behaviour, searching for prey not only under stones and within crevices, but also in the base of shrubs and under sand substrates. These prey types are consequently underrepresented or even absent in availability samples. The estimation of trophic availability made with the biocenometer can give a good representation of epigeal and flying invertebrates [18], available to an active forager as *P*. *lilfordi*, but not of those prey captured under stones or inside rock crevices, both unreachable sites to sample. Hence, the values of electivity indices should be taken with caution. Maragou et al. [66] recognized a similar problem in the comparison between the diet of lizards and availability estimated with pitfall traps. From our viewpoint, no sampling device can reliably imitate the complexity of foraging behaviour of lizards and, consequently, there will always be a clear bias in the estimation of prey truly available to lizards. That seems to be particularly right in the case of this extremely active and omnivorous lizard living in Aire.

Regarding this relation between prey availability and diet, in the case of Sanitja Islet (Menorca, Balearic Islands), no matching was found between ranked available prey sizes and prey sizes in the diet, which indicated a lack of selection for larger prey items [11]. In Aire Island, our previous data indicated that there was a negative correlation between available and consumed prey sizes. Thus, in Aire Island there was a selection of the larger and less abundant prey [11]. These findings were only partially confirmed in this study, where abundant ants remaining as one of the most important prey item.

Lizards are clearly able to feed from several unexpected food resources, as carcasses from death gulls or mammals. In addition to the capture of flying insects attracted by carcasses, lizards also directly eat the meat. Carrion was mainly consumed in Jetty area, where rabbits and gulls are attracted by human leftovers (pers. obs.) or in West area, where the majority of gulls breed (pers. obs.). To obtain convenient pieces of food, lizards employ a spinning maneuver to subdue and dismember large pieces. A maneuver referred to as the 'death roll', previously described in Crocodilians [67]. This behaviour is also useful to pull pieces of inflorescences away from some plant species, as we described in the case of the thistle *Carlina corymbosa* [14].

Low levels of cannibalism are characteristic of generalist predators from many taxonomically diverse groups, including reptiles [68]. This behaviour has been reported in some insular lizards, such as *Gallotia caesaris* [15], *Podarcis gaigeae* [69] or *Podarcis atrata* [70]. In Aire Island, as well as in other species, the highest consumption of conspecifics coincided with the peak of hatchlings [71]. In any case, the remains of lizards found in scats indicated that, probably, an unknown proportion of juveniles were not totally consumed, but only their more vulnerable body parts, like tail fragments. Apparently, the consumption of juveniles could have a similar relevance in the balance of the diet to that of fruit consumption: both are sporadic food items that are only available during short periods and show similar proportions in diet.

In other lacertid lizards, as *Podarcis peloponnesiacus* [66] or *Timon lepidus* [72], plant matter is a sporadically eaten food. Obviously, this is not the case for the Balearic lizard, whose diet presents plant matter in all studied months. Consumption of certain plant species would be partially linked to their relative abundance. It seems to be the case of the sea fern, *C*. *maritimum*, a plant species intensively consumed by the Balearic lizard in several populations [73]. In Aire Island, sea ferns are present over the entire island's surface. They are particularly abundant in both peninsulae (North and West), deeply influenced by the proximity of the sea and where this plant species is dominant. Consequently, the consumption of sea fern was overriding in North and West areas. Fruits can be also an important food resource for lizards during some particular periods of the year. It is the case of the fruits of the dead horse arum. They constitute a main food item during June, coinciding with the ripening period of this plant species [55].

Our study was done with traditional techniques of prey and plant remains identification. Thus, we were able to recognize several prey items only at Order level. The molecular diet analysis is a new promising tool that can be employed in insular lizards. There is only an important limitation of molecular diet analysis. Due to differences in prey size, it is extremely difficult to quantify the relative contribution of each prey or plant species in the diet [74]. Next generation sequencing (NGS) provides a good compromise in terms of precision in prey identification, information obtained (DNA sequences) and prompts delivery of results [75]. Our dietary information would provide the required inventory of potential prey to design appropriate group-specific primers for a sound molecular diet analysis [75].

As in the case of other vertebrates [76], researchers aiming to understand the feeding ecology of small insular lizards should combine multiple approaches to studying diet, including the examination of faecal remains, direct observation of foraging behavior and DNA-based analysis of faecal samples [77].

In conclusion, the Balearic lizard living in Aire Island is able, not only to maintain a very dense population in an environment characterized by a low amount of trophic resources, but also to adapt its foraging behaviour to the variable offer along different years, seasons or areas of the islet. The important contribution to the diet of some prey items for a short period of time or in only a particular area of the island indicates an acute capacity to exploit ephemeral resources, as well as almost any new edible resource appearing in land.

## Supporting Information

S1 Dataset (XLSX)

S1 Fig Diet by volume during April in High area of Aire Island. (2006, 2008, 2009, 2010 and 2013).
(TIFF)

S2 Fig Diet by volume during May in High area of Aire Island. (2006, 2007, 2008, 2009, 2011 and 2012).
(TIFF)

S3 Fig Diet by volume during June in High area of Aire Island. (2007, 2008, 2009, 2010, 2011 and 2012).
(TIFF)

S4 Fig Diet by volume during July in High area of Aire Island. (1996, 2006, 2007, 2008, 2010, 2011, 2012 and 2013).
(TIFF)

S5 Fig Diet by volume during August in High area of Aire Island. (1995, 1997, 2000, 2006, 2009, 2010 and 2012).
(TIFF)

S6 Fig Variation of the diet by frequency in three months. (TIFF)

S7 Fig Variation of the diet by biomass in three months. (TIFF)

S1 Table Overall diet of 2009. Prey abundance (%) as the percentage of a given prey type in relation to the total prey number and relative prey presence (%p) as the percentage of faeces containing a given prey type. For plant matter we give its average (±SE) volume in the sample (see more details in the text).
(DOCX)

S2 Table Overall diet of 2011. (DOCX)

S3 Table Overall diet of 2012. (DOCX)

S4 Table Diet of High area. (DOCX)

S5 Table Diet of Low area. (DOCX)

S6 Table Diet of North area. Years 2009, 2011 and 2012.
(DOCX)

S7 Table Diet of West area. Years 2009, 2011 and 2012.
(DOCX)

S8 Table Diet of Jetty area. (DOCX)

S9 Table Diet of May. Years 2009, 2011 and 2012.
(DOCX)

S10 Table Diet of June. Years 2009, 2011 and 2012.
(DOCX)

S11 Table Diet of July. Years 2009, 2011 and 2012.
(DOCX)

S12 Table Diet of April in High area (2006). (DOCX)

S13 Table Diet of April in High area (2008). (DOCX)

S14 Table Diet of April in High area (2009). (DOCX)

S15 Table Diet of April in High area (2010). (DOCX)

S16 Table Diet of April in High area (2013). (DOCX)

S17 Table Diet of May in High area (2006). (DOCX)

S18 Table Diet of May in High area (2007). (DOCX)

S19 Table Diet of May in High area (2008). (DOCX)

S20 Table Diet of May in High area (2009). (DOCX)

S21 Table Diet of May in High area (2011). (DOCX)

S22 Table Diet of May in High area (2012). (DOCX)

S23 Table Diet of June in High area (2007). (DOCX)

S24 Table Diet of June in High area (2008). (DOCX)

S25 Table Diet of June in High area (2009). (DOCX)

S26 Table Diet of June in High area (2010). (DOCX)

S27 Table Diet of June in High area (2011). (DOCX)

S28 Table Diet of June in High area (2012). (DOCX)

S29 Table Diet of July in High area (1996). (DOCX)

S30 Table Diet of July in High area (1997). (DOCX)

S31 Table Diet of July in High area (2006). (DOCX)

S32 Table Diet of July in High area (2007). (DOCX)

S33 Table Diet of July in High area (2008). (DOCX)

S34 Table Diet of July in High area (2010). (DOCX)

S35 Table Diet of July in High area (2011). (DOCX)

S36 Table Diet of July in High area (2012). (DOCX)

S37 Table Diet of July in High area (2013). (DOCX)

S38 Table Diet of August in High area (1995). (DOCX)

S39 Table Diet of August in High area (1997). (DOCX)

S40 Table Diet of August in High area (2000). (DOCX)

S41 Table Diet of August in High area (2006). (DOCX)

S42 Table Diet of August in High area (2010). (DOCX)

S43 Table Diet of August in High area (2012). (DOCX)

S44 Table Prey availability and diet in High area during 2009, with values of D and E electivities. (DOCX)

S45 Table Prey availability and diet in Low area during 2009. (DOCX)

S46 Table Prey availability and diet in North area during 2009. (DOCX)

S47 Table Prey availability and diet in West area during 2009. (DOCX)

S48 Table Prey availability and diet in Jetty area during 2009. (DOCX)

S49 Table Prey availability and diet in High area during 2011. (DOCX)

S50 Table Prey availability and diet in Low area during 2011. (DOCX)

S51 Table Prey availability and diet in North area during 2011. (DOCX)

S52 Table Prey availability and diet in West area during 2011. (DOCX)

S53 Table Prey availability and diet in Jetty area during 2011. (DOCX)
