# Supplementary figures and images for: Omnivory of an Insular Lizard: Sources of Variation in the Diet of Podarcis lilfordi (Squamata, Lacertidae)
Source: PLoS One. 2016 Feb 12;11(2):e0148947. doi: 10.1371/journal.pone.0148947 (PMC4752353; doi:10.1371/journal.pone.0148947)

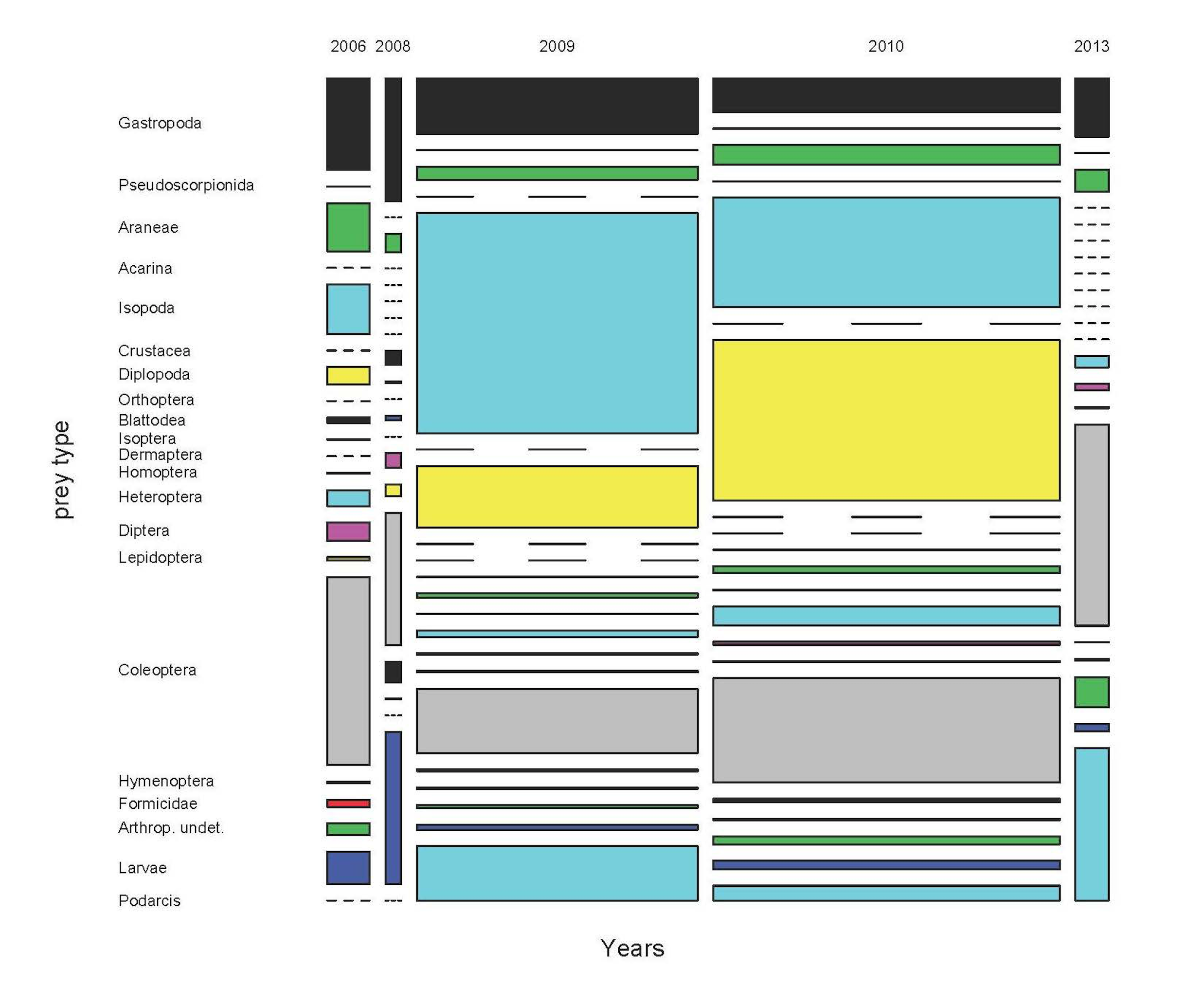

Supplement: S1 Fig — (2006, 2008, 2009, 2010 and 2013). (TIFF) [file pone.0148947.s002.tiff]

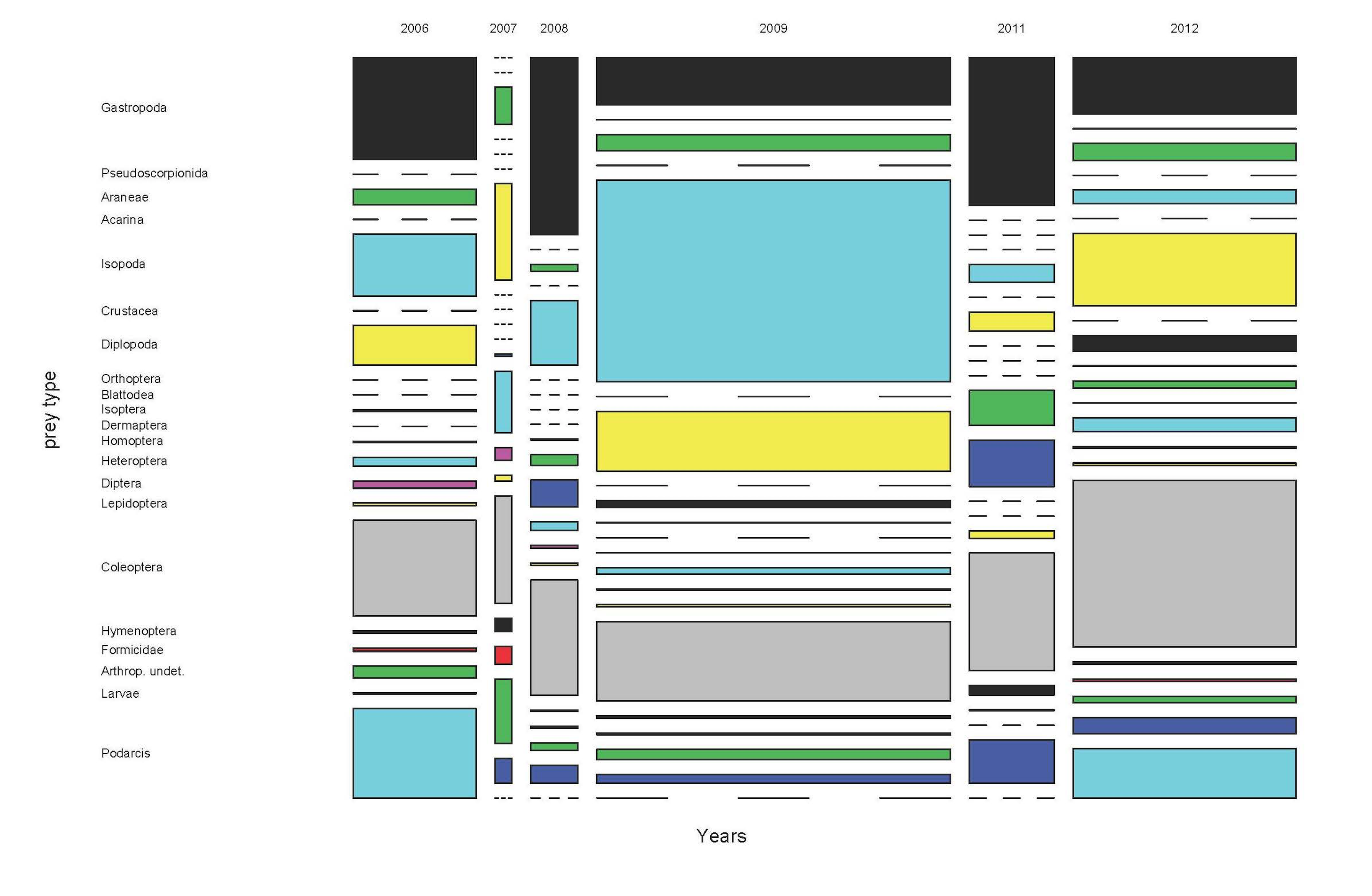

Supplement: S2 Fig — (2006, 2007, 2008, 2009, 2011 and 2012). (TIFF) [file pone.0148947.s003.tiff]

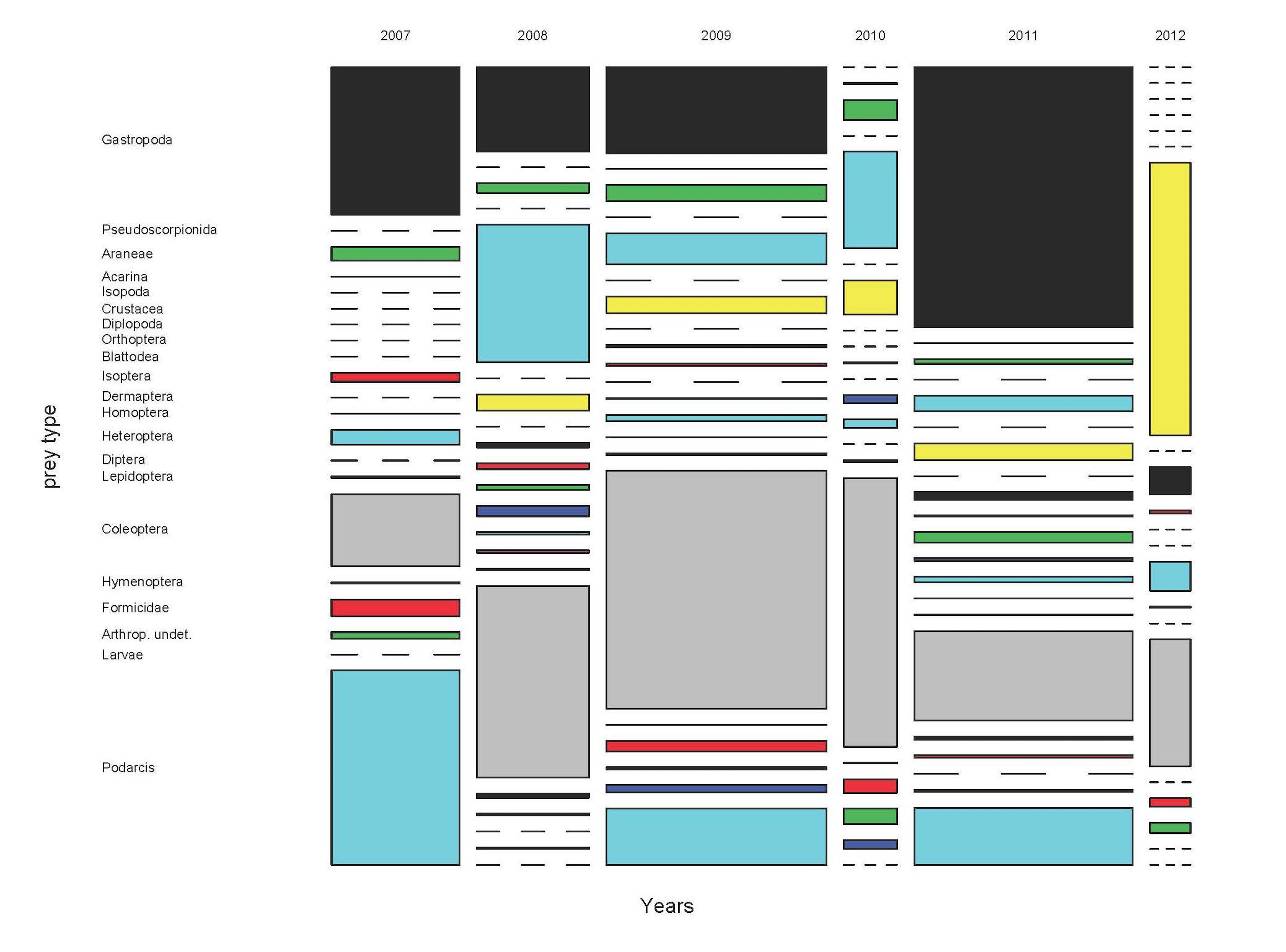

Supplement: S3 Fig — (2007, 2008, 2009, 2010, 2011 and 2012). (TIFF) [file pone.0148947.s004.tiff]

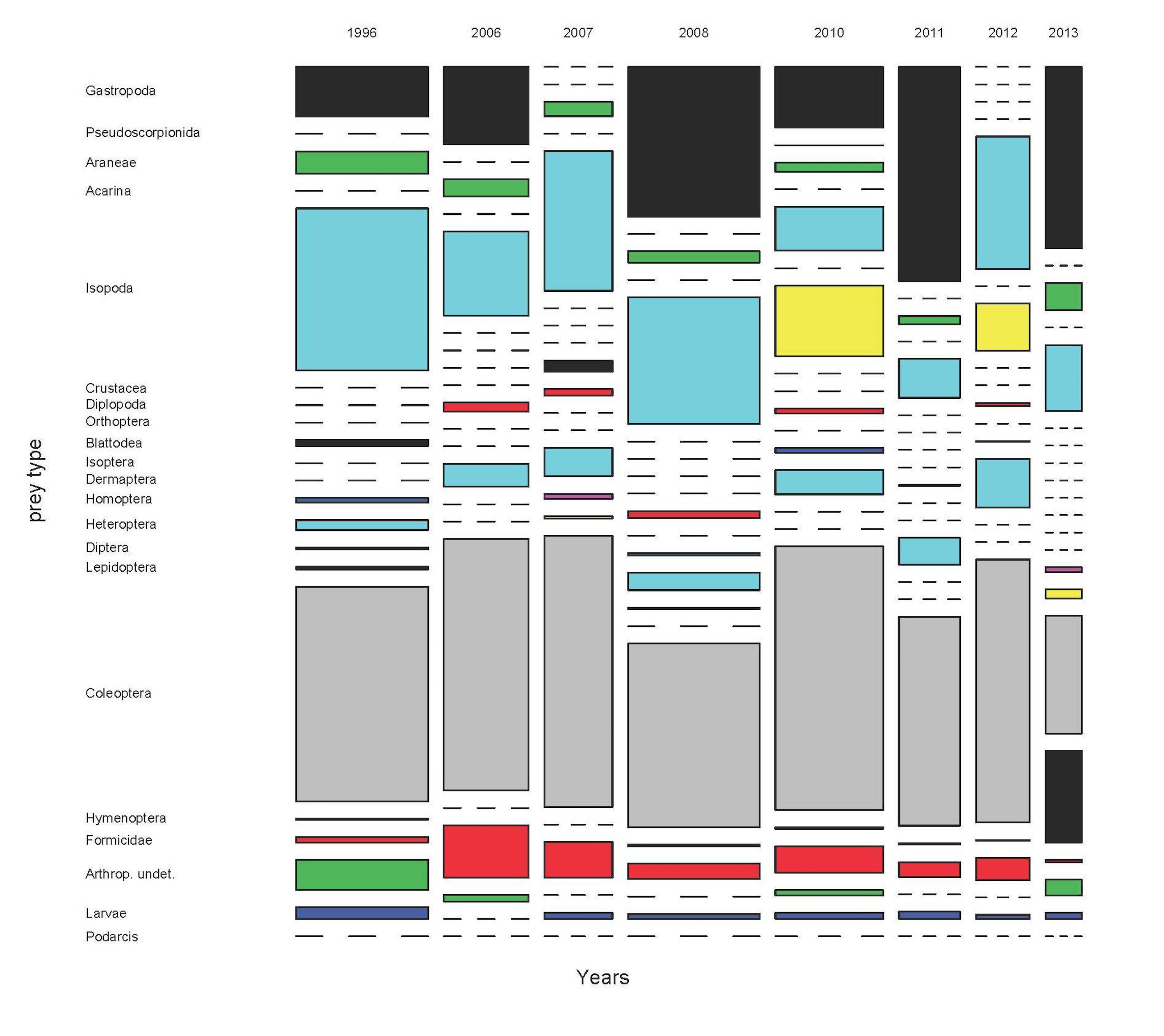

Supplement: S4 Fig — (1996, 2006, 2007, 2008, 2010, 2011, 2012 and 2013). (TIFF) [file pone.0148947.s005.tiff]

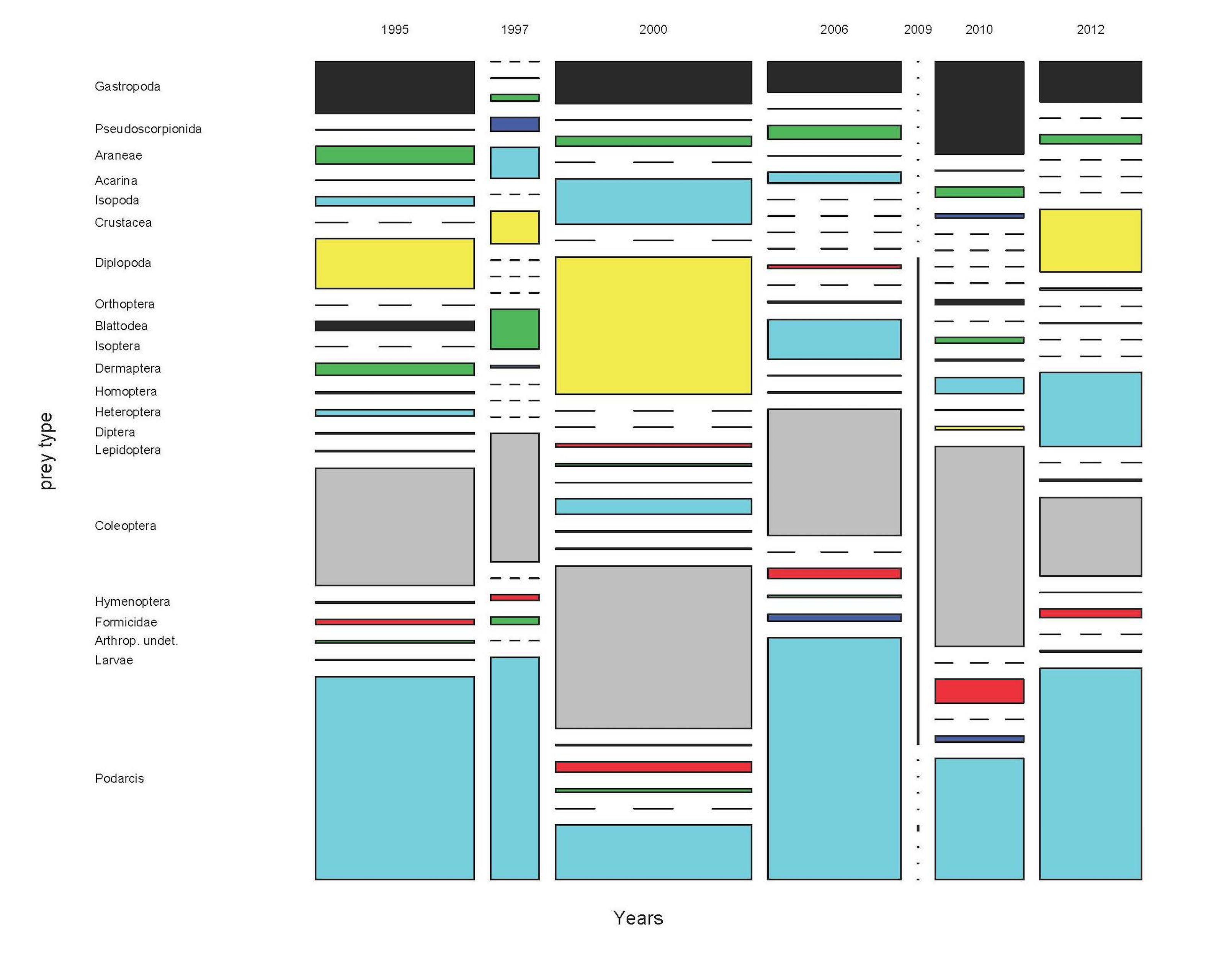

Supplement: S5 Fig — (1995, 1997, 2000, 2006, 2009, 2010 and 2012). (TIFF) [file pone.0148947.s006.tiff]

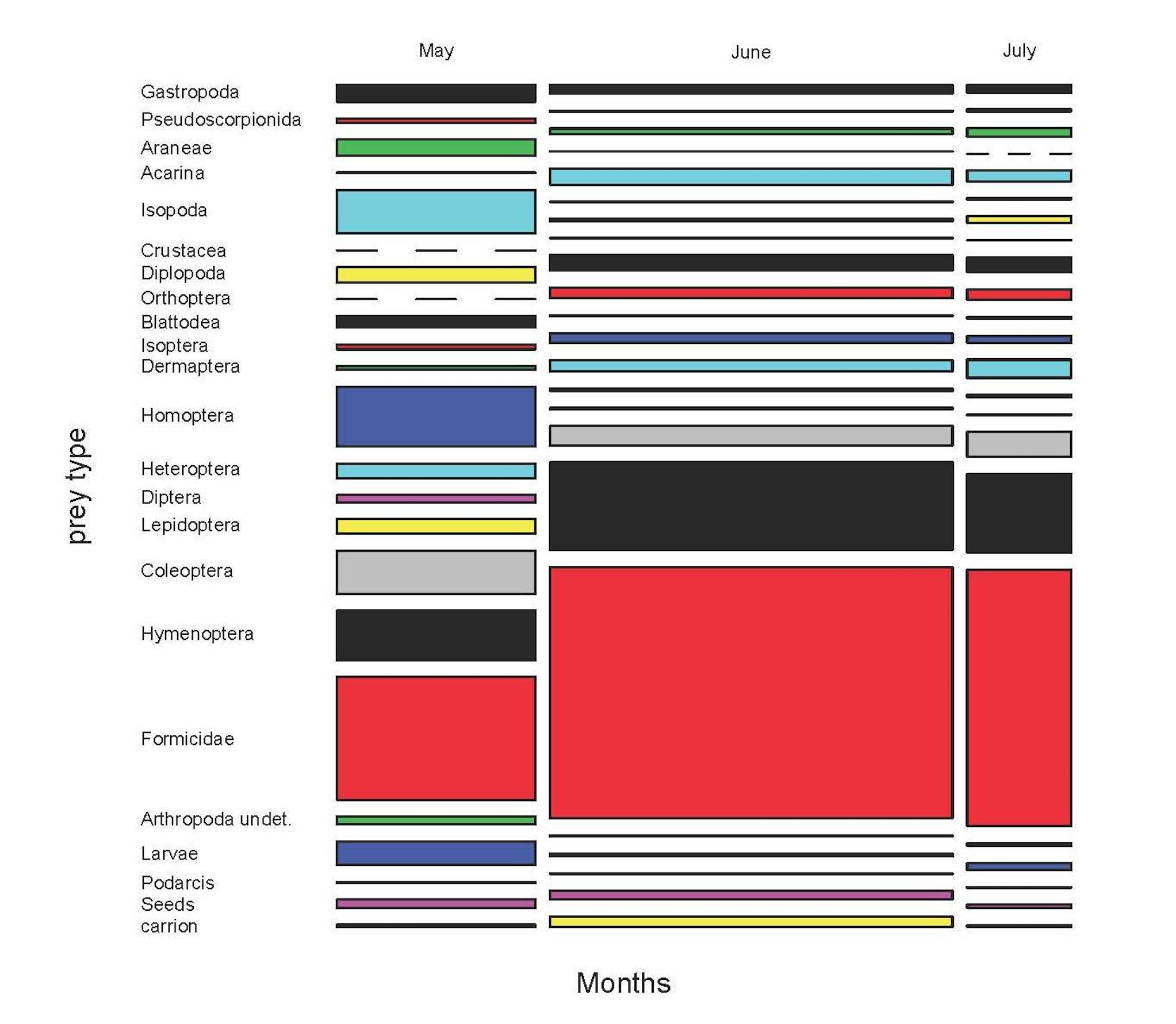

Supplement: S6 Fig — (TIFF) [file pone.0148947.s007.tiff]

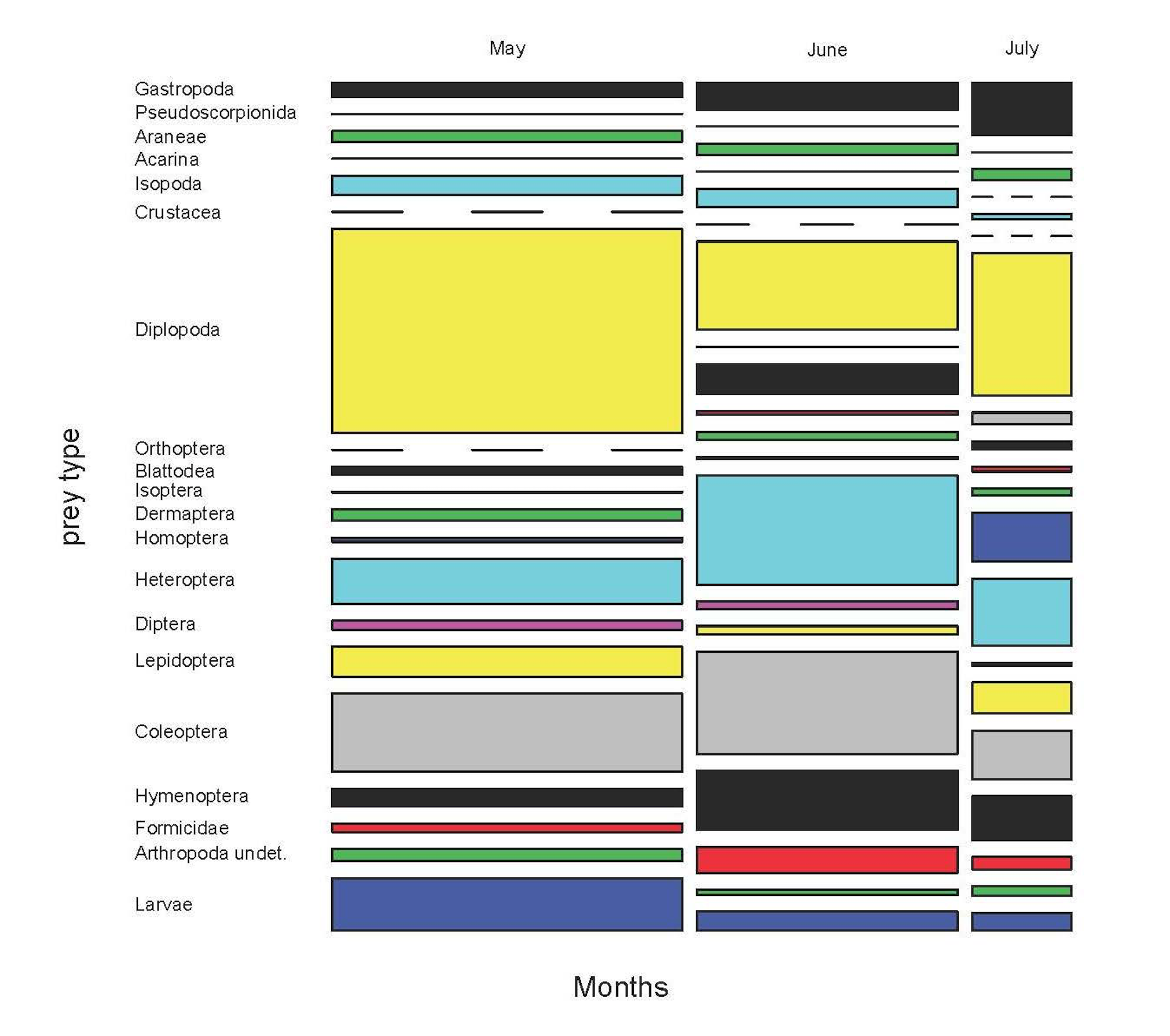

Supplement: S7 Fig — (TIFF) [file pone.0148947.s008.tiff]
