# Supplementary material for: Omnivory of an Insular Lizard: Sources of Variation in the Diet of Podarcis lilfordi (Squamata, Lacertidae)
Source: PLoS One. 2016 Feb 12;11(2):e0148947. doi: 10.1371/journal.pone.0148947 (PMC4752353; doi:10.1371/journal.pone.0148947)
Supplement: S1 Table — Prey abundance (%) as the percentage of a given prey type in relation to the total prey number and relative prey presence (%p) as the percentage of faeces containing a given prey type. For plant matter we give its average (±SE) volume in the sample (see more details in the text). (DOCX) [file pone.0148947.s009.docx]

| **Taxon** | **n** | **%n** | **presence** | **%presence** |
| --- | --- | --- | --- | --- |
| Gastropoda | 75 | 1.70 | 69 | 6.12 |
| Pseudoscorpionida | 20 | 0.45 | 18 | 1.60 |
| Araneae | 129 | 2.95 | 127 | 11.27 |
| Acarina | 5 | 0.11 | 4 | 0.35 |
| Isopoda | 365 | 8.27 | 356 | 31.59 |
| Crustaceae | 3 | 0.07 | 3 | 0.27 |
| Diplopoda | 61 | 1.38 | 61 | 5.41 |
| Blattodea | 74 | 1.68 | 66 | 5.86 |
| Isoptera | 72 | 1.63 | 61 | 5.41 |
| Dermaptera | 7 | 0.16 | 7 | 0.62 |
| Homoptera | 37 | 0.84 | 32 | 2.84 |
| Heteroptera | 131 | 2.97 | 123 | 10.91 |
| Diptera | 50 | 1.13 | 49 | 4.35 |
| Lepidoptera | 64 | 1.45 | 64 | 5.68 |
| Coleoptera | 283 | 6.41 | 222 | 19.70 |
| Hymenoptera | 359 | 8.13 | 104 | 9.23 |
| Formicidae | 2448 | 55.46 | 437 | 38.78 |
| Unidentif. Arthrop. | 43 | 0.97 | 43 | 3.82 |
| Larvae | 88 | 1.99 | 86 | 7.63 |
| *P. lilfordi* | 10 | 0.23 | 10 | 0.89 |
| Seeds | 80 | 1.81 | 74 | 6.57 |
| Carrion | 10 | 0.23 | 10 | 0.89 |
| Plant matter | 29.70 ± 1.18 |  | 565 | 50.13 |
| **Total** | **4414** | **100** | **1127** |  |
