# Supplementary material for: Omnivory of an Insular Lizard: Sources of Variation in the Diet of Podarcis lilfordi (Squamata, Lacertidae)
Source: PLoS One. 2016 Feb 12;11(2):e0148947. doi: 10.1371/journal.pone.0148947 (PMC4752353; doi:10.1371/journal.pone.0148947)
Supplement: S2 Table — (DOCX) [file pone.0148947.s010.docx]

| **Taxon** | **n** | **%n** | **presence** | **%presence** |
| --- | --- | --- | --- | --- |
| Gastropoda | 99 | 3.53 | 95 | 13.03 |
| Pseudoscorpionida | 16 | 0.57 | 16 | 2.19 |
| Araneae | 44 | 1.57 | 44 | 6.04 |
| Acarina | 1 | 0.04 | 1 | 0.14 |
| Isopoda | 89 | 3.18 | 89 | 12.21 |
| Crustaceae | 3 | 0.11 | 3 | 0.41 |
| Diplopoda | 36 | 1.28 | 36 | 4.94 |
| Orthoptera | 1 | 0.04 | 1 | 0.14 |
| Blattodea | 108 | 3.85 | 99 | 13.58 |
| Isoptera | 43 | 1.53 | 34 | 4.66 |
| Dermaptera | 19 | 0.68 | 14 | 1.92 |
| Homoptera | 357 | 12.74 | 132 | 18.11 |
| Heteroptera | 74 | 2.64 | 67 | 9.19 |
| Diptera | 29 | 1.03 | 29 | 3.98 |
| Lepidoptera | 27 | 0.96 | 26 | 3.57 |
| Coleoptera | 158 | 5.64 | 137 | 18.79 |
| Hymenoptera | 759 | 27.09 | 67 | 9.19 |
| Formicidae | 800 | 28.55 | 279 | 38.27 |
| Unidentif. Arthrop. | 11 | 0.39 | 11 | 1.51 |
| Larvae | 60 | 2.14 | 45 | 6.17 |
| *P. lilfordi* | 5 | 0.17 | 5 | 0.69 |
| Seeds | 56 | 2.00 | 44 | 6.04 |
| Carrion | 7 | 0.25 | 7 | 0.96 |
| Plant matter | 32.08 ± 1.52 |  | 379 | 51.99 |
| **Total** | **2802** | **100** | **729** |  |
