# Supplementary material for: Omnivory of an Insular Lizard: Sources of Variation in the Diet of Podarcis lilfordi (Squamata, Lacertidae)
Source: PLoS One. 2016 Feb 12;11(2):e0148947. doi: 10.1371/journal.pone.0148947 (PMC4752353; doi:10.1371/journal.pone.0148947)
Supplement: S3 Table — (DOCX) [file pone.0148947.s011.docx]

| **Taxon** | **n** | **%n** | **presence** | **%presence** |
| --- | --- | --- | --- | --- |
| Gastropoda | 55 | 2.46 | 46 | 6.87 |
| Pseudoscorpionida | 7 | 0.31 | 7 | 1.04 |
| Araneae | 40 | 1.79 | 40 | 5.97 |
| Acarina | 1 | 0.04 | 1 | 0.15 |
| Isopoda | 58 | 2.60 | 58 | 8.66 |
| Diplopoda | 79 | 3.54 | 79 | 11.79 |
| Orthoptera | 3 | 0.13 | 3 | 0.45 |
| Blattodea | 80 | 3.58 | 80 | 11.94 |
| Isoptera | 47 | 2.11 | 38 | 5.67 |
| Dermaptera | 11 | 0.49 | 10 | 1.49 |
| Homoptera | 22 | 0.99 | 22 | 3.28 |
| Heteroptera | 146 | 6.55 | 113 | 16.87 |
| Diptera | 27 | 1.21 | 27 | 4.03 |
| Lepidoptera | 17 | 0.76 | 17 | 2.54 |
| Coleoptera | 133 | 5.97 | 120 | 17.91 |
| Hymenoptera | 249 | 11.17 | 54 | 8.06 |
| Formicidae | 1036 | 46.48 | 278 | 41.49 |
| Unidentif. Arthrop. | 16 | 0.72 | 16 | 2.39 |
| Larvae | 56 | 2.51 | 56 | 8.36 |
| *P. lilfordi* | 5 | 0.22 | 5 | 0.75 |
| Seeds | 27 | 1.21 | 22 | 3.28 |
| Carrion | 114 | 5.11 | 15 | 2.24 |
| Plant matter | 39.12 ± 1.62 |  | 429 | 64.03 |
| **Total** | **2229** | **100** | **670** |  |
