# Supplementary material for: Omnivory of an Insular Lizard: Sources of Variation in the Diet of Podarcis lilfordi (Squamata, Lacertidae)
Source: PLoS One. 2016 Feb 12;11(2):e0148947. doi: 10.1371/journal.pone.0148947 (PMC4752353; doi:10.1371/journal.pone.0148947)
Supplement: S4 Table — (DOCX) [file pone.0148947.s012.docx]

| **Taxon** | **n** | **%n** | **presence** | **%presence** |
| --- | --- | --- | --- | --- |
| Gastropoda | 48 | 1.81 | 48 | 5.28 |
| Pseudoscorpionida | 8 | 0.3 | 8 | 0.88 |
| Araneae | 80 | 3.02 | 79 | 8.69 |
| Acarina | 0 | 0. | 0 | 0.00 |
| Isopoda | 155 | 5.85 | 152 | 16.72 |
| Crustaceae | 0 | 0 | 0 | 0.00 |
| Diplopoda | 75 | 2.83 | 75 | 8.25 |
| Orthoptera | 1 | 0.04 | 1 | 0.11 |
| Blattodea | 19 | 0.72 | 19 | 2.09 |
| Isoptera | 34 | 1.28 | 30 | 3.30 |
| Dermaptera | 18 | 0.68 | 15 | 1.65 |
| Homoptera | 241 | 9.1 | 71 | 7.81 |
| Heteroptera | 120 | 4.53 | 93 | 10.23 |
| Diptera | 42 | 1.59 | 41 | 4.51 |
| Lepidoptera | 38 | 1.43 | 37 | 4.07 |
| Coleoptera | 247 | 9.32 | 214 | 23.54 |
| Hymenoptera | 191 | 7.21 | 98 | 10.78 |
| Formicidae | 1117 | 42.15 | 338 | 37.18 |
| Unidentif. Arthrop. | 23 | 0.87 | 23 | 2.53 |
| Larvae | 119 | 4.49 | 106 | 11.66 |
| *P. lilfordi* | 8 | 0.3 | 8 | 0.88 |
| Seeds | 60 | 2.26 | 55 | 6.05 |
| Carrion | 6 | 0.23 | 6 | 0.66 |
| Plant matter | 39.14 ± 1.39 |  | 564 | 62.05 |
| **Total** | **2650** | **100** | **909** |  |
