# Supplementary material for: Omnivory of an Insular Lizard: Sources of Variation in the Diet of Podarcis lilfordi (Squamata, Lacertidae)
Source: PLoS One. 2016 Feb 12;11(2):e0148947. doi: 10.1371/journal.pone.0148947 (PMC4752353; doi:10.1371/journal.pone.0148947)
Supplement: S5 Table — (DOCX) [file pone.0148947.s013.docx]

| **Taxon** | **n** | **%n** | **presence** | **%presence** |
| --- | --- | --- | --- | --- |
| Gastropoda | 35 | 1.57 | 30 | 6.65 |
| Pseudoscorpionida | 12 | 0.54 | 12 | 2.66 |
| Araneae | 43 | 1.93 | 43 | 9.53 |
| Acarina | 4 | 0.18 | 3 | 0.67 |
| Isopoda | 89 | 4.00 | 88 | 19.51 |
| Crustaceae | 1 | 0.04 | 1 | 0.22 |
| Diplopoda | 33 | 1.48 | 33 | 7.32 |
| Orthoptera | 2 | 0.09 | 2 | 0.44 |
| Blattodea | 46 | 2.07 | 46 | 10.20 |
| Isoptera | 32 | 1.44 | 29 | 6.43 |
| Dermaptera | 10 | 0.45 | 8 | 1.77 |
| Homoptera | 63 | 2.83 | 39 | 8.65 |
| Heteroptera | 75 | 3.37 | 70 | 15.52 |
| Diptera | 18 | 0.81 | 18 | 3.99 |
| Lepidoptera | 29 | 1.30 | 29 | 6.43 |
| Coleoptera | 136 | 6.11 | 91 | 20.18 |
| Hymenoptera | 435 | 19.55 | 52 | 11.53 |
| Formicidae | 1077 | 48.38 | 179 | 39.69 |
| Unidentif. Arthrop. | 11 | 0.49 | 11 | 2.44 |
| Larvae | 27 | 1.21 | 26 | 5.77 |
| *P. lilfordi* | 5 | 0.22 | 5 | 1.11 |
| Seeds | 41 | 1.84 | 32 | 7.10 |
| Carrion | 2 | 0.09 | 2 | 0.44 |
| Plant matter | 39.14 ± 1.92 |  | 252 | 55.88 |
| **Total** | **2226** | **100** | **451** |  |
