# Supplementary material for: Omnivory of an Insular Lizard: Sources of Variation in the Diet of Podarcis lilfordi (Squamata, Lacertidae)
Source: PLoS One. 2016 Feb 12;11(2):e0148947. doi: 10.1371/journal.pone.0148947 (PMC4752353; doi:10.1371/journal.pone.0148947)
Supplement: S6 Table — Years 2009, 2011 and 2012. (DOCX) [file pone.0148947.s014.docx]

| **Taxon** | **n** | **%n** | **presence** | **%presence** |
| --- | --- | --- | --- | --- |
| Gastropoda | 28 | 1.63 | 26 | 7.62 |
| Pseudoscorpionida | 14 | 0.82 | 12 | 3.52 |
| Araneae | 27 | 1.57 | 27 | 7.92 |
| Acarina | 1 | 0.06 | 1 | 0.29 |
| Isopoda | 14 | 0.82 | 14 | 4.11 |
| Crustaceae | 2 | 0.12 | 2 | 0.59 |
| Diplopoda | 13 | 0.76 | 13 | 3.81 |
| Orthoptera | 0 | 0.00 | 0 | 0.00 |
| Blattodea | 117 | 6.81 | 104 | 30.50 |
| Isoptera | 29 | 1.69 | 24 | 7.04 |
| Dermaptera | 6 | 0.35 | 6 | 1.76 |
| Homoptera | 65 | 3.79 | 38 | 11.14 |
| Heteroptera | 54 | 3.14 | 51 | 14.96 |
| Diptera | 24 | 1.40 | 24 | 7.04 |
| Lepidoptera | 10 | 0.58 | 10 | 2.93 |
| Coleoptera | 59 | 3.44 | 55 | 16.13 |
| Hymenoptera | 420 | 24.46 | 29 | 8.50 |
| Formicidae | 794 | 46.24 | 147 | 43.11 |
| Unidentif. Arthrop. | 8 | 0.47 | 8 | 2.35 |
| Larvae | 21 | 1.22 | 18 | 5.28 |
| *P. lilfordi* | 2 | 0.12 | 2 | 0.59 |
| Seeds | 9 | 0.52 | 8 | 2.35 |
| Carrion | 0 | 0.00 | 0 | 0.00 |
| Plant matter | 25.79 ± 2.03 |  | 165 | 48.39 |
| **Total** | **1717** | **100** | **341** |  |
