# Supplementary material for: Omnivory of an Insular Lizard: Sources of Variation in the Diet of Podarcis lilfordi (Squamata, Lacertidae)
Source: PLoS One. 2016 Feb 12;11(2):e0148947. doi: 10.1371/journal.pone.0148947 (PMC4752353; doi:10.1371/journal.pone.0148947)
Supplement: S7 Table — Years 2009, 2011 and 2012. (DOCX) [file pone.0148947.s015.docx]

| **Taxon** | **n** | **%n** | **presence** | **%presence** |
| --- | --- | --- | --- | --- |
| Gastropoda | 81 | 5.02 | 72 | 14.49 |
| Pseudoscorpionida | 4 | 0.25 | 4 | 0.8 |
| Araneae | 43 | 2.67 | 43 | 8.65 |
| Acarina | 1 | 0.06 | 1 | 0.20 |
| Isopoda | 205 | 12.71 | 200 | 40.24 |
| Crustaceae | 1 | 0.06 | 1 | 0.20 |
| Diplopoda | 46 | 2.85 | 46 | 9.25 |
|  | 1 | 0.06 | 1 | 0.20 |
| Blattodea | 39 | 2.42 | 39 | 7.85 |
| Isoptera | 34 | 2.11 | 25 | 5.03 |
| Dermaptera | 0 | 0.0 | 0 | 0.00 |
| Homoptera | 16 | 0.99 | 13 | 2.62 |
| Heteroptera | 36 | 2.23 | 36 | 7.24 |
| Diptera | 9 | 0.56 | 9 | 1.81 |
| Lepidoptera | 16 | 0.99 | 16 | 3.22 |
| Coleoptera | 77 | 4.77 | 70 | 14.08 |
| Hymenoptera | 221 | 13.70 | 26 | 5.23 |
| Formicidae | 712 | 44.14 | 171 | 34.41 |
| Unidentif. Arthrop. | 20 | 1.24 | 20 | 4.02 |
| Larvae | 15 | 0.93 | 15 | 3.02 |
| *P. lilfordi* | 2 | 0.12 | 2 | 0.40 |
| Seeds | 21 | 1.30 | 19 | 3.82 |
| Carrion | 13 | 0.80 | 13 | 2.62 |
| Plant matter | 26.08 ± 1.71 |  | 225 | 45.27 |
| **Total** | **1613** | **100** | **497** |  |
