# Supplementary material for: Omnivory of an Insular Lizard: Sources of Variation in the Diet of Podarcis lilfordi (Squamata, Lacertidae)
Source: PLoS One. 2016 Feb 12;11(2):e0148947. doi: 10.1371/journal.pone.0148947 (PMC4752353; doi:10.1371/journal.pone.0148947)
Supplement: S8 Table — (DOCX) [file pone.0148947.s016.docx]

| **Taxon** | **n** | **%n** | **presence** | **%presence** |
| --- | --- | --- | --- | --- |
| Gastropoda | 36 | 2.92 | 33 | 10.15 |
| Pseudoscorpionida | 5 | 0.40 | 5 | 1.54 |
| Araneae | 20 | 1.62 | 19 | 5.85 |
| Acarina | 1 | 0.08 | 1 | 0.31 |
| Isopoda | 49 | 3.97 | 49 | 15.08 |
| Crustaceae | 2 | 0.16 | 2 | 0.61 |
| Diplopoda | 9 | 0.73 | 9 | 2.77 |
| Orthoptera | 0 | 0.00 | 0 | 0.00 |
| Blattodea | 41 | 3.33 | 37 | 11.38 |
| Isoptera | 33 | 2.67 | 25 | 7.69 |
| Dermaptera | 3 | 0.24 | 2 | 0.61 |
| Homoptera | 31 | 2.51 | 25 | 7.69 |
| Heteroptera | 65 | 5.27 | 52 | 16.00 |
| Diptera | 12 | 0.97 | 12 | 3.69 |
| Lepidoptera | 15 | 1.22 | 15 | 4.61 |
| Coleoptera | 55 | 4.46 | 49 | 15.08 |
| Hymenoptera | 98 | 7.95 | 18 | 5.54 |
| Formicidae | 584 | 47.36 | 159 | 48.92 |
| Unidentif. Arthrop. | 8 | 0.65 | 8 | 2.46 |
| Larvae | 21 | 1.70 | 21 | 6.46 |
| *P. lilfordi* | 3 | 0.24 | 3 | 0.92 |
| Seeds | 32 | 2.59 | 26 | 8.00 |
| Carrion | 110 | 8.92 | 11 | 3.39 |
| Plant matter | 33.69 ± 2.34 |  | 165 | 50.77 |
| **Total** | **1233** | **100** | **325** |  |
