# Supplementary material for: Omnivory of an Insular Lizard: Sources of Variation in the Diet of Podarcis lilfordi (Squamata, Lacertidae)
Source: PLoS One. 2016 Feb 12;11(2):e0148947. doi: 10.1371/journal.pone.0148947 (PMC4752353; doi:10.1371/journal.pone.0148947)
Supplement: S9 Table — Years 2009, 2011 and 2012. (DOCX) [file pone.0148947.s017.docx]

| **Taxon** | **n** | **%n** | **presence** | **%presence** |
| --- | --- | --- | --- | --- |
| Gastropoda | 81 | 3.53 | 528 | 8.31 |
| Pseudoscorpionida | 20 | 0.87 | 78 | 1.92 |
| Araneae | 78 | 3.41 | 18 | 8.31 |
| Acarina | 3 | 0.13 | 78 | 0.21 |
| Isopoda | 212 | 9.26 | 2 | 22.15 |
| Diplopoda | 73 | 3.19 | 208 | 7.77 |
| Blattodea | 56 | 2.45 | 73 | 5.96 |
| Isoptera | 24 | 1.05 | 56 | 1.92 |
| Dermaptera | 17 | 0.74 | 18 | 1.38 |
| Homoptera | 291 | 12.71 | 13 | 8.73 |
| Heteroptera | 72 | 3.15 | 82 | 7.35 |
| Diptera | 35 | 1.53 | 69 | 3.62 |
| Lepidoptera | 71 | 3.10 | 34 | 7.45 |
| Coleoptera | 210 | 9.17 | 70 | 17.89 |
| Hymenoptera | 240 | 10.48 | 168 | 11.93 |
| Formicidae | 596 | 26.04 | 112 | 28.33 |
| Unidentif. Arthrop. | 38 | 1.70 | 266 | 4.15 |
| Larvae | 115 | 5.02 | 39 | 10.54 |
| *P. lilfordi* | 4 | 0.17 | 99 | 0.43 |
| Seeds | 40 | 1.75 | 4 | 4.05 |
| Carrion | 12 | 0.52 | 38 | 1.28 |
| Plant matter | 35.56 ± 1.35 |  | 12 | 56.23 |
| **Total** | **2289** | **100** | **939** |  |
