# Supplementary material for: Omnivory of an Insular Lizard: Sources of Variation in the Diet of Podarcis lilfordi (Squamata, Lacertidae)
Source: PLoS One. 2016 Feb 12;11(2):e0148947. doi: 10.1371/journal.pone.0148947 (PMC4752353; doi:10.1371/journal.pone.0148947)
Supplement: S10 Table — Years 2009, 2011 and 2012. (DOCX) [file pone.0148947.s018.docx]

| **Taxon** | **n** | **%n** | **presence** | **%presence** |
| --- | --- | --- | --- | --- |
| Gastropoda | 84 | 1.81 | 79 | 10.66 |
| Pseudoscorpionida | 12 | 0.26 | 12 | 1.62 |
| Araneae | 58 | 1.25 | 57 | 7.69 |
| Acarina | 3 | 0.06 | 3 | 0.4 |
| Isopoda | 161 | 3.47 | 159 | 21.46 |
| Crustaceae | 2 | 0.04 | 2 | 0.27 |
| Diplopoda | 25 | 0.54 | 25 | 3.37 |
| Orthoptera | 1 | 0.02 | 1 | 0.14 |
| Blattodea | 154 | 3.32 | 138 | 18.62 |
| Isoptera | 107 | 2.31 | 88 | 11.88 |
| Dermaptera | 10 | 0.22 | 9 | 1.21 |
| Homoptera | 99 | 2.13 | 79 | 10.66 |
| Heteroptera | 114 | 2.46 | 107 | 14.44 |
| Diptera | 25 | 0.54 | 25 | 3.37 |
| Lepidoptera | 14 | 0.30 | 14 | 1.89 |
| Coleoptera | 190 | 4.10 | 150 | 20.24 |
| Hymenoptera | 864 | 18.63 | 64 | 8.64 |
| Formicidae | 2473 | 53.32 | 382 | 51.55 |
| Unidentif. Arthrop. | 12 | 0.26 | 12 | 1.62 |
| Larvae | 32 | 0.69 | 31 | 4.18 |
| *P. lilfordi* | 5 | 0.11 | 5 | 0.67 |
| Seeds | 87 | 1.86 | 77 | 10.39 |
| Carrion | 106 | 2.28 | 7 | 0.94 |
| Plant matter | 23.09 ± 1.35 |  | 308 | 41.56 |
| **Total** | **4638** | **100** | **741** |  |
