# Supplementary material for: Omnivory of an Insular Lizard: Sources of Variation in the Diet of Podarcis lilfordi (Squamata, Lacertidae)
Source: PLoS One. 2016 Feb 12;11(2):e0148947. doi: 10.1371/journal.pone.0148947 (PMC4752353; doi:10.1371/journal.pone.0148947)
Supplement: S11 Table — Years 2009, 2011 and 2012. (DOCX) [file pone.0148947.s019.docx]

| **Taxon** | **n** | **%n** | **presence** | **%presence** |
| --- | --- | --- | --- | --- |
| Gastropoda | 19 | 1.59 | 16 | 5.86 |
| Pseudoscorpionida | 5 | 0.42 | 5 | 1.83 |
| Araneae | 22 | 1.84 | 22 | 8.06 |
| Acarina | 0 | 0 | 0 | 0.00 |
| Isopoda | 27 | 2.26 | 27 | 9.89 |
| Crustaceae | 3 | 0.25 | 3 | 1.10 |
| Diplopoda | 18 | 1.5 | 18 | 6.59 |
| Orthoptera | 1 | 0.08 | 1 | 0.37 |
| Blattodea | 38 | 3.17 | 37 | 13.55 |
| Isoptera | 28 | 2.34 | 24 | 8.79 |
| Dermaptera | 5 | 0.42 | 4 | 1.46 |
| Homoptera | 18 | 1.5 | 18 | 6.59 |
| Heteroptera | 46 | 3.84 | 45 | 16.48 |
| Diptera | 7 | 0.58 | 7 | 2.56 |
| Lepidoptera | 2 | 0.17 | 2 | 0.73 |
| Coleoptera | 64 | 5.35 | 58 | 21.25 |
| Hymenoptera | 200 | 16.71 | 14 | 5.13 |
| Formicidae | 651 | 54.39 | 151 | 55.31 |
| Unidentif. Arthrop. | 8 | 0.67 | 8 | 2.93 |
| Larvae | 18 | 1.5 | 18 | 6.59 |
| *P. lilfordi* | 2 | 0.17 | 2 | 0.73 |
| Seeds | 9 | 0.75 | 6 | 2.2 |
| Carrion | 6 | 0.5 | 6 | 2.2 |
| Plant matter | 38.78 ± 2.46 |  | 182 | 66.67 |
| **Total** | **1197** | **100** | **273** |  |
