# Supplementary material for: Omnivory of an Insular Lizard: Sources of Variation in the Diet of Podarcis lilfordi (Squamata, Lacertidae)
Source: PLoS One. 2016 Feb 12;11(2):e0148947. doi: 10.1371/journal.pone.0148947 (PMC4752353; doi:10.1371/journal.pone.0148947)
Supplement: S12 Table — (DOCX) [file pone.0148947.s020.docx]

| **Taxon** | **n** | **%n** | **presence** | **%presence** |
| --- | --- | --- | --- | --- |
| Gastropoda | 2 | 0.84 | 2 | 1.32 |
| Pseudoscorpionida | 2 | 0.84 | 2 | 1.32 |
| Araneae | 14 | 5.86 | 14 | 9.21 |
| Acarina | 0 | 0 | 0 | 0 |
| Isopoda | 3 | 1.26 | 3 | 1.97 |
| Crustaceae | 0 | 0 | 0 | 0 |
| Diplopoda | 1 | 0.42 | 1 | 0.66 |
| Orthoptera | 0 | 0 | 0 | 0 |
| Blattodea | 1 | 0.42 | 1 | 0.66 |
| Isoptera | 1 | 0.42 | 1 | 0.66 |
| Dermaptera | 0 | 0 | 0 | 0 |
| Homoptera | 5 | 2.09 | 4 | 2.63 |
| Heteroptera | 7 | 2.93 | 6 | 3.95 |
| Diptera | 46 | 19.25 | 41 | 26.97 |
| Lepidoptera | 3 | 1.26 | 3 | 1.97 |
| Coleoptera | 19 | 7.95 | 18 | 11.84 |
| Hymenoptera | 8 | 3.35 | 8 | 5.26 |
| Formicidae | 100 | 41.84 | 48 | 31.58 |
| Unidentif. Arthrop. | 3 | 1.26 | 3 | 1.97 |
| Larvae | 21 | 8.79 | 21 | 13.82 |
| *P. lilfordi* | 0 | 0 | 0 | 0 |
| Seeds | 3 | 1.26 | 2 | 1.32 |
| Carrion | 0 | 0 | 0 | 0 |
| Plant matter | 50.55 ± 3.62 |  | 102 | 67.11 |
| **Total** | **239** | **100** | **152** |  |
