# Supplementary material for: Omnivory of an Insular Lizard: Sources of Variation in the Diet of Podarcis lilfordi (Squamata, Lacertidae)
Source: PLoS One. 2016 Feb 12;11(2):e0148947. doi: 10.1371/journal.pone.0148947 (PMC4752353; doi:10.1371/journal.pone.0148947)
Supplement: S13 Table — (DOCX) [file pone.0148947.s021.docx]

| **Taxon** | **n** | **%n** | **presence** | **%presence** |
| --- | --- | --- | --- | --- |
| Gastropoda | 1 | 0.92 | 1 | 1.85 |
| Pseudoscorpionida | 0 | 0 | 0 | 0 |
| Araneae | 2 | 1.83 | 2 | 3.70 |
| Acarina | 0 | 0 | 0 | 0 |
| Isopoda | 0 | 0 | 0 | 0 |
| Crustaceae | 0 | 0 | 0 | 0 |
| Diplopoda | 0 | 0 | 0 | 0 |
| Orthoptera | 0 | 0 | 0 | 0 |
| Blattodea | 1 | 0.92 | 1 | 1.85 |
| Isoptera | 1 | 0.92 | 1 | 1.85 |
| Dermaptera | 0 | 0 | 0 | 0 |
| Homoptera | 8 | 7.34 | 5 | 9.26 |
| Heteroptera | 0 | 0 | 0 | 0 |
| Diptera | 14 | 12.84 | 14 | 25.93 |
| Lepidoptera | 4 | 3.67 | 4 | 7.41 |
| Coleoptera | 5 | 4.59 | 3 | 5.56 |
| Hymenoptera | 34 | 31.19 | 22 | 40.74 |
| Formicidae | 1 | 0.92 | 1 | 1.85 |
| Unidentif. Arthrop. | 0 | 0 | 0 | 0 |
| Larvae | 37 | 33.94 | 35 | 64.81 |
| *P. lilfordi* | 0 | 0 | 0 | 0 |
| Seeds | 1 | 0.92 | 1 | 1.85 |
| Carrion | 0 | 0 | 0 | 0 |
| Plant matter | 6.65 ± 2.44 |  | 17 | 31.48 |
| **Total** | **109** | **100** | **54** |  |
