# Supplementary material for: Omnivory of an Insular Lizard: Sources of Variation in the Diet of Podarcis lilfordi (Squamata, Lacertidae)
Source: PLoS One. 2016 Feb 12;11(2):e0148947. doi: 10.1371/journal.pone.0148947 (PMC4752353; doi:10.1371/journal.pone.0148947)
Supplement: S14 Table — (DOCX) [file pone.0148947.s022.docx]

| **Taxon** | **n** | **%n** | **presence** | **%presence** |
| --- | --- | --- | --- | --- |
| Gastropoda | 8 | 1.84 | 8 | 3.38 |
| Pseudoscorpionida | 2 | 0.46 | 2 | 0.84 |
| Araneae | 26 | 5.99 | 25 | 10.55 |
| Acarina | 0 | 0 | 0 | 0 |
| Isopoda | 88 | 20.28 | 85 | 35.86 |
| Crustaceae | 0 | 0 | 0 | 0 |
| Diplopoda | 23 | 5.30 | 23 | 9.70 |
| Orthoptera | 0 | 0 | 0 | 0 |
| Blattodea | 0 | 0 | 0 | 0 |
| Isoptera | 1 | 0.23 | 1 | 0.42 |
| Dermaptera | 5 | 1.15 | 5 | 2.11 |
| Homoptera | 3 | 0.69 | 2 | 0.84 |
| Heteroptera | 19 | 4.38 | 15 | 6.33 |
| Diptera | 16 | 3.69 | 16 | 6.75 |
| Lepidoptera | 10 | 2.30 | 10 | 4.22 |
| Coleoptera | 43 | 9.91 | 42 | 17.72 |
| Hymenoptera | 46 | 10.60 | 31 | 13.08 |
| Formicidae | 110 | 25.35 | 63 | 26.58 |
| Unidentif. Arthrop. | 5 | 1.15 | 5 | 2.11 |
| Larvae | 22 | 5.07 | 22 | 9.28 |
| *P. lilfordi* | 3 | 0.69 | 3 | 1.27 |
| Seeds | 3 | 0.69 | 3 | 1.27 |
| Carrion | 1 | 0.23 | 1 | 0.42 |
| Plant matter | 37.12 ± 2.69 |  | 146 | 61.60 |
| **Total** | **434** | **100** | **237** |  |
