# Supplementary material for: Omnivory of an Insular Lizard: Sources of Variation in the Diet of Podarcis lilfordi (Squamata, Lacertidae)
Source: PLoS One. 2016 Feb 12;11(2):e0148947. doi: 10.1371/journal.pone.0148947 (PMC4752353; doi:10.1371/journal.pone.0148947)
Supplement: S15 Table — (DOCX) [file pone.0148947.s023.docx]

| **Taxon** | **n** | **%n** | **presence** | **%presence** |
| --- | --- | --- | --- | --- |
| Gastropoda | 6 | 0.74 | 6 | 1.51 |
| Pseudoscorpionida | 5 | 0.62 | 5 | 1.26 |
| Araneae | 47 | 5.78 | 47 | 11.84 |
| Acarina | 4 | 0.49 | 4 | 1.01 |
| Isopoda | 54 | 6.64 | 54 | 13.60 |
| Crustaceae | 0 | 0 | 0 | 0 |
| Diplopoda | 74 | 9.10 | 73 | 18.39 |
| Orthoptera | 0 | 0 | 0 | 0 |
| Blattodea | 0 | 0 | 0 | 0 |
| Isoptera | 1 | 0.12 | 1 | 0.25 |
| Dermaptera | 10 | 1.23 | 9 | 2.27 |
| Homoptera | 35 | 4.31 | 31 | 7.81 |
| Heteroptera | 67 | 8.24 | 67 | 16.88 |
| Diptera | 61 | 7.50 | 42 | 10.58 |
| Lepidoptera | 5 | 0.62 | 5 | 1.26 |
| Coleoptera | 86 | 10.58 | 76 | 19.14 |
| Hymenoptera | 122 | 15.01 | 78 | 19.65 |
| Formicidae | 165 | 20.30 | 106 | 26.70 |
| Unidentif. Arthrop. | 16 | 1.97 | 16 | 4.03 |
| Larvae | 46 | 5.66 | 45 | 11.34 |
| *P. lilfordi* | 1 | 0.12 | 1 | 0.25 |
| Seeds | 2 | 0.25 | 1 | 0.25 |
| Carrion | 6 | 0.74 | 6 | 1.51 |
| Plant matter | 37.22 ± 2.01 |  | 272 | 68.51 |
| **Total** | **813** | **100** | **397** |  |
