# Supplementary material for: Omnivory of an Insular Lizard: Sources of Variation in the Diet of Podarcis lilfordi (Squamata, Lacertidae)
Source: PLoS One. 2016 Feb 12;11(2):e0148947. doi: 10.1371/journal.pone.0148947 (PMC4752353; doi:10.1371/journal.pone.0148947)
Supplement: S16 Table — (DOCX) [file pone.0148947.s024.docx]

| **Taxon** | **n** | **%n** | **presence** | **%presence** |
| --- | --- | --- | --- | --- |
| Gastropoda | 1 | 1.39 | 1 | 2.04 |
| Pseudoscorpionida | 1 | 1.39 | 1 | 2.04 |
| Araneae | 5 | 6.94 | 4 | 8.16 |
| Acarina | 0 | 0 | 0 | 0 |
| Isopoda | 0 | 0 | 0 | 0 |
| Crustaceae | 0 | 0 | 0 | 0 |
| Diplopoda | 0 | 0 | 0 | 0 |
| Orthoptera | 0 | 0 | 0 | 0 |
| Blattodea | 0 | 0 | 0 | 0 |
| Isoptera | 0 | 0 | 0 | 0 |
| Dermaptera | 0 | 0 | 0 | 0 |
| Homoptera | 0 | 0 | 0 | 0 |
| Heteroptera | 4 | 5.56 | 4 | 8.16 |
| Diptera | 13 | 18.06 | 12 | 24.49 |
| Lepidoptera | 1 | 1.39 | 1 | 2.04 |
| Coleoptera | 16 | 22.32 | 14 | 28.57 |
| Hymenoptera | 1 | 1.39 | 1 | 2.04 |
| Formicidae | 19 | 26.39 | 13 | 26.53 |
| Unidentif. Arthrop. | 6 | 8.33 | 5 | 10.20 |
| Larvae | 4 | 5.56 | 4 | 8.16 |
| *P. lilfordi* | 1 | 1.39 | 1 | 2.04 |
| Seeds | 0 | 0 | 0 | 0 |
| Carrion | 0 | 0 | 0 | 0 |
| Plant matter | 78.65 ± 4.25 |  | 45 | 91.84 |
| **Total** | **72** | **100** | **49** |  |
