# Supplementary material for: Omnivory of an Insular Lizard: Sources of Variation in the Diet of Podarcis lilfordi (Squamata, Lacertidae)
Source: PLoS One. 2016 Feb 12;11(2):e0148947. doi: 10.1371/journal.pone.0148947 (PMC4752353; doi:10.1371/journal.pone.0148947)
Supplement: S17 Table — (DOCX) [file pone.0148947.s025.docx]

| **Taxon** | **n** | **%n** | **presence** | **%presence** |
| --- | --- | --- | --- | --- |
| Gastropoda | 3 | 1.69 | 3 | 3.13 |
| Pseudoscorpionida | 0 | 0 | 0 | 0 |
| Araneae | 6 | 3.39 | 6 | 6.25 |
| Acarina | 0 | 0 | 0 | 0 |
| Isopoda | 5 | 2.82 | 5 | 5.21 |
| Crustaceae | 0 | 0 | 0 | 0 |
| Diplopoda | 3 | 1.69 | 3 | 3.13 |
| Orthoptera | 0 | 0 | 0 | 0 |
| Blattodea | 0 | 0 | 0 | 0 |
| Isoptera | 6 | 3.39 | 6 | 6.25 |
| Dermaptera | 0 | 0 | 0 | 0 |
| Homoptera | 10 | 5.65 | 8 | 8.33 |
| Heteroptera | 5 | 2.82 | 5 | 5.21 |
| Diptera | 25 | 14.12 | 20 | 20.83 |
| Lepidoptera | 3 | 1.69 | 3 | 3.13 |
| Coleoptera | 13 | 7.34 | 13 | 13.54 |
| Hymenoptera | 14 | 7.91 | 8 | 8.33 |
| Formicidae | 61 | 34.46 | 35 | 36.46 |
| Unidentif. Arthrop. | 4 | 2.26 | 4 | 4.17 |
| Larvae | 1 | 0.56 | 1 | 1.04 |
| *P. lilfordi* | 1 | 0.56 | 1 | 1.04 |
| Seeds | 17 | 9.60 | 14 | 14.58 |
| Carrion | 0 | 0 | 0 | 0 |
| Plant matter | 40.69 ± 4.46 |  | 58 | 60.42 |
| **Total** | **177** | **100** | **96** |  |
