# Supplementary material for: Omnivory of an Insular Lizard: Sources of Variation in the Diet of Podarcis lilfordi (Squamata, Lacertidae)
Source: PLoS One. 2016 Feb 12;11(2):e0148947. doi: 10.1371/journal.pone.0148947 (PMC4752353; doi:10.1371/journal.pone.0148947)
Supplement: S18 Table — (DOCX) [file pone.0148947.s026.docx]

| **Taxon** | **n** | **%n** | **presence** | **%presence** |
| --- | --- | --- | --- | --- |
| Gastropoda | 0 | 0 | 0 | 0 |
| Pseudoscorpionida | 0 | 0 | 0 | 0 |
| Araneae | 2 | 2.27 | 2 | 4.08 |
| Acarina | 0 | 0 | 0 | 0 |
| Isopoda | 0 | 0 | 0 | 0 |
| Crustaceae | 0 | 0 | 0 | 0 |
| Diplopoda | 1 | 1.14 | 1 | 2.04 |
| Orthoptera | 0 | 0 | 0 | 0 |
| Blattodea | 0 | 0 | 0 | 0 |
| Isoptera | 0 | 0 | 0 | 0 |
| Dermaptera | 0 | 0 | 0 | 0 |
| Homoptera | 2 | 2.27 | 2 | 4.08 |
| Heteroptera | 5 | 5.68 | 4 | 8.16 |
| Diptera | 6 | 6.82 | 6 | 12.24 |
| Lepidoptera | 1 | 1.14 | 1 | 2.04 |
| Coleoptera | 2 | 2.27 | 2 | 4.08 |
| Hymenoptera | 11 | 12.50 | 10 | 20.41 |
| Formicidae | 46 | 52.27 | 23 | 46.94 |
| Unidentif. Arthrop. | 3 | 3.41 | 3 | 6.12 |
| Larvae | 3 | 3.41 | 3 | 6.12 |
| *P. lilfordi* | 0 | 0 | 0 | 0 |
| Seeds | 5 | 5.68 | 3 | 6.12 |
| Carrion | 1 | 1.14 | 1 | 2.04 |
| Plant matter | 67.98 ± 6.23 |  | 36 | 73.47 |
| **Total** | **88** | **100** | **49** |  |
