# Supplementary material for: Omnivory of an Insular Lizard: Sources of Variation in the Diet of Podarcis lilfordi (Squamata, Lacertidae)
Source: PLoS One. 2016 Feb 12;11(2):e0148947. doi: 10.1371/journal.pone.0148947 (PMC4752353; doi:10.1371/journal.pone.0148947)
Supplement: S19 Table — (DOCX) [file pone.0148947.s027.docx]

| **Taxon** | **n** | **%n** | **presence** | **%presence** |
| --- | --- | --- | --- | --- |
| Gastropoda | 2 | 1.52 | 2 | 3.64 |
| Pseudoscorpionida | 0 | 0 | 0 | 0 |
| Araneae | 1 | 0.76 | 1 | 1.82 |
| Acarina | 0 | 0 | 0 | 0 |
| Isopoda | 2 | 1.52 | 2 | 3.64 |
| Crustaceae | 0 | 0 | 0 | 0 |
| Diplopoda | 0 | 0 | 0 | 0 |
| Orthoptera | 0 | 0 | 0 | 0 |
| Blattodea | 0 | 0 | 0 | 0 |
| Isoptera | 1 | 0.76 | 1 | 1.82 |
| Dermaptera | 1 | 0.76 | 1 | 1.82 |
| Homoptera | 62 | 46.97 | 26 | 47.27 |
| Heteroptera | 2 | 1.52 | 2 | 3.64 |
| Diptera | 4 | 3.03 | 4 | 7.27 |
| Lepidoptera | 1 | 0.76 | 1 | 1.82 |
| Coleoptera | 6 | 4.55 | 6 | 10.91 |
| Hymenoptera | 3 | 2.72 | 1 | 1.82 |
| Formicidae | 13 | 9.85 | 12 | 21.82 |
| Unidentif. Arthrop. | 1 | 0.76 | 1 | 1.82 |
| Larvae | 6 | 4.55 | 6 | 10.91 |
| *P. lilfordi* | 0 | 0 | 0 | 0 |
| Seeds | 26 | 19.70 | 19 | 35.54 |
| Carrion | 1 | 0.76 | 1 | 1.82 |
| Plant matter | 19.05 ± 4.98 |  | 14 | 25.45 |
| **Total** | **132** | **100** | **55** |  |
