# Supplementary material for: Omnivory of an Insular Lizard: Sources of Variation in the Diet of Podarcis lilfordi (Squamata, Lacertidae)
Source: PLoS One. 2016 Feb 12;11(2):e0148947. doi: 10.1371/journal.pone.0148947 (PMC4752353; doi:10.1371/journal.pone.0148947)
Supplement: S20 Table — (DOCX) [file pone.0148947.s028.docx]

| **Taxon** | **n** | **%n** | **presence** | **%presence** |
| --- | --- | --- | --- | --- |
| Gastropoda | 4 | 1.20 | 4 | 2.30 |
| Pseudoscorpionida | 3 | 0.90 | 3 | 1.72 |
| Araneae | 18 | 5.41 | 18 | 10.34 |
| Acarina | 0 | 0 | 0 | 0 |
| Isopoda | 47 | 14.11 | 47 | 27.01 |
| Crustaceae | 0 | 0 | 0 | 0 |
| Diplopoda | 13 | 3.90 | 13 | 7.47 |
| Orthoptera | 0 | 0 | 0 | 0 |
| Blattodea | 5 | 1.50 | 5 | 2.87 |
| Isoptera | 6 | 1.80 | 5 | 2.87 |
| Dermaptera | 0 | 0 | 0 | 0 |
| Homoptera | 6 | 1.80 | 5 | 2.87 |
| Heteroptera | 11 | 3.30 | 11 | 6.32 |
| Diptera | 8 | 2.40 | 7 | 4.02 |
| Lepidoptera | 7 | 2.10 | 7 | 4.02 |
| Coleoptera | 31 | 9.31 | 31 | 17.82 |
| Hymenoptera | 44 | 13.21 | 21 | 12.07 |
| Formicidae | 84 | 25.23 | 53 | 30.46 |
| Unidentif. Arthrop. | 10 | 3.00 | 10 | 5.75 |
| Larvae | 22 | 6.61 | 22 | 12.64 |
| *P. lilfordi* | 0 | 0 | 0 | 0 |
| Seeds | 13 | 3.90 | 13 | 7.47 |
| Carrion | 1 | 0.30 | 1 | 0.57 |
| Plant matter | 51.11 ± 3.21 |  | 127 | 72.99 |
| **Total** | **333** | **1001** | **174** |  |
