# Supplementary material for: Omnivory of an Insular Lizard: Sources of Variation in the Diet of Podarcis lilfordi (Squamata, Lacertidae)
Source: PLoS One. 2016 Feb 12;11(2):e0148947. doi: 10.1371/journal.pone.0148947 (PMC4752353; doi:10.1371/journal.pone.0148947)
Supplement: S21 Table — (DOCX) [file pone.0148947.s029.docx]

| **Taxon** | **n** | **%n** | **presence** | **%presence** |
| --- | --- | --- | --- | --- |
| Gastropoda | 3 | 0.99 | 3 | 6 |
| Pseudoscorpionida | 0 | 0 | 0 | 0 |
| Araneae | 0 | 0 | 0 | 0 |
| Acarina | 0 | 0 | 0 | 0 |
| Isopoda | 1 | 0.33 | 1 | 2 |
| Crustaceae | 0 | 0 | 0 | 0 |
| Diplopoda | 1 | 0.33 | 1 | 2 |
| Orthoptera | 0 | 0 | 0 | 0 |
| Blattodea | 0 | 0 | 0 | 0 |
| Isoptera | 0 | 0 | 0 | 0 |
| Dermaptera | 6 | 1.99 | 3 | 6 |
| Homoptera | 194 | 64.24 | 34 | 68 |
| Heteroptera | 0 | 0 | 0 | 0 |
| Diptera | 0 | 0 | 0 | 0 |
| Lepidoptera | 6 | 1.99 | 5 | 10 |
| Coleoptera | 11 | 3.64 | 8 | 16 |
| Hymenoptera | 40 | 13.25 | 9 | 18 |
| Formicidae | 9 | 2.98 | 7 | 14 |
| Unidentif. Arthrop. | 0 | 0 | 0 | 0 |
| Larvae | 26 | 8.61 | 14 | 28 |
| *P. lilfordi* | 0 | 0 | 0 | 0 |
| Seeds | 5 | 1.66 | 5 | 10 |
| Carrion | 0 | 0 | 0 | 0 |
| Plant matter | 22.94 ± 5.17 |  | 22 | 44 |
| **Total** | **302** | **1001** | **50** |  |
