# Supplementary material for: Omnivory of an Insular Lizard: Sources of Variation in the Diet of Podarcis lilfordi (Squamata, Lacertidae)
Source: PLoS One. 2016 Feb 12;11(2):e0148947. doi: 10.1371/journal.pone.0148947 (PMC4752353; doi:10.1371/journal.pone.0148947)
Supplement: S22 Table — (DOCX) [file pone.0148947.s030.docx]

| **Taxon** | **n** | **%n** | **presence** | **%presence** |
| --- | --- | --- | --- | --- |
| Gastropoda | 3 | 1.19 | 3 | 2.04 |
| Pseudoscorpionida | 1 | 0.40 | 1 | 0.68 |
| Araneae | 12 | 4.74 | 12 | 8.16 |
| Acarina | 0 | 0 | 0 | 0 |
| Isopoda | 2 | 0.79 | 2 | 1.36 |
| Crustaceae | 0 | 0 | 0 | 0 |
| Diplopoda | 10 | 3.95 | 10 | 6.80 |
| Orthoptera | 0 | 0 | 0 | 0 |
| Blattodea | 7 | 2.77 | 7 | 4.76 |
| Isoptera | 3 | 1.19 | 3 | 2.04 |
| Dermaptera | 3 | 1.19 | 3 | 2.04 |
| Homoptera | 3 | 1.19 | 3 | 2.04 |
| Heteroptera | 15 | 5.93 | 13 | 8.84 |
| Diptera | 10 | 3.95 | 10 | 6.80 |
| Lepidoptera | 6 | 2.37 | 6 | 4.08 |
| Coleoptera | 41 | 16.21 | 36 | 24.49 |
| Hymenoptera | 27 | 10.67 | 23 | 15.65 |
| Formicidae | 78 | 30.83 | 42 | 28.57 |
| Unidentif. Arthrop. | 4 | 1.58 | 4 | 2.72 |
| Larvae | 26 | 10.28 | 26 | 17.69 |
| *P. lilfordi* | 1 | 0.40 | 1 | 0.68 |
| Seeds | 1 | 0.40 | 1 | 0.68 |
| Carrion | 0 | 0 | 0 | 0 |
| Plant matter | 45.14 ± 3.6 |  | 86 | 58.50 |
| **Total** | **253** | **100** | **147** |  |
