# Supplementary material for: Omnivory of an Insular Lizard: Sources of Variation in the Diet of Podarcis lilfordi (Squamata, Lacertidae)
Source: PLoS One. 2016 Feb 12;11(2):e0148947. doi: 10.1371/journal.pone.0148947 (PMC4752353; doi:10.1371/journal.pone.0148947)
Supplement: S23 Table — (DOCX) [file pone.0148947.s031.docx]

| **Taxon** | **n** | **%n** | **presence** | **%presence** |
| --- | --- | --- | --- | --- |
| Gastropoda | 4 | 0.93 | 4 | 3.51 |
| Pseudoscorpionida | 0 | 0 | 0 | 0 |
| Araneae | 5 | 1.17 | 5 | 4.39 |
| Acarina | 1 | 0.23 | 1 | 0.88 |
| Isopoda | 0 | 0 | 0 | 0 |
| Crustaceae | 0 | 0 | 0 | 0 |
| Diplopoda | 0 | 0 | 0 | 0 |
| Orthoptera | 0 | 0 | 0 | 0 |
| Blattodea | 0 | 0 | 0 | 0 |
| Isoptera | 26 | 6.06 | 15 | 13.16 |
| Dermaptera | 0 | 0 | 0 | 0 |
| Homoptera | 1 | 0.23 | 1 | 0.88 |
| Heteroptera | 8 | 1.86 | 8 | 7.02 |
| Diptera | 0 | 0 | 0 | 0 |
| Lepidoptera | 2 | 0.47 | 2 | 1.75 |
| Coleoptera | 9 | 2.10 | 9 | 7.89 |
| Hymenoptera | 6 | 1.40 | 6 | 5.26 |
| Formicidae | 292 | 68.07 | 79 | 69.30 |
| Unidentif. Arthrop. | 2 | 0.47 | 2 | 1.75 |
| Larvae | 0 | 0 | 0 | 0 |
| *P. lilfordi* | 2 | 0.47 | 2 | 1.75 |
| Seeds | 71 | 16.55 | 65 | 57.02 |
| Carrion | 0 | 0 | 0 | 0 |
| Plant matter | 69.79 ± 4.08 |  | 86 | 75.44 |
| **Total** | **429** | **100** | **114** |  |
