# Supplementary material for: Omnivory of an Insular Lizard: Sources of Variation in the Diet of Podarcis lilfordi (Squamata, Lacertidae)
Source: PLoS One. 2016 Feb 12;11(2):e0148947. doi: 10.1371/journal.pone.0148947 (PMC4752353; doi:10.1371/journal.pone.0148947)
Supplement: S24 Table — (DOCX) [file pone.0148947.s032.docx]

| **Taxon** | **n** | **%n** | **presence** | **%presence** |
| --- | --- | --- | --- | --- |
| Gastropoda | 2 | 0.91 | 2 | 1.96 |
| Pseudoscorpionida | 0 | 0 | 0 | 0 |
| Araneae | 3 | 1.37 | 3 | 2.94 |
| Acarina | 0 | 0 | 0 | 0 |
| Isopoda | 9 | 4.11 | 9 | 8.82 |
| Crustaceae | 0 | 0 | 0 | 0 |
| Diplopoda | 1 | 0.46 | 1 | 0.98 |
| Orthoptera | 0 | 0 | 0 | 0 |
| Blattodea | 1 | 0.46 | 1 | 0.98 |
| Isoptera | 14 | 6.39 | 9 | 8.82 |
| Dermaptera | 1 | 0.46 | 1 | 0.98 |
| Homoptera | 50 | 22.83 | 32 | 31.37 |
| Heteroptera | 1 | 0.46 | 1 | 0.98 |
| Diptera | 7 | 3.20 | 7 | 6.86 |
| Lepidoptera | 1 | 0.46 | 1 | 0.98 |
| Coleoptera | 21 | 9.59 | 20 | 19.61 |
| Hymenoptera | 20 | 9.13 | 13 | 12.75 |
| Formicidae | 29 | 13.24 | 17 | 16.67 |
| Unidentif. Arthrop. | 0 | 0 | 0 | 0 |
| Larvae | 1 | 0.46 | 1 | 0.98 |
| *P. lilfordi* | 0 | 0 | 0 | 0 |
| Seeds | 58 | 26.48 | 54 | 52.94 |
| Carrion | 0 | 0 | 0 | 0 |
| Plant matter | 40.9 ± 4.66 |  | 49 | 48.04 |
| **Total** | **219** | **100** | **102** |  |
