# Supplementary material for: Omnivory of an Insular Lizard: Sources of Variation in the Diet of Podarcis lilfordi (Squamata, Lacertidae)
Source: PLoS One. 2016 Feb 12;11(2):e0148947. doi: 10.1371/journal.pone.0148947 (PMC4752353; doi:10.1371/journal.pone.0148947)
Supplement: S25 Table — (DOCX) [file pone.0148947.s033.docx]

| **Taxon** | **n** | **%n** | **presence** | **%presence** |
| --- | --- | --- | --- | --- |
| Gastropoda | 4 | 0.87 | 4 | 5.33 |
| Pseudoscorpionida | 1 | 0.22 | 1 | 1.33 |
| Araneae | 10 | 2.18 | 10 | 13.33 |
| Acarina | 0 | 0 | 0 | 0 |
| Isopoda | 4 | 0.87 | 4 | 5.33 |
| Crustaceae | 0 | 0 | 0 | 0 |
| Diplopoda | 2 | 0.44 | 2 | 2.67 |
| Orthoptera | 0 | 0 | 0 | 0 |
| Blattodea | 1 | 0.22 | 1 | 1.33 |
| Isoptera | 13 | 2.83 | 12 | 16.00 |
| Dermaptera | 0 | 0 | 0 | 0 |
| Homoptera | 7 | 1.53 | 6 | 8.00 |
| Heteroptera | 6 | 1.31 | 6 | 8.00 |
| Diptera | 2 | 0.44 | 2 | 2.67 |
| Lepidoptera | 3 | 0.65 | 3 | 4.00 |
| Coleoptera | 51 | 11.11 | 35 | 46.67 |
| Hymenoptera | 1 | 0.22 | 1 | 1.33 |
| Formicidae | 317 | 69.06 | 52 | 69.33 |
| Unidentif. Arthrop. | 1 | 0.22 | 1 | 1.33 |
| Larvae | 10 | 2.18 | 9 | 12.00 |
| *P. lilfordi* | 1 | 0.22 | 1 | 1.33 |
| Seeds | 24 | 5.23 | 21 | 28.00 |
| Carrion | 1 | 0.22 | 1 | 1.33 |
| Plant matter | 37.69 ± 4.98 |  | 42 | 56.00 |
| **Total** | **459** | **100** | **75** |  |
