# Supplementary material for: Omnivory of an Insular Lizard: Sources of Variation in the Diet of Podarcis lilfordi (Squamata, Lacertidae)
Source: PLoS One. 2016 Feb 12;11(2):e0148947. doi: 10.1371/journal.pone.0148947 (PMC4752353; doi:10.1371/journal.pone.0148947)
Supplement: S26 Table — (DOCX) [file pone.0148947.s034.docx]

| **Taxon** | **n** | **%n** | **presence** | **%presence** |
| --- | --- | --- | --- | --- |
| Gastropoda | 0 | 0 | 0 | 0 |
| Pseudoscorpionida | 3 | 1.61 | 3 | 4.76 |
| Araneae | 3 | 1.61 | 3 | 4.76 |
| Acarina | 0 | 0 | 0 | 0 |
| Isopoda | 3 | 1.61 | 3 | 4.76 |
| Crustaceae | 0 | 0 | 0 | 0 |
| Diplopoda | 1 | 0.54 | 1 | 1.59 |
| Orthoptera | 0 | 0 | 0 | 0 |
| Blattodea | 0 | 0 | 0 | 0 |
| Isoptera | 1 | 0.54 | 1 | 1.59 |
| Dermaptera | 0 | 0 | 0 | 0 |
| Homoptera | 19 | 10.22 | 12 | 19.05 |
| Heteroptera | 2 | 1.08 | 2 | 3.17 |
| Diptera | 0 | 0 | 0 | 0 |
| Lepidoptera | 1 | 0.54 | 1 | 1.59 |
| Coleoptera | 14 | 7.53 | 12 | 19.05 |
| Hymenoptera | 1 | 0.54 | 1 | 1.59 |
| Formicidae | 98 | 52.69 | 32 | 50.79 |
| Unidentif. Arthrop. | 2 | 1.08 | 2 | 3.17 |
| Larvae | 3 | 1.61 | 3 | 4.76 |
| *P. lilfordi* | 0 | 0 | 0 | 0 |
| Seeds | 35 | 18.82 | 26 | 41.27 |
| Carrion | 0 | 0 | 0 | 0 |
| Plant matter | 63.65 ± 5.64 |  | 49 | 77.78 |
| **Total** | **186** | **100** | **63** |  |
