# Supplementary material for: Omnivory of an Insular Lizard: Sources of Variation in the Diet of Podarcis lilfordi (Squamata, Lacertidae)
Source: PLoS One. 2016 Feb 12;11(2):e0148947. doi: 10.1371/journal.pone.0148947 (PMC4752353; doi:10.1371/journal.pone.0148947)
Supplement: S27 Table — (DOCX) [file pone.0148947.s035.docx]

| **Taxon** | **n** | **%n** | **presence** | **%presence** |
| --- | --- | --- | --- | --- |
| Gastropoda | 12 | 6.19 | 12 | 22.22 |
| Pseudoscorpionida | 1 | 0.52 | 1 | 1.85 |
| Araneae | 3 | 1.55 | 3 | 5.56 |
| Acarina | 0 | 0 | 0 | 0 |
| Isopoda | 2 | 1.03 | 2 | 3.70 |
| Crustaceae | 0 | 0 | 0 | 0 |
| Diplopoda | 2 | 1.03 | 2 | 3.70 |
| Orthoptera | 0 | 0 | 0 | 0 |
| Blattodea | 3 | 1.55 | 3 | 5.56 |
| Isoptera | 3 | 1.55 | 3 | 5.56 |
| Dermaptera | 4 | 2.06 | 4 | 7.41 |
| Homoptera | 24 | 12.37 | 17 | 31.48 |
| Heteroptera | 5 | 2.58 | 5 | 9.26 |
| Diptera | 1 | 0.52 | 1 | 1.85 |
| Lepidoptera | 2 | 1.03 | 2 | 3.70 |
| Coleoptera | 19 | 9.79 | 14 | 25.93 |
| Hymenoptera | 28 | 14.43 | 8 | 14.81 |
| Formicidae | 73 | 37.63 | 27 | 50.00 |
| Unidentif. Arthrop. | 0 | 0 | 0 | 0 |
| Larvae | 3 | 1.55 | 3 | 5.56 |
| *P. lilfordi* | 1 | 0.52 | 1 | 1.85 |
| Seeds | 7 | 3.61 | 6 | 11.11 |
| Carrion | 1 | 0.52 | 1 | 1.85 |
| Plant matter | 28.26 ± 5.37 |  | 32 | 59.26 |
| **Total** | **194** | **100** | **54** |  |
