# Supplementary material for: Omnivory of an Insular Lizard: Sources of Variation in the Diet of Podarcis lilfordi (Squamata, Lacertidae)
Source: PLoS One. 2016 Feb 12;11(2):e0148947. doi: 10.1371/journal.pone.0148947 (PMC4752353; doi:10.1371/journal.pone.0148947)
Supplement: S28 Table — (DOCX) [file pone.0148947.s036.docx]

| **Taxon** | **n** | **%n** | **presence** | **%presence** |
| --- | --- | --- | --- | --- |
| Gastropoda | 0 | 0 | 0 | 0 |
| Pseudoscorpionida | 0 | 0 | 0 | 0 |
| Araneae | 0 | 0 | 0 | 0 |
| Acarina | 0 | 0 | 0 | 0 |
| Isopoda | 0 | 0 | 0 | 0 |
| Crustaceae | 0 | 0 | 0 | 0 |
| Diplopoda | 6 | 7.69 | 6 | 24 |
| Orthoptera | 0 | 0 | 0 | 0 |
| Blattodea | 2 | 2.56 | 2 | 8 |
| Isoptera | 3 | 3.85 | 2 | 8 |
| Dermaptera | 0 | 0 | 0 | 0 |
| Homoptera | 0 | 0 | 0 | 0 |
| Heteroptera | 5 | 6.41 | 4 | 16 |
| Diptera | 1 | 1.28 | 1 | 4 |
| Lepidoptera | 0 | 0 | 0 | 0 |
| Coleoptera | 5 | 6.41 | 5 | 20 |
| Hymenoptera | 0 | 0 | 0 | 0 |
| Formicidae | 48 | 61.54 | 16 | 64 |
| Unidentif. Arthrop. | 1 | 1.28 | 1 | 4 |
| Larvae | 0 | 0 | 0 | 0 |
| *P. lilfordi* | 0 | 0 | 0 | 0 |
| Seeds | 7 | 5.97 | 6 | 24 |
| Carrion | 0 | 0 | 0 | 0 |
| Plant matter | 48 ± 8.43 |  | 21 | 84 |
| **Total** | **78** | **100** | **25** |  |
