# Supplementary material for: Omnivory of an Insular Lizard: Sources of Variation in the Diet of Podarcis lilfordi (Squamata, Lacertidae)
Source: PLoS One. 2016 Feb 12;11(2):e0148947. doi: 10.1371/journal.pone.0148947 (PMC4752353; doi:10.1371/journal.pone.0148947)
Supplement: S29 Table — (DOCX) [file pone.0148947.s037.docx]

| **Taxon** | **n** | **%n** | **presence** | **%presence** |
| --- | --- | --- | --- | --- |
| Gastropoda | 1 | 0.64 | 1 | 2.5 |
| Pseudoscorpionida | 0 | 0 | 0 | 0.0 |
| Araneae | 6 | 3.87 | 6 | 15.0 |
| Acarina | 0 | 0 | 0 | 0.0 |
| Isopoda | 9 | 5.81 | 9 | 22.5 |
| Crustaceae | 0 | 0 | 0 | 0.0 |
| Diplopoda | 0 | 0 | 0 | 0.0 |
| Orthoptera | 0 | 0 | 0 | 0.0 |
| Blattodea | 1 | 0.64 | 1 | 2.5 |
| Isoptera | 0 | 0 | 0 | 0.0 |
| Dermaptera | 0 | 0 | 0 | 0.0 |
| Homoptera | 20 | 2.90 | 12 | 30.0 |
| Heteroptera | 4 | 2.58 | 4 | 10.0 |
| Diptera | 5 | 3.26 | 5 | 12.5 |
| Lepidoptera | 2 | 1.29 | 2 | 5.0 |
| Coleoptera | 20 | 12.90 | 15 | 37.5 |
| Hymenoptera | 3 | 1.93 | 2 | 5.0 |
| Formicidae | 70 | 45.16 | 28 | 70.0 |
| Unidentif. Arthrop. | 7 | 4.52 | 6 | 15.0 |
| Larvae | 7 | 4.52 | 7 | 17.5 |
| *P. lilfordi* | 0 | 0 | 0 | 0.0 |
| Seeds | 0 | 0 | 0 | 0.0 |
| Carrion | 0 | 0 | 0 | 0.0 |
| Plant matter | 52.63 ± 6.53 |  | 38 | 95.0 |
| **Total** | **155** | **100** | **40** |  |
