# Supplementary material for: Omnivory of an Insular Lizard: Sources of Variation in the Diet of Podarcis lilfordi (Squamata, Lacertidae)
Source: PLoS One. 2016 Feb 12;11(2):e0148947. doi: 10.1371/journal.pone.0148947 (PMC4752353; doi:10.1371/journal.pone.0148947)
Supplement: S30 Table — (DOCX) [file pone.0148947.s038.docx]

| **Taxon** | **n** | **%n** | **presence** | **%presence** |
| --- | --- | --- | --- | --- |
| Gastropoda | 0 | 0 | 0 | 0 |
| Pseudoscorpionida | 1 | 4 | 1 | 25 |
| Araneae | 1 | 4 | 1 | 25 |
| Acarina | 0 | 0 | 0 | 0 |
| Isopoda | 1 | 4 | 1 | 25 |
| Crustaceae | 0 | 0 | 0 | 0 |
| Diplopoda | 0 | 0 | 0 | 0 |
| Orthoptera | 0 | 0 | 0 | 0 |
| Blattodea | 1 | 4 | 1 | 25 |
| Isoptera | 0 | 0 | 0 | 0 |
| Dermaptera | 0 | 0 | 0 | 0 |
| Homoptera | 2 | 8 | 2 | 50 |
| Heteroptera | 1 | 4 | 1 | 25 |
| Diptera | 2 | 8 | 2 | 50 |
| Lepidoptera | 0 | 0 | 0 | 0 |
| Coleoptera | 3 | 12 | 3 | 75 |
| Hymenoptera | 1 | 4 | 1 | 25 |
| Formicidae | 12 | 48 | 3 | 75 |
| Unidentif. Arthrop. | 0 | 0 | 0 | 0 |
| Larvae | 0 | 0 | 0 | 0 |
| *P. lilfordi* | 0 | 0 | 0 | 0 |
| Seeds | 0 | 0 | 0 | 0 |
| Carrion | 0 | 0 | 0 | 0 |
| Plant matter | 25 ± 6.45 |  | 4 | 100 |
| **Total** | **25** | **100** | **4** |  |
