# Supplementary material for: Omnivory of an Insular Lizard: Sources of Variation in the Diet of Podarcis lilfordi (Squamata, Lacertidae)
Source: PLoS One. 2016 Feb 12;11(2):e0148947. doi: 10.1371/journal.pone.0148947 (PMC4752353; doi:10.1371/journal.pone.0148947)
Supplement: S31 Table — (DOCX) [file pone.0148947.s039.docx]

| **Taxon** | **n** | **%n** | **presence** | **%presence** |
| --- | --- | --- | --- | --- |
| Gastropoda | 1 | 0.21 | 1 | 1.89 |
| Pseudoscorpionida | 0 | 0 | 0 | 0.00 |
| Araneae | 3 | 0.63 | 3 | 5.66 |
| Acarina | 0 | 0 | 0 | 0.00 |
| Isopoda | 3 | 0.63 | 3 | 5.66 |
| Crustaceae | 0 | 0 | 0 | 0 |
| Diplopoda | 0 | 0 | 0 | 0 |
| Orthoptera | 0 | 0 | 0 | 0 |
| Blattodea | 0 | 0 | 0 | 0 |
| Isoptera | 12 | 2.53 | 9 | 16.98 |
| Dermaptera | 0 | 0 | 0 | 0 |
| Homoptera | 0 | 0 | 0 | 0 |
| Heteroptera | 6 | 1.27 | 6 | 11.32 |
| Diptera | 0 | 0 | 0 | 0 |
| Lepidoptera | 0 | 0 | 0 | 0 |
| Coleoptera | 15 | 3.16 | 14 | 26.42 |
| Hymenoptera | 0 | 0 | 0 | 0 |
| Formicidae | 432 | 91.14 | 47 | 88.68 |
| Unidentif. Arthrop. | 1 | 0.21 | 1 | 1.89 |
| Larvae | 0 | 0 | 0 | 0 |
| *P. lilfordi* | 0 | 0 | 0 | 0 |
| Seeds | 1 | 0.21 | 1 | 1.89 |
| Carrion | 0 | 0 | 0 | 0 |
| Plant matter | 71.51 ± 5.09 |  | 46 | 86.79 |
| **Total** | **474** | **100** | **53** |  |
