# Supplementary material for: Omnivory of an Insular Lizard: Sources of Variation in the Diet of Podarcis lilfordi (Squamata, Lacertidae)
Source: PLoS One. 2016 Feb 12;11(2):e0148947. doi: 10.1371/journal.pone.0148947 (PMC4752353; doi:10.1371/journal.pone.0148947)
Supplement: S32 Table — (DOCX) [file pone.0148947.s040.docx]

| **Taxon** | **n** | **%n** | **presence** | **%presence** |
| --- | --- | --- | --- | --- |
| Gastropoda | 0 | 0 | 0 | 0 |
| Pseudoscorpionida | 0 | 0 | 0 | 0 |
| Araneae | 2 | 0.71 | 2 | 3.77 |
| Acarina | 0 | 0 | 0 | 0 |
| Isopoda | 4 | 1.41 | 4 | 7.55 |
| Crustaceae | 0 | 0 | 0 | 0 |
| Diplopoda | 0 | 0 | 0 | 0 |
| Orthoptera | 0 | 0 | 0 | 0 |
| Blattodea | 1 | 0.35 | 1 | 1.89 |
| Isoptera | 7 | 2.47 | 6 | 11.32 |
| Dermaptera | 0 | 0 | 0 | 0 |
| Homoptera | 0 | 0 | 0 | 0 |
| Heteroptera | 6 | 2.12 | 6 | 11.32 |
| Diptera | 6 | 2.12 | 6 | 11.32 |
| Lepidoptera | 1 | 0.35 | 1 | 1.89 |
| Coleoptera | 13 | 4.59 | 12 | 22.64 |
| Hymenoptera | 0 | 0 | 0 | 0 |
| Formicidae | 238 | 84.1 | 39 | 73.58 |
| Unidentif. Arthrop. | 0 | 0 | 0 | 0 |
| Larvae | 2 | 0.71 | 2 | 3.77 |
| *P. lilfordi* | 0 | 0 | 0 | 0 |
| Seeds | 1 | 0.35 | 1 | 1.89 |
| Carrion | 2 | 0.71 | 2 | 3.77 |
| Plant matter | 40.89 ± 5.98 |  | 26 | 49.06 |
| **Total** | **283** | **100** | **53** |  |
