# Supplementary material for: Omnivory of an Insular Lizard: Sources of Variation in the Diet of Podarcis lilfordi (Squamata, Lacertidae)
Source: PLoS One. 2016 Feb 12;11(2):e0148947. doi: 10.1371/journal.pone.0148947 (PMC4752353; doi:10.1371/journal.pone.0148947)
Supplement: S33 Table — (DOCX) [file pone.0148947.s041.docx]

| **Taxon** | **n** | **%n** | **presence** | **%presence** |
| --- | --- | --- | --- | --- |
| Gastropoda | 3 | 1.09 | 3 | 5.45 |
| Pseudoscorpionida | 0 | 0 | 0 | 0 |
| Araneae | 3 | 1.09 | 3 | 5.45 |
| Acarina | 0 | 0 | 0 | 0 |
| Isopoda | 7 | 2.54 | 7 | 12.73 |
| Crustaceae | 0 | 0 | 0 | 0 |
| Diplopoda | 0 | 0 | 0 | 0 |
| Orthoptera | 0 | 0 | 0 | 0 |
| Blattodea | 0 | 0 | 0 | 0 |
| Isoptera | 15 | 5.45 | 11 | 20.00 |
| Dermaptera | 0 | 0 | 0 | 0 |
| Homoptera | 10 | 3.64 | 10 | 18.19 |
| Heteroptera | 7 | 2.54 | 7 | 12.73 |
| Diptera | 3 | 1.09 | 3 | 5.45 |
| Lepidoptera | 0 | 0 | 0 | 0 |
| Coleoptera | 17 | 6.18 | 17 | 30.91 |
| Hymenoptera | 6 | 2.18 | 6 | 10.91 |
| Formicidae | 199 | 72.36 | 38 | 69.09 |
| Unidentif. Arthrop. | 0 | 0 | 0 | 0 |
| Larvae | 3 | 1.09 | 3 | 5.45 |
| *P. lilfordi* | 0 | 0 | 0 | 0 |
| Seeds | 2 | 0.72 | 2 | 3.64 |
| Carrion | 0 | 0 | 0 | 0 |
| Plant matter | 41.87 ± 5.47 |  | 33 | 60.00 |
| **Total** | **275** | **100** | **55** |  |
