# Supplementary material for: Omnivory of an Insular Lizard: Sources of Variation in the Diet of Podarcis lilfordi (Squamata, Lacertidae)
Source: PLoS One. 2016 Feb 12;11(2):e0148947. doi: 10.1371/journal.pone.0148947 (PMC4752353; doi:10.1371/journal.pone.0148947)
Supplement: S34 Table — (DOCX) [file pone.0148947.s042.docx]

| **Taxon** | **n** | **%n** | **presence** | **%presence** |
| --- | --- | --- | --- | --- |
| Gastropoda | 1 | 0.28 | 1 | 1.72 |
| Pseudoscorpionida | 1 | 0.28 | 1 | 1.72 |
| Araneae | 2 | 0.56 | 2 | 3.45 |
| Acarina | 0 | 0 | 0 | 0 |
| Isopoda | 2 | 0.56 | 2 | 3.45 |
| Crustaceae | 0 | 0 | 0 | 0 |
| Diplopoda | 3 | 0.84 | 3 | 5.17 |
| Orthoptera | 0 | 0 | 0 | 0 |
| Blattodea | 0 | 0 | 0 | 0 |
| Isoptera | 8 | 2.23 | 5 | 8.62 |
| Dermaptera | 0 | 0 | 0 | 0 |
| Homoptera | 16 | 4.46 | 12 | 20.69 |
| Heteroptera | 8 | 2.23 | 7 | 12.07 |
| Diptera | 0 | 0 | 0 | 0 |
| Lepidoptera | 0 | 0 | 0 | 0 |
| Coleoptera | 20 | 5.57 | 15 | 25.86 |
| Hymenoptera | 5 | 1.39 | 5 | 8.62 |
| Formicidae | 280 | 78.00 | 48 | 82.76 |
| Unidentif. Arthrop. | 1 | 0.28 | 1 | 1.72 |
| Larvae | 3 | 0.84 | 3 | 5.17 |
| *P. lilfordi* | 0 | 0.00 | 0 | 0 |
| Seeds | 9 | 2.51 | 7 | 12.07 |
| Carrion | 0 | 0 | 0 | 0 |
| Plant matter | 30.97 ± 4.86 |  | 36 | 62.07 |
| **Total** | **359** | **100** | **58** |  |
