# Supplementary material for: Omnivory of an Insular Lizard: Sources of Variation in the Diet of Podarcis lilfordi (Squamata, Lacertidae)
Source: PLoS One. 2016 Feb 12;11(2):e0148947. doi: 10.1371/journal.pone.0148947 (PMC4752353; doi:10.1371/journal.pone.0148947)
Supplement: S35 Table — (DOCX) [file pone.0148947.s043.docx]

| **Taxon** | **n** | **%n** | **presence** | **%presence** |
| --- | --- | --- | --- | --- |
| Gastropoda | 2 | 1.77 | 2 | 9.52 |
| Pseudoscorpionida | 0 | 0 | 0 | 0 |
| Araneae | 1 | 0.88 | 1 | 4.76 |
| Acarina | 0 | 0 | 0 | 0 |
| Isopoda | 1 | 0.88 | 1 | 4.76 |
| Crustaceae | 0 | 0 | 0 | 0 |
| Diplopoda | 0 | 0 | 0 | 0 |
| Orthoptera | 0 | 0 | 0 | 0 |
| Blattodea | 0 | 0 | 0 | 0 |
| Isoptera | 1 | 0.88 | 1 | 4.76 |
| Dermaptera | 0 | 0 | 0 | 0 |
| Homoptera | 0 | 0 | 0 | 0 |
| Heteroptera | 5 | 4.42 | 5 | 23.81 |
| Diptera | 0 | 0 | 0 | 0 |
| Lepidoptera | 0 | 0 | 0 | 0 |
| Coleoptera | 9 | 7.96 | 7 | 33.33 |
| Hymenoptera | 3 | 2.65 | 3 | 14.29 |
| Formicidae | 89 | 78.76 | 18 | 85.71 |
| Unidentif. Arthrop. | 0 | 0 | 0 | 0 |
| Larvae | 2 | 1.77 | 2 | 9.52 |
| *P. lilfordi* | 0 | 0 | 0 | 0 |
| Seeds | 0 | 0 | 0 | 0 |
| Carrion | 0 | 0 | 0 | 0 |
| Plant matter | 45.67 ± 10 |  | 17 | 8.96 |
| **Total** | **113** | **100** | **21** |  |
