# Supplementary material for: Omnivory of an Insular Lizard: Sources of Variation in the Diet of Podarcis lilfordi (Squamata, Lacertidae)
Source: PLoS One. 2016 Feb 12;11(2):e0148947. doi: 10.1371/journal.pone.0148947 (PMC4752353; doi:10.1371/journal.pone.0148947)
Supplement: S36 Table — (DOCX) [file pone.0148947.s044.docx]

| **Taxon** | **n** | **%n** | **presence** | **%presence** |
| --- | --- | --- | --- | --- |
| Gastropoda | 0 | 0 | 0 | 0 |
| Pseudoscorpionida | 0 | 0 | 0 | 0 |
| Araneae | 0 | 0 | 0 | 0 |
| Acarina | 0 | 0 | 0 | 0 |
| Isopoda | 3 | 2.07 | 3 | 11.11 |
| Crustaceae | 0 | 0 | 0 | 0 |
| Diplopoda | 1 | 0.69 | 1 | 3.70 |
| Orthoptera | 0 | 0 | 0 | 0 |
| Blattodea | 0 | 0 | 0 | 0 |
| Isoptera | 3 | 2.07 | 2 | 7.41 |
| Dermaptera | 0 | 0 | 0 | 0 |
| Homoptera | 1 | 0.69 | 1 | 3.70 |
| Heteroptera | 8 | 5.52 | 8 | 29.63 |
| Diptera | 0 | 0 | 0 | 0 |
| Lepidoptera | 0 | 0 | 0 | 0 |
| Coleoptera | 10 | 6.90 | 10 | 37.04 |
| Hymenoptera | 1 | 0.69 | 1 | 3.70 |
| Formicidae | 117 | 80.70 | 21 | 77.78 |
| Unidentif. Arthrop. | 0 | 0 | 0 | 0 |
| Larvae | 1 | 0.70 | 1 | 3.70 |
| *P. lilfordi* | 0 | 0 | 0 | 0 |
| Seeds | 0 | 0 | 0 | 0 |
| Carrion | 0 | 0 | 0 | 0 |
| Plant matter | 43.11 ± 7.59 |  | 21 | 77.78 |
| **Total** | **145** | **100** | **27** |  |
