# Supplementary material for: Omnivory of an Insular Lizard: Sources of Variation in the Diet of Podarcis lilfordi (Squamata, Lacertidae)
Source: PLoS One. 2016 Feb 12;11(2):e0148947. doi: 10.1371/journal.pone.0148947 (PMC4752353; doi:10.1371/journal.pone.0148947)
Supplement: S37 Table — (DOCX) [file pone.0148947.s045.docx]

| **Taxon** | **n** | **%n** | **presence** | **%presence** |
| --- | --- | --- | --- | --- |
| Gastropoda | 1 | 0.8 | 1 | 7.69 |
| Pseudoscorpionida | 0 | 0 | 0 | 0 |
| Araneae | 2 | 1.6 | 2 | 15.38 |
| Acarina | 0 | 0 | 0 | 0 |
| Isopoda | 1 | 0.8 | 1 | 7.69 |
| Crustaceae | 0 | 0 | 0 | 0 |
| Diplopoda | 0 | 0 | 0 | 0 |
| Orthoptera | 0 | 0 | 0 | 0 |
| Blattodea | 0 | 0 | 0 | 0 |
| Isoptera | 0 | 0 | 0 | 0 |
| Dermaptera | 0 | 0 | 0 | 0 |
| Homoptera | 0 | 0 | 0 | 0 |
| Heteroptera | 0 | 0 | 0 | 0 |
| Diptera | 3 | 2.4 | 2 | 15.38 |
| Lepidoptera | 2 | 1.6 | 2 | 15.38 |
| Coleoptera | 3 | 2.4 | 3 | 23.08 |
| Hymenoptera | 102 | 81.6 | 1 | 7.69 |
| Formicidae | 9 | 7.2 | 5 | 38.46 |
| Unidentif. Arthrop. | 1 | 0.8 | 1 | 7.69 |
| Larvae | 1 | 0.8 | 1 | 7.69 |
| *P. lilfordi* | 0 | 0 | 0 | 0 |
| Seeds | 0 | 0 | 0 | 0 |
| Carrion | 0 | 0 | 0 | 0 |
| Plant matter | 23.08 ± 10.02 |  | 6 | 26.15 |
| **Total** | **125** | **100** | **13** |  |
