# Supplementary material for: Omnivory of an Insular Lizard: Sources of Variation in the Diet of Podarcis lilfordi (Squamata, Lacertidae)
Source: PLoS One. 2016 Feb 12;11(2):e0148947. doi: 10.1371/journal.pone.0148947 (PMC4752353; doi:10.1371/journal.pone.0148947)
Supplement: S38 Table — (DOCX) [file pone.0148947.s046.docx]

| **Taxon** | **n** | **%n** | **presence** | **%presence** |
| --- | --- | --- | --- | --- |
| Gastropoda | 2 | 0.89 | 2 | 6.06 |
| Pseudoscorpionida | 5 | 2.23 | 5 | 15.15 |
| Araneae | 9 | 4.02 | 8 | 24.24 |
| Acarina | 2 | 0.89 | 1 | 3.03 |
| Isopoda | 1 | 0.45 | 1 | 3.03 |
| Crustaceae | 0 | 0 | 0 | 0 |
| Diplopoda | 5 | 2.23 | 5 | 15.15 |
| Orthoptera | 0 | 0 | 0 | 0 |
| Blattodea | 3 | 1.34 | 3 | 9.09 |
| Isoptera | 0 | 0 | 0 | 0 |
| Dermaptera | 4 | 1.79 | 3 | 9.09 |
| Homoptera | 14 | 6.25 | 14 | 42.42 |
| Heteroptera | 5 | 2.23 | 4 | 12.12 |
| Diptera | 7 | 3.13 | 7 | 21.21 |
| Lepidoptera | 2 | 0.89 | 1 | 3.03 |
| Coleoptera | 21 | 9.38 | 17 | 51.52 |
| Hymenoptera | 12 | 5.36 | 2 | 6.06 |
| Formicidae | 121 | 54.02 | 23 | 69.70 |
| Unidentif. Arthrop. | 1 | 0.45 | 1 | 3.03 |
| Larvae | 1 | 0.45 | 1 | 3.03 |
| *P. lilfordi* | 3 | 1.34 | 3 | 9.09 |
| Seeds | 0 | 0 | 0 | 0 |
| Carrion | 6 | 2.68 | 6 | 18.18 |
| Plant matter | 50.67 ± 7.77 |  | 33 | 100 |
| **Total** | **224** | **100** | **33** |  |
