# Supplementary material for: Omnivory of an Insular Lizard: Sources of Variation in the Diet of Podarcis lilfordi (Squamata, Lacertidae)
Source: PLoS One. 2016 Feb 12;11(2):e0148947. doi: 10.1371/journal.pone.0148947 (PMC4752353; doi:10.1371/journal.pone.0148947)
Supplement: S39 Table — (DOCX) [file pone.0148947.s047.docx]

| **Taxon** | **n** | **%n** | **presence** | **%presence** |
| --- | --- | --- | --- | --- |
| Gastropoda | 0 | 0 | 0 | 0 |
| Pseudoscorpionida | 1 | 0.52 | 1 | 6.25 |
| Araneae | 1 | 0.52 | 1 | 6.25 |
| Acarina | 125 | 64.77 | 2 | 12.50 |
| Isopoda | 1 | 0.52 | 1 | 6.25 |
| Crustaceae | 0 | 0 | 0 | 0 |
| Diplopoda | 1 | 0.52 | 1 | 6.25 |
| Orthoptera | 0 | 0 | 0 | 0 |
| Blattodea | 0 | 0 | 0 | 0 |
| Isoptera | 0 | 0 | 0 | 0 |
| Dermaptera | 4 | 2.07 | 4 | 25.0 |
| Homoptera | 6 | 3.11 | 3 | 18.75 |
| Heteroptera | 0 | 0 | 0 | 0 |
| Diptera | 0 | 0 | 0 | 0 |
| Lepidoptera | 0 | 0 | 0 | 0 |
| Coleoptera | 7 | 3.63 | 6 | 37.50 |
| Hymenoptera | 0 | 0 | 0 | 0 |
| Formicidae | 45 | 23.32 | 11 | 68.75 |
| Unidentif. Arthrop. | 1 | 0.52 | 1 | 6.25 |
| Larvae | 0 | 0 | 0 | 0 |
| *P. lilfordi* | 1 | 0.52 | 1 | 6.25 |
| Seeds | 0 | 0 | 0 | 0 |
| Carrion | 0 | 0 | 0 | 0 |
| Plant matter | 29.44 ± 8.29 |  | 14 | 87.50 |
| **Total** | **193** | **100** | **16** |  |
