# Supplementary material for: Omnivory of an Insular Lizard: Sources of Variation in the Diet of Podarcis lilfordi (Squamata, Lacertidae)
Source: PLoS One. 2016 Feb 12;11(2):e0148947. doi: 10.1371/journal.pone.0148947 (PMC4752353; doi:10.1371/journal.pone.0148947)
Supplement: S40 Table — (DOCX) [file pone.0148947.s048.docx]

| **Taxon** | **n** | **%n** | **presence** | **%presence** |
| --- | --- | --- | --- | --- |
| Gastropoda | 2 | 0.44 | 2 | 2.33 |
| Pseudoscorpionida | 6 | 1.33 | 6 | 6.98 |
| Araneae | 6 | 1.33 | 6 | 6.98 |
| Acarina | 0 | 0 | 0 | 0 |
| Isopoda | 6 | 1.33 | 6 | 6.98 |
| Crustaceae | 0 | 0 | 0 | 0 |
| Diplopoda | 17 | 3.77 | 17 | 19.77 |
| Orthoptera | 0 | 0 | 0 | 0 |
| Blattodea | 0 | 0 | 0 | 0 |
| Isoptera | 19 | 4.21 | 12 | 13.95 |
| Dermaptera | 1 | 0.22 | 1 | 1.16 |
| Homoptera | 1 | 0.22 | 1 | 1.16 |
| Heteroptera | 15 | 3.33 | 11 | 12.79 |
| Diptera | 7 | 1.55 | 7 | 8.14 |
| Lepidoptera | 2 | 1.44 | 2 | 2.33 |
| Coleoptera | 36 | 7.98 | 32 | 37.21 |
| Hymenoptera | 6 | 1.33 | 3 | 3.49 |
| Formicidae | 324 | 71.84 | 70 | 81.40 |
| Unidentif. Arthrop. | 2 | 0.44 | 2 | 2.33 |
| Larvae | 0 | 0 | 0 | 0 |
| *P. lilfordi* | 1 | 0.22 | 1 | 1.16 |
| Seeds | 0 | 0 | 0 | 0 |
| Carrion | 0 | 0 | 0 | 0 |
| Plant matter | 57.94 ± 4.09 |  | 70 | 81.40 |
| **Total** | **451** | **100** | **86** |  |
