# Supplementary material for: Omnivory of an Insular Lizard: Sources of Variation in the Diet of Podarcis lilfordi (Squamata, Lacertidae)
Source: PLoS One. 2016 Feb 12;11(2):e0148947. doi: 10.1371/journal.pone.0148947 (PMC4752353; doi:10.1371/journal.pone.0148947)
Supplement: S41 Table — (DOCX) [file pone.0148947.s049.docx]

| **Taxon** | **n** | **%n** | **presence** | **%presence** |
| --- | --- | --- | --- | --- |
| Gastropoda | 1 | 0.32 | 1 | 1.72 |
| Pseudoscorpionida | 3 | 0.97 | 3 | 5.17 |
| Araneae | 6 | 1.95 | 6 | 10.34 |
| Acarina | 1 | 0.32 | 1 | 1.72 |
| Isopoda | 1 | 0.32 | 1 | 1.72 |
| Crustaceae | 0 | 0 | 0 | 0 |
| Diplopoda | 0 | 0 | 0 | 0 |
| Orthoptera | 0 | 0 | 0 | 0 |
| Blattodea | 0 | 0 | 0 | 0 |
| Isoptera | 12 | 3.90 | 10 | 17.24 |
| Dermaptera | 0 | 0 | 0 | 0 |
| Homoptera | 12 | 3.90 | 6 | 10.34 |
| Heteroptera | 26 | 8.44 | 20 | 34.48 |
| Diptera | 1 | 0.32 | 1 | 1.72 |
| Lepidoptera | 1 | 0.32 | 1 | 1.72 |
| Coleoptera | 19 | 6.17 | 18 | 31.03 |
| Hymenoptera | 0 | 0 | 0 | 0 |
| Formicidae | 213 | 69.16 | 45 | 77.59 |
| Unidentif. Arthrop. | 1 | 0.32 | 1 | 1.72 |
| Larvae | 7 | 2.27 | 7 | 12.07 |
| *P. lilfordi* | 3 | 0.97 | 3 | 5.17 |
| Seeds | 1 | 0.32 | 1 | 1.72 |
| Carrion | 0 | 0 | 0 | 0 |
| Plant matter | 36.66 ± 5.45 |  | 36 | 62.07 |
| **Total** | **308** | **100** | **58** |  |
