# Supplementary material for: Omnivory of an Insular Lizard: Sources of Variation in the Diet of Podarcis lilfordi (Squamata, Lacertidae)
Source: PLoS One. 2016 Feb 12;11(2):e0148947. doi: 10.1371/journal.pone.0148947 (PMC4752353; doi:10.1371/journal.pone.0148947)
Supplement: S42 Table — (DOCX) [file pone.0148947.s050.docx]

| **Taxon** | **n** | **%n** | **presence** | **%presence** |
| --- | --- | --- | --- | --- |
| Gastropoda | 2 | 0.45 | 2 | 3.77 |
| Pseudoscorpionida | 1 | 0.22 | 1 | 1.89 |
| Araneae | 3 | 0.67 | 3 | 5.66 |
| Acarina | 66 | 14.77 | 6 | 11.32 |
| Isopoda | 0 | 0 | 0 | 0 |
| Crustaceae | 0 | 0 | 0 | 0 |
| Diplopoda | 0 | 0 | 0 | 0 |
| Orthoptera | 0 | 0 | 0 | 0 |
| Blattodea | 1 | 0.22 | 1 | 1.89 |
| Isoptera | 0 | 0 | 0 | 0 |
| Dermaptera | 1 | 0.22 | 1 | 1.89 |
| Homoptera | 7 | 1.57 | 7 | 13.21 |
| Heteroptera | 7 | 1.57 | 7 | 13.21 |
| Diptera | 1 | 0.22 | 1 | 1.89 |
| Lepidoptera | 3 | 0.67 | 2 | 3.77 |
| Coleoptera | 20 | 4.47 | 19 | 35.85 |
| Hymenoptera | 0 | 0 | 0 | 0 |
| Formicidae | 329 | 73.60 | 47 | 88.68 |
| Unidentif. Arthrop. | 0 | 0 | 0 | 0 |
| Larvae | 4 | 0.89 | 4 | 7.55 |
| *P. lilfordi* | 1 | 0.22 | 1 | 1.89 |
| Seeds | 0 | 0 | 0 | 0 |
| Carrion | 1 | 0.22 | 1 | 1.89 |
| Plant matter | 45.85 ± 5.3 |  | 48 | 90.57 |
| **Total** | **447** | **100** | **53** |  |
