# Supplementary material for: Omnivory of an Insular Lizard: Sources of Variation in the Diet of Podarcis lilfordi (Squamata, Lacertidae)
Source: PLoS One. 2016 Feb 12;11(2):e0148947. doi: 10.1371/journal.pone.0148947 (PMC4752353; doi:10.1371/journal.pone.0148947)
Supplement: S43 Table — (DOCX) [file pone.0148947.s051.docx]

| **Taxon** | **n** | **%n** | **presence** | **%presence** |
| --- | --- | --- | --- | --- |
| Gastropoda | 1 | 0.49 | 1 | 3.85 |
| Pseudoscorpionida | 0 | 0 | 0 | 0 |
| Araneae | 3 | 1.46 | 3 | 11.54 |
| Acarina | 0 | 0 | 0 | 0 |
| Isopoda | 0 | 0 | 0 | 0 |
| Crustaceae | 0 | 0 | 0 | 0 |
| Diplopoda | 4 | 1.95 | 4 | 15.38 |
| Orthoptera | 1 | 0.49 | 1 | 3.85 |
| Blattodea | 0 | 0 | 0 | 0 |
| Isoptera | 1 | 0.49 | 1 | 3.85 |
| Dermaptera | 0 | 0 | 0 | 0 |
| Homoptera | 0 | 0 | 0 | 0 |
| Heteroptera | 37 | 18.05 | 19 | 73.08 |
| Diptera | 0 | 0 | 0 | 0 |
| Lepidoptera | 2 | 0.98 | 2 | 7.69 |
| Coleoptera | 9 | 4.39 | 8 | 30.77 |
| Hymenoptera | 1 | 0.49 | 1 | 3.85 |
| Formicidae | 141 | 68.78 | 21 | 80.77 |
| Unidentif. Arthrop. | 0 | 0 | 0 | 0 |
| Larvae | 1 | 0.49 | 1 | 3.85 |
| *P. lilfordi* | 2 | 0.98 | 2 | 7.69 |
| Seeds | 0 | 0 | 0 | 0 |
| Carrion | 2 | 0.98 | 2 | 7.69 |
| Plant matter | 16.19 ± 5.71 |  | 13 | 50 |
| **Total** | **205** | **100** | **26** |  |
