# Supplementary material for: Omnivory of an Insular Lizard: Sources of Variation in the Diet of Podarcis lilfordi (Squamata, Lacertidae)
Source: PLoS One. 2016 Feb 12;11(2):e0148947. doi: 10.1371/journal.pone.0148947 (PMC4752353; doi:10.1371/journal.pone.0148947)
Supplement: S44 Table — (DOCX) [file pone.0148947.s052.docx]

| **Taxon** | **%n**  **availability** | **%n diet** | **D** | **E** |
| --- | --- | --- | --- | --- |
| Gastropoda | 0 | 1.2048 | +1 | +1 |
| Pseudoscorpionida | 0 | 0.9036 | +1 | +1 |
| Araneae | 0 | 5.4217 | +1 | +1 |
| Acarina | 0 | 0 | -- | -- |
| Isopoda | 0 | 14.1566 | +1 | +1 |
| Crustaceae | 0 | 0 | -- | -- |
| Diplopoda | 0 | 3.9157 | +1 | +1 |
| Orthoptera | 5.4054 | 0 | +1 | -1 |
| Blattodea | 0 | 1.5060 | +1 | +1 |
| Isoptera | 0 | 1.8072 | +1 | +1 |
| Dermaptera | 0 | 0 | -- | -- |
| Homoptera | 0 | 1.8072 | +1 | +1 |
| Heteroptera | 9.4594 | 3.3132 | -0.5060 | -0.3049 |
| Diptera | 32.4324 | 2.4096 | -.9021 | -0.7970 |
| Lepidoptera | 6.7567 | 2.1084 | -0.5417 | -0.3574 |
| Coleoptera | 12.1622 | 9.3373 | -0.1469 | 0.0773 |
| Hymenoptera | 9.4594 | 13.2530 | 0.1877 | 0.3611 |
| Formicidae | 24.3243 | 25.3012 | 0.0262 | 0.2253 |
| Unidentif. Arthrop. | 0 | 3.0120 | +1 | +1 |
| Larvae | 0 | 6.6265 | +1 | +1 |
| *P. lilfordi* | 0 | 0 | -- | -- |
| Seeds | 0 | 3.9157 | +1 | +1 |
| Tysanura | 0 | 0 | -- | -- |
| Neuroptera | 0 | 0 | -- | -- |
| **Total** | **100** | **100** |  |  |

Table B44
