# Supplementary material for: Omnivory of an Insular Lizard: Sources of Variation in the Diet of Podarcis lilfordi (Squamata, Lacertidae)
Source: PLoS One. 2016 Feb 12;11(2):e0148947. doi: 10.1371/journal.pone.0148947 (PMC4752353; doi:10.1371/journal.pone.0148947)
Supplement: S45 Table — (DOCX) [file pone.0148947.s053.docx]

| **Taxon** | **%n**  **availability** | **%n diet** | **D** | **E** |
| --- | --- | --- | --- | --- |
| Gastropoda | 0 | 2.9925 | +1 | +1 |
| Pseudoscorpionida | 0 | 1.4962 | +1 | +1 |
| Araneae | 3.1250 | 5.9850 | 0.3274 | 0.1820 |
| Acarina | 0 | 0.7481 | +1 | +1 |
| Isopoda | 0 | 9.9750 | +1 | +1 |
| Crustaceae | 0 | 0 | -- | -- |
| Diplopoda | 0 | 0.7481 | +1 | +1 |
| Orthoptera | 2.0833 | 0 | -1 | -1 |
| Blattodea | 0 | 2.4938 | +1 | +1 |
| Isoptera | 0 | 0.7481 | +1 | +1 |
| Dermaptera | 0 | 0 | -- | -- |
| Homoptera | 5.2083 | 1.7456 | -0.5113 | -0.5963 |
| Heteroptera | 22.9167 | 3.4912 | -0.7830 | -0.7938 |
| Diptera | 16.6667 | 0.4987 | -0.9111 | -0.9558 |
| Lepidoptera | 2.0833 | 3.9900 | 0.3228 | 0.1820 |
| Coleoptera | 7.2917 | 15.7107 | 0.4065 | 0.2383 |
| Hymenoptera | 3.1250 | 21.1970 | 0.7858 | 0.6731 |
| Formicidae | 37.50 | 20.9476 | -0.3873 | -0.4070 |
| Unidentif. Arthrop. | 0 | 1.7456 | +1 | +1 |
| Larvae | 0 | 2.7431 | +1 | +1 |
| *P. lilfordi* | 0 | 0.7481 | +1 | +1 |
| Seeds | 0 | 1.9950 | +1 | +1 |
| Tysanura | 0 | 0 | -- | -- |
| Neuroptera | 0 | 0 | -- | -- |
| **Total** | **100** | **100** |  |  |

Table B45
