# Supplementary material for: Omnivory of an Insular Lizard: Sources of Variation in the Diet of Podarcis lilfordi (Squamata, Lacertidae)
Source: PLoS One. 2016 Feb 12;11(2):e0148947. doi: 10.1371/journal.pone.0148947 (PMC4752353; doi:10.1371/journal.pone.0148947)
Supplement: S47 Table — (DOCX) [file pone.0148947.s055.docx]

| **Taxon** | **%n**  **availability** | **%n**  **diet** | **D** | **E** |
| --- | --- | --- | --- | --- |
| Gastropoda | 1.8518 | 5.5363 | 0.5129 | 0.0638 |
| Pseudoscorpionida | 0 | 0 | -- | -- |
| Araneae | 0 | 3.4602 | +1 | +1 |
| Acarina | 1.8518 | 0 | -1 | -1 |
| Isopoda | 1.8518 | 28.7197 | 0.9105 | 0.7010 |
| Crustaceae | 0 | 0 | -- | -- |
| Diplopoda | 0 | 4.4983 | +1 | +1 |
| Orthoptera | 0 | 0 | -- | -- |
| Blattodea | 1.85018 | 1.0380 | -0.2854 | -0.6487 |
| Isoptera | 0 | 1.3840 | +1 | +1 |
| Dermaptera | 0 | 0 | -- | -- |
| Homoptera | 11.1111 | 1.7301 | -0.7531 | -0.8882 |
| Heteroptera | 0 | 4.1522 | +1 | +1 |
| Diptera | 5.5555 | 2.0761 | -0.4701 | -0.7513 |
| Lepidoptera | 0 | 3.8062 | +1 | +1 |
| Coleoptera | 0 | 7.9585 | +1 | +1 |
| Hymenoptera | 3.7037 | 4.1522 | 0.0594 | -0.4024 |
| Formicidae | 72.2222 | 24.5675 | -0.7773 | -0.7710 |
| Unidentif. Arthrop. | 0 | 2.7682 | +1 | +1 |
| Larvae | 0 | 1.0380 | +1 | +1 |
| *P. lilfordi* | 0 | 0 | -- | -- |
| Seeds | 0 | 3.1142 | +1 | +1 |
| Tysanura | 0 | 0 | -- | -- |
| Neuroptera | 0 | 0 | -- | -- |
| **Total** | **100** | **100** |  | **--** |

Table B47
