# Supplementary material for: Omnivory of an Insular Lizard: Sources of Variation in the Diet of Podarcis lilfordi (Squamata, Lacertidae)
Source: PLoS One. 2016 Feb 12;11(2):e0148947. doi: 10.1371/journal.pone.0148947 (PMC4752353; doi:10.1371/journal.pone.0148947)
Supplement: S48 Table — (DOCX) [file pone.0148947.s056.docx]

| **Taxon** | **%n**  **availability** | **%n diet** | **D** | **E** |
| --- | --- | --- | --- | --- |
| Gastropoda | 1.1765 | 2.8985 | 0.4298 | 0.2961 |
| Pseudoscorpionida | 0 | 0 | -- | -- |
| Araneae | 0 | 2.8985 | +1 | +1 |
| Acarina | 1.1765 | 0 | -1 | -1 |
| Isopoda | 0 | 4.3478 | +1 | +1 |
| Crustaceae | 0 | 0 | -- | -- |
| Diplopoda | 0 | 0 | -- | -- |
| Orthoptera | 2.3529 | 0 | -1 | -1 |
| Blattodea | 0 | 0 | -- | -- |
| Isoptera | 0 | 1.4493 | +1 | +1 |
| Dermaptera | 0 | 0 | -- | -- |
| Homoptera | 1.1765 | 1.4493 | 0.1053 | -0.0413 |
| Heteroptera | 2.3529 | 8.6956 | 0.5961 | 0.4683 |
| Diptera | 10.5882 | 0 | -1 | -1 |
| Lepidoptera | 2.3529 | 8.6956 | 0.5961 | 0.4683 |
| Coleoptera | 3.5294 | 4.3478 | 0.1081 | -0.0413 |
| Hymenoptera | 8.2353 | 1.4493 | -0.7184 | -0.7670 |
| Formicidae | 67.0588 | 59.4203 | -0.1632 | -0.2030 |
| Unidentif. Arthrop. | 0 | 0 | -- | -- |
| Larvae | 0 | 4.3448 | +1 | +1 |
| *P. lilfordi* | 0 | 0 | -- | -- |
| Seeds | 0 | 0 | -- | -- |
| Tysanura | 0 | 0 | -- | -- |
| Neuroptera | 0 | 0 | -- | -- |
| **Total** | **100** | **100** |  |  |

Table B48
