# Supplementary material for: Omnivory of an Insular Lizard: Sources of Variation in the Diet of Podarcis lilfordi (Squamata, Lacertidae)
Source: PLoS One. 2016 Feb 12;11(2):e0148947. doi: 10.1371/journal.pone.0148947 (PMC4752353; doi:10.1371/journal.pone.0148947)
Supplement: S49 Table — (DOCX) [file pone.0148947.s057.docx]

| **Taxon** | **%n**  **availability** | **%n diet** | **D** | **E** |
| --- | --- | --- | --- | --- |
| Gastropoda | 0 | 6.2176 | +1 | +1 |
| Pseudoscorpionida | 0 | 0.5181 | +1 | +1 |
| Araneae | 0 | 1.5544 | +1 | +1 |
| Acarina | 1.8182 | 0 | -1 | -1 |
| Isopoda | 0 | 1.0362 | +1 | +1 |
| Crustaceae | 0 | 0 | -- | -- |
| Diplopoda | 0 | 1.0362 | +1 | +1 |
| Orthoptera | 0 | 0 | -- | -- |
| Blattodea | 0 | 1.5544 | +1 | +1 |
| Isoptera | 0 | 1.5544 | +1 | +1 |
| Dermaptera | 0 | 2.0725 | +1 | +1 |
| Homoptera | 14.5454 | 12.4352 | -0.0903 | 0.1305 |
| Heteroptera | 0 | 2.5907 | +1 | +1 |
| Diptera | 23.6364 | 0.5181 | 0.9669 | -0.9355 |
| Lepidoptera | 0 | 1.0363 | +1 | +1 |
| Coleoptera | 18.1818 | 9.8445 | -0.3410 | -0.0968 |
| Hymenoptera | 7.2727 | 14.5078 | 0.3678 | 0.5041 |
| Formicidae | 16.3636 | 37.8238 | 0.5133 | 0.5570 |
| Unidentif. Arthrop. | 0 | 0 | -- | -- |
| Larvae | 18.1818 | 1.5544 | -0.8673 | -0.7699 |
| *P. lilfordi* | 0 | 0.5181 | +1 | +1 |
| Seeds | 0 | 3.6269 | +1 | +1 |
| Tysanura | 0 | 0 | -- | -- |
| Neuroptera | 0 | 0 | -- | -- |
| **Total** | **100** | **100** |  |  |

Table B49
