# Supplementary material for: Omnivory of an Insular Lizard: Sources of Variation in the Diet of Podarcis lilfordi (Squamata, Lacertidae)
Source: PLoS One. 2016 Feb 12;11(2):e0148947. doi: 10.1371/journal.pone.0148947 (PMC4752353; doi:10.1371/journal.pone.0148947)
Supplement: S50 Table — (DOCX) [file pone.0148947.s058.docx]

| **Taxon** | **%n**  **availability** | **%n diet** | **D** | **E** |
| --- | --- | --- | --- | --- |
| Gastropoda | 0 | 3.6290 | +1 | +1 |
| Pseudoscorpionida | 0 | 1.2097 | +1 | +1 |
| Araneae | 1.2820 | 20.0161 | 0.2261 | 0.0853 |
| Acarina | 0 | 0 | -- | -- |
| Isopoda | 1.2820 | 4.4355 | 0.5627 | 0.4460 |
| Crustaceae | 0 | 0 | -- | -- |
| Diplopoda | 0 | 0 | -- | -- |
| Orthoptera | 2.5641 | 0 | -1 | -1 |
| Blattodea | 0 | 5.6452 | +1 | +1 |
| Isoptera | 0 | 3.6290 | +1 | +1 |
| Dermaptera | 0 | 1.6129 | +1 | +1 |
| Homoptera | 34.6153 | 7.2580 | -0.7424 | -0.7268 |
| Heteroptera | 0 | 3.6290 | +1 | +1 |
| Diptera | 28.2051 | 0.8064 | -0.9594 | -0.9578 |
| Lepidoptera | 1.2820 | 0 | -1 | -1 |
| Coleoptera | 5.1282 | 4.0322 | -0.1253 | -0.2553 |
| Hymenoptera | 1.2820 | 20.1613 | 0.9022 | 0.8445 |
| Formicidae | 21.7949 | 39.9193 | 0.4090 | 0.1603 |
| Unidentif. Arthrop. | 0 | 0 | -- | -- |
| Larvae | 0 | 0.4032 | +1 | +1 |
| *P. lilfordi* | 0 | 0 | -- | -- |
| Seeds | 0 | 1.6129 | +1 | +1 |
| Tysanura | 2.5641 | 0 | -1 | -1 |
| Neuroptera | 0 | 0 | -- | -- |
| **Total** | **100** | **100** |  |  |

Table B50
