# Supplementary material for: Omnivory of an Insular Lizard: Sources of Variation in the Diet of Podarcis lilfordi (Squamata, Lacertidae)
Source: PLoS One. 2016 Feb 12;11(2):e0148947. doi: 10.1371/journal.pone.0148947 (PMC4752353; doi:10.1371/journal.pone.0148947)
Supplement: S51 Table — (DOCX) [file pone.0148947.s059.docx]

| **Taxon** | **%n**  **availability** | **%n diet** | **D** | **E** |
| --- | --- | --- | --- | --- |
| Gastropoda | 0.9009 | 1.2903 | 0.1796 | -0.0731 |
| Pseudoscorpionida | 0 | 3.8710 | +1 | +1 |
| Araneae | 0 | 1.2903 | +1 | +1 |
| Acarina | 0 | 0 | -- | -- |
| Isopoda | 0 | 0 | -- | -- |
| Crustaceae | 0 | 0 | -- | -- |
| Diplopoda | 0 | 0 | -- | -- |
| Orthoptera | 0 | 0 | -- | -- |
| Blattodea | 0 | 5.1613 | +1 | +1 |
| Isoptera | 0 | 0.6452 | +1 | +1 |
| Dermaptera | 0 | 0 | -- | -- |
| Homoptera | 0 | 1.2903 | 0.1796 | -00731 |
| Heteroptera | 8.5585 | 1.2903 | -0.7549 | -0.8333 |
| Diptera | 7.6576 | 1.9355 | -0.6155 | -0.7355 |
| Lepidoptera | 1.8018 | 2.5806 | 0.1816 | -0.0731 |
| Coleoptera | 0.4504 | 3.2258 | 0.7609 | 0.6239 |
| Hymenoptera | 1.3513 | 0.6452 | -0.3568 | -0.5529 |
| Formicidae | 77.9279 | 72.2580 | -0.1509 | -0.2827 |
| Unidentif. Arthrop. | 0 | 1.2903 | +1 | +1 |
| Larvae | 0 | 3.2258 | +1 | +1 |
| *P. lilfordi* | 0 | 0 | -- | -- |
| Seeds | 0 | 0 | -- | -- |
| Tysanura | 0 | 0 | -- | -- |
| Neuroptera | 0.4504 | 0 | -1 | -1 |
| **Total** | **100** | **100** |  |  |

Table B51
