# Supplementary material for: Omnivory of an Insular Lizard: Sources of Variation in the Diet of Podarcis lilfordi (Squamata, Lacertidae)
Source: PLoS One. 2016 Feb 12;11(2):e0148947. doi: 10.1371/journal.pone.0148947 (PMC4752353; doi:10.1371/journal.pone.0148947)
Supplement: S52 Table — (DOCX) [file pone.0148947.s060.docx]

| **Taxon** | **%n**  **availability** | **%n diet** | **D** | **E** |
| --- | --- | --- | --- | --- |
| Gastropoda | 2.3809 | 3.5191 | 0.1985 | -0.2806 |
| Pseudoscorpionida | 0 | 0 | -- | -- |
| Araneae | 0 | 0.5865 | +1 | +1 |
| Acarina | 0 | 0 | -- | -- |
| Isopoda | 0 | 4.1056 | +1 | +1 |
| Crustaceae | 0 | 0 | -- | -- |
| Diplopoda | 0 | 1.1730 | +1 | +1 |
| Orthoptera | 0 | 0 | -- | -- |
| Blattodea | 0 | 2.6393 | +1 | +1 |
| Isoptera | 0 | 0.8798 | +1 | +1 |
| Dermaptera | 0 | 0 | -- | -- |
| Homoptera | 14.2857 | 1.4663 | -0.8361 | -0.9249 |
| Heteroptera | 0 | 0.8798 | +1 | +1 |
| Diptera | 61.9048 | 0 | -1 | -1 |
| Lepidoptera | 2.3809 | 0 | -1 | -1 |
| Coleoptera | 4.7619 | 4.3988 | -0.0415 | -0.4803 |
| Hymenoptera | 4.7619 | 58.0645 | 0.9303 | 0.6450 |
| Formicidae | 7.1428 | 19.9413 | 0.5281 | 0.0296 |
| Unidentif. Arthrop. | 0 | 0 | -- | -- |
| Larvae | 0 | 0.5865 | +1 | +1 |
| *P. lilfordi* | 0 | 0.2932 | +1 | +1 |
| Seeds | 0 | 1.4663 | +1 | +1 |
| Tysanura | 2.3809 | 0 | -1 | -1 |
| Neuroptera | 0 | 0 | -- | -- |
| **Total** | **100** | **100** |  |  |

Table B52
