# Supplementary material for: Omnivory of an Insular Lizard: Sources of Variation in the Diet of Podarcis lilfordi (Squamata, Lacertidae)
Source: PLoS One. 2016 Feb 12;11(2):e0148947. doi: 10.1371/journal.pone.0148947 (PMC4752353; doi:10.1371/journal.pone.0148947)
Supplement: S53 Table — (DOCX) [file pone.0148947.s061.docx]

| **Taxon** | **%n**  **availability** | **%n diet** | **D** | **E** |
| --- | --- | --- | --- | --- |
| Gastropoda | 0 | 4.7059 | +1 | +1 |
| Pseudoscorpionida | 0 | 1.7647 | +1 | +1 |
| Araneae | 0 | 2.3529 | +1 | +1 |
| Acarina | 0 | 0.5882 | +1 | +1 |
| Isopoda | 13.8889 | 6.4706 | -0.3996 | -0.4835 |
| Crustaceae | 0 | 1.1765 | +1 | +1 |
| Diplopoda | 0 | 0 | -- | -- |
| Orthoptera | 5.5555 | 0 | -1 | -1 |
| Blattodea | 0 | 9.4118 | +1 | +1 |
| Isoptera | 0 | 4.7059 | +1 | +1 |
| Dermaptera | 0 | 0 | -- | -- |
| Homoptera | 0 | 7.0588 | +1 | +1 |
| Heteroptera | 0 | 2.3529 | +1 | +1 |
| Diptera | 30.5555 | 0.5882 | -0.9735 | -0.9716 |
| Lepidoptera | 2.7777 | 0.5882 | -0.6569 | -0.7267 |
| Coleoptera | 8.3333 | 7.0588 | -0.0896 | -0.2247 |
| Hymenoptera | 19.4444 | 8.8235 | -0.4276 | -0.4935 |
| Formicidae | 19.4444 | 39.4118 | 0.4587 | 0.2047 |
| Unidentif. Arthrop. | 0 | 0.5882 | +1 | +1 |
| Larvae | 0 | 0.5882 | +1 | +1 |
| *P. lilfordi* | 0 | 0 | -- | -- |
| Seeds | 0 | 1.7647 | +1 | +1 |
| Tysanura | 0 | 0 | -- | -- |
| Neuroptera | 0 | 0 | -- | -- |
| **Total** | **100** | **100** |  |  |

Table B53
